# Global, regional, and national burden of migraine in postmenopausal women: a 32-year analysis from the global burden of disease study 1990–2021

**DOI:** 10.22514/jofph.2026.052

**Published:** 2026-07-12

**Authors:** Yufeng Ding, Kegang Cao, Nannan Qian, Yulong Yang, Xuran Zhang

**Affiliations:** ^1^Department of Neurology, Dongzhimen Hospital of Beijing University of Chinese Medicine, 100700 Beijing, China; ^2^Department of Neurology, the First Affiliated Hospital of Anhui University of Chinese Medicine, 230031 Hefei, Anhui, China

**Keywords:** Migraine, Postmenopausal women, Global burden of disease 2021, Age-standardized rates, Socioeconomic inequality

## Abstract

**Background**: Migraine is a major cause of disability globally, yet its epidemiological impact on postmenopausal women (aged ≥55 years) is not well understood. This study aims to fill this knowledge gap by analyzing long-term trends in migraine burden from 1990 to 2021. **Methods**: Data from the Global Burden of Disease (GBD) 2021 study were utilized to estimate prevalence, incidence, and disability-adjusted life years (DALYs), stratified by age group, Socio-demographic Index (SDI), and geographical region. Temporal trends were assessed using the estimated annual percentage change (EAPC), while socioeconomic inequalities were evaluated through the Slope Index of Inequality (SII) and Concentration Index (CIX). **Results**: The number of prevalent cases of migraine among postmenopausal women increased by 118.3% globally, rising from 55.29 million to 120.68 million between 1990 and 2021. Age-standardized rates (ASRs) in this population displayed contrasting trends: while age-standardized prevalence and incidence rates experienced slight increases (EAPC: 0.07–0.17), the age-standardized DALY rate (ASDR) remained stable. Middle-SDI regions faced the highest absolute burden of migraine in postmenopausal women and showed the most rapid growth in ASRs. Inequality analyses highlighted persistent socioeconomic disparities, revealing significant efficiency gaps in health resource utilization in high-SDI countries. **Conclusions**: This study highlights the increasing absolute burden of migraine among postmenopausal women, despite stable age-standardized rates. This underscores the need for context-specific interventions across SDI strata to reduce health inequities and optimize resource allocation in migraine care.

## 1. Introduction

Migraine is a prevalent and disabling primary headache disorder marked by recurrent episodes of moderate-to-severe throbbing pain, frequently accompanied by sensory sensitivities. It continues to be a leading cause of disability globally, placing a significant burden on healthcare systems and affecting quality of life [1]. While extensive epidemiological research has characterized the global distribution of migraine, significant gaps remain in understanding its specific burden and long-term trends among older adults [2], especially postmenopausal women, a growing demographic group particularly vulnerable to chronic health conditions [3].

Existing studies have primarily focused on migraine in reproductive-age populations or general adult cohorts. However, they often overlook the distinct epidemiological patterns that may emerge after menopause [4, 5]. These patterns may involve differences in incidence, symptom severity, disability burden, or trigger factors, which are not yet fully elucidated. Furthermore, the evolving global distribution of migraine burden across various socioeconomic development levels remains insufficiently explored, especially through a sex- and age-specific lens. Understanding how demographic shifts, aging populations, and disparities in healthcare resources—such as diagnostic capacity, access to preventive treatments, and specialist care—affect the burden of migraine in postmenopausal women is essential for effective resource allocation and targeted public health interventions.

This study aims to fill these evidence gaps by providing a comprehensive assessment of the global, regional, and national burden of migraine in postmenopausal women from 1990 to 2021. Utilizing data from the Global Burden of Disease (GBD) 2021 study, we analyze trends in prevalence, incidence, and disability-adjusted life years (DALYs) across socio-demographic index (SDI) strata, geographic regions, and countries [6]. We further analyze age-specific patterns and conduct inequality assessments to identify disparities in disease distribution and healthcare efficiency.

Our analysis provides a nuanced perspective on the evolution of migraine burden over three decades within this specific population subgroup. This evidence can inform clinical practice and health policy development tailored to the needs of postmenopausal women across various developmental contexts.

## 2. Materials and methods

### 2.1 Data source and study population

Data were obtained from the Global Burden of Diseases (GBD) 2021 study, which offers comprehensive estimates of the burden of 371 diseases and injuries across 204 countries and territories from 1990 to 2021. We defined postmenopausal women as those aged 55 years or older. Although the average age of natural menopause typically ranges from 45 to 55 years globally, we chose a cutoff of 55 years to more conservatively identify a population that is clearly beyond the menopausal transition. This approach helps to reduce misclassification and enhances the biological specificity of our postmenopausal cohort [7]. Migraine was identified according to the International Classification of Headache Disorders, 3rd edition (ICHD-3), and corresponding International Classification of Diseases (ICD) codes: ICD-9 (346–346.93) and ICD-10 (G43–G43.919) [8].

### 2.2 Metrics and estimation

We extracted three primary metrics: prevalence, incidence, and disability-adjusted life years (DALYs). DALYs are a combination of years of life lost (YLLs) and years lived with disability (YLDs). Because migraine is non-fatal, we considered YLLs to be zero, meaning that DALYs equal YLDs [9]. All estimates were generated with 95% uncertainty intervals (UIs) using 500 draws to propagate uncertainty throughout the modeling process.

### 2.3 Socio-demographic index (SDI)

The SDI is a composite measure of development that considers average income per capita, educational attainment, and total fertility rate. Countries were classified into five SDI quintiles: low, low-middle, middle, high-middle, and high. Additionally, we analyzed 21 GBD regions and 204 countries/territories to examine geographic variations [10].

### 2.4 Statistical analysis

Temporal trends were evaluated using the estimated annual percentage change (EAPC), calculated from a linear regression model applied to the natural logarithm of age-standardized rates (ASRs). A positive EAPC with a 95% confidence interval (CI) that does not include zero indicates an increasing trend, whereas a negative EAPC indicates a decreasing trend [11]. ln(ASR) is the natural logarithm of the age-standardized rate, α is the intercept, β is the slope coefficient representing the average annual change in the log-transformed rate, Year is the calendar year, and ε is the error term. The formula is as follows (Eqn. 1):

$$\begin{array}{cr} ln(ASR)=\alpha +\beta \times \text{Year}+\varepsilon & \;\text{(1)} \end{array}$$

The EAPC was then derived from the slope coefficient β using the formula as follows (Eqn. 2):

$$\begin{array}{cr} EAPC=100\times (e^{\beta }-1) & \;\text{(2)} \end{array}$$

Inequality was evaluated using the Slope Index of Inequality (SII) and Concentration Index (CIX), which measure absolute and relative socioeconomic disparities in health outcomes, respectively [12]. Frontier analysis was conducted to compare observed DALY rates with theoretically achievable rates given a country’s SDI, identifying efficiency gaps [13]. Decomposition analysis quantified contributions of population growth, aging, and epidemiological changes to DALY trends [14].

All analyses were performed using R software version 4.2.2. The figures were generated using the JD_GBDR (V2.5.7, provided by Jingding Medical Technology Co., Ltd., Hefei, China).

## 3. Results

### 3.1 Global level

Global data show a significant increase in the burden of migraine among postmenopausal women from 1990 to 2021. The number of prevalent cases among postmenopausal women rose from 55.29 million to 120.68 million, representing a 118.26% increase. Similarly, incident cases among postmenopausal women increased from 1.60 million to 3.60 million, a rise of 125.15%. Disability-adjusted life years (DALYs) due to migraine in postmenopausal women also increased by 113.86%, from 2.13 million to 4.56 million. Age-standardized rates (ASRs) displayed differing trends: the age-standardized prevalence rate (ASPR) increased from 15,194.93 to 15,394.20 per 100,000 population (estimated annual percentage change (EAPC) = 0.07; 95% confidence interval (CI): 0.05–0.09), while the age-standardized incidence rate (ASIR) rose from 440.67 to 460.29 per 100,000 (EAPC = 0.17; 95% CI: 0.15–0.18), both indicating statistically significant upward trends. In contrast, the age-standardized DALY rate (ASDR) remained stable, slightly declining from 585.68 to 582.08 per 100,000 (EAPC = 0.01; 95% CI: 0.00–0.03), with the confidence interval including zero, suggesting no significant temporal trend.

These findings underscore the growing global burden of migraine among postmenopausal women in terms of prevalence and incidence, alongside a persistently high level of disability (**Supplementary Table 1**, Fig. 1).

**Fig. 1.** Analysis of trends in the prevalence, incidence, and disability-adjusted life years (DALYs) of migraine among postmenopausal women worldwide from 1990 to 2021. (A) Prevalence. (B) Incidence. (C) DALYs. SDI: socio-demographic index.

### 3.2 Regional level

From 1990 to 2021, the burden of migraine among postmenopausal women exhibited pronounced regional heterogeneity—both across SDI strata and among the 21 GBD regions. At the SDI level, the middle SDI stratum bore the greatest absolute burden in 2021: prevalent cases reached 38.85 million (a 182.68% increase from 1990), incident cases totaled 1.26 million (a 178.05% increase), and DALYs amounted to 1.44 million (a 180.93% increase). The low-middle SDI region followed closely in absolute growth, with prevalence, incidence, and DALYs rising by 146.74%, 145.01%, and 146.28%, respectively. In contrast, the high SDI region experienced comparatively modest absolute increases—prevalence rose by 72.29%, incidence by 75.80%, and DALYs by 69.57%—underscoring a decoupling between socioeconomic development and absolute increases in disease. However, high SDI regions maintained the highest ASPR in 2021 (15,839.66 per 100,000; EAPC = 0.07; 95% CI: 0.03–0.11) and the second-highest ASDR of 604.57 per 100,000 (EAPC = 0.02; 95% CI: −0.01 to 0.05); moreover, neither trend achieved statistical significance (*p* > 0.05), suggesting a plateauing burden after decades of demographic and health transition. Middle SDI regions demonstrated the most rapid and statistically robust increases across all age-standardized indicators: ASPR rose significantly (EAPC = 0.16; 95% CI: 0.13–0.18), as did ASIR (EAPC = 0.11; 95% CI: 0.08–0.13) and ASDR (EAPC = 0.14; 95% CI: 0.12–0.16)—indicating accelerating disease pressure coinciding with ongoing epidemiological transition. Conversely, low–middle SDI regions exhibited consistent, statistically significant declines in all age-standardized rates—despite steep absolute growth—reflecting population aging and expanding case detection outpacing true increase in incidence: ASPR declined (EAPC = −0.03; 95% CI: −0.03 to −0.02), ASIR fell (EAPC = −0.03; 95% CI: −0.03 to −0.02), and ASDR decreased (EAPC = −0.02; 95% CI: −0.02 to −0.01).

At the GBD regional level, absolute burden increased universally—but the magnitude and underlying drivers diverged markedly. East Asia recorded one of the largest absolute expansions: prevalence surged by 184.98% (from 10.63 million to 30.30 million), incidence by 175.63% (from 0.40 million to 1.11 million), and DALYs by 182.37% (from 0.39 million to 1.11 million). Central Latin America reported a 224.51% rise in prevalence (from 1.07 million to 3.46 million), while Andean Latin America showed a 215.86% increase (from 0.19 million to 0.58 million). Critically, age-standardized trends were not merely corollaries of absolute growth: East Asia exhibited significant, parallel increases across all three standardized metrics—ASPR (EAPC = 0.40; 95% CI: 0.33–0.47), ASIR (EAPC = 0.26; 95% CI: 0.21–0.30), and ASDR (EAPC = 0.36; 95% CI: 0.29–0.42)—pointing to a genuine escalation in underlying risk or diagnostic intensity. A similar pattern emerged in Andean Latin America (ASPR: EAPC = 0.24; ASIR: 0.13; ASDR: 0.18), whereas Southeast Asia displayed a contrasting decline in both ASPR (EAPC = −0.06; 95% CI: −0.08 to −0.04) and ASDR (EAPC = −0.05; 95% CI: −0.07 to −0.03), suggesting successful mitigation or shifting comorbidity profiles. Tropical Latin America uniquely showed a selective decline in ASIR only (EAPC = −0.03; 95% CI: −0.05 to −0.02), implying region-specific influences on new-onset disease.

High-income regions displayed notable intra-group divergence. Western Europe showed a statistically significant rise in ASPR (EAPC = 0.09; 95% CI: 0.07–0.12), whereas high-income North America showed a non-significant decline in ASDR (EAPC = −0.04; 95% CI: −0.10 to 0.03)—highlighting differential impacts of healthcare access, aging structure, or diagnostic practices. Australasia, Oceania, and South Asia exhibited stability across all standardized indicators (EAPCs ≈ 0, with 95% CIs spanning zero), indicating no detectable temporal shift in underlying burden intensity. Collectively, these patterns reveal a dual epidemiological reality: middle SDI and several transitioning regions face escalating age-standardized burden—signaling an emergent public health priority—while select high SDI settings sustain persistently high ASDR despite minimal growth, reflecting an entrenched disease burden requiring sustained service investment (**Supplementary Table 1**; **Supplementary Fig. 1**).

### 3.3 Countries level

Between 1990 and 2021, the global burden of migraine among postmenopausal women showed significant yet varied changes across 204 countries and territories. Approximately 99% of these jurisdictions experienced increases in prevalence (202/204), incidence (201/204), and disability-adjusted life years (DALYs) (201/204). Qatar had the highest percentage increases in prevalence (880.61%), incidence (879.75%), and DALYs (862.87%). In contrast, Georgia experienced the steepest declines across all three metrics, with decreases of −11.22%, −12.42%, and −12.65%, respectively.

Age-standardized rates (ASRs) revealed differing epidemiological patterns. The proportion of countries with increasing age-standardized DALY rates (ASDRs) for migraine in postmenopausal women was notably higher (31.86%; 65/204) compared to those with rising age-standardized prevalence rates (ASPRs) (13.73%; 28/204) or age-standardized incidence rates (ASIRs) (9.80%; 20/204). Belgium demonstrated the largest increases in ASPR (16.45%) and ASDR (13.61%), while Thailand experienced the most significant declines in both ASPR (−11.66%) and ASDR (−10.65%) (**Supplementary Tables 2,3,4**, Fig. 2).

**Fig. 2.** Age-standardized rates of migraine among postmenopausal women across 204 countries and territories worldwide in 2021. (A) ASPR. (B) ASIR. (C) ASDR. ASPR: age-standardized prevalence rate; ASIR: age-standardized incidence rate; ASDR: age-standardized disability-adjusted life years rate.

Estimated annual percentage change (EAPC) analysis revealed that Belgium exhibited the most substantial upward trends in ASPR (EAPC = 0.53; 95% confidence interval (CI): 0.38–0.69) and ASDR (EAPC = 0.45; 95% CI: 0.32–0.58). Conversely, Thailand recorded the steepest declines in ASPR (EAPC = −0.43; 95% CI: −0.56 to −0.31) and ASDR (EAPC = −0.39; 95% CI: −0.51 to −0.28). Notable increases in ASIR were observed in Japan (EAPC = 0.40; 95% CI: 0.33–0.48) and China (EAPC = 0.27; 95% CI: 0.22–0.32), contributing to the rising migraine burden in East Asia. This trend stands in stark contrast to the consistent declines noted in Egypt, where ASPR (EAPC = −0.24; 95% CI: −0.29 to −0.20), ASIR (EAPC = −0.12; 95% CI: −0.14 to −0.09), and ASDR (EAPC = −0.22; 95% CI: −0.26 to −0.19) all decreased significantly (**Supplementary Tables 2,3,4**, **Supplementary Fig. 2**). It should be noted that national-level variation in case counts and age-standardized rates may be influenced by differences in healthcare access, diagnostic capacity, and case ascertainment methods across countries, in addition to true differences in disease occurrence.

### 3.4 Age patterns

Migraine prevalence, incidence, and disability-adjusted life years (DALYs) among postmenopausal women showed a consistent decline with advancing age from 1990 to 2021. The greatest burden was found in the 55–59-year age group across all metrics, while the ≥95-year age group exhibited the lowest values globally. This inverse age-related gradient was consistent across all Socio-demographic Index (SDI) strata and age cohorts.

A slight deviation was noted in high-SDI regions, where incidence proportions in three consecutive age groups (70–74, 75–79, and 80–84 years) were marginally higher in older cohorts than in adjacent younger groups. For example, in 2021, the incidence proportion for the 80–84-year group (10.7%) slightly surpassed that of the 70–74-year group (9.8%), indicating a reversal of the overall trend. Despite this anomaly, all four age groups maintained incidence proportions close to 10% throughout the study period.

Overall, the migraine burden among postmenopausal women demonstrated a consistent inverse association with age from 1990 to 2021. The 55–59-year cohort consistently exhibited the highest global levels of prevalence, incidence, and DALYs, confirming a progressive decline in disease burden with increasing age. Nonetheless, all age groups had non-negligible case proportions, emphasizing the importance of expanding clinical attention beyond reproductive-age women to include postmenopausal populations (Fig. 3 and **Supplementary Figs 3,4,5**).

**Fig. 3.** Comparative analysis of the prevalence, incidence, and burden of migraine among postmenopausal women across different age groups worldwide in 2021. (A) Prevalence. (B) Incidence. (C) DALYs. UI: uncertainty interval.

### 3.5 Cross-country inequality analysis, frontier analysis and decomposition analysis

Socioeconomic inequality in migraine burden among postmenopausal women has consistently favored higher socioeconomic groups from 1990 to 2021. The Slope Index of Inequality (SII) for migraine in postmenopausal women rose from 93.27 (95% confidence interval (CI): 67.01–119.53) to 97.47 (95% CI: 72.16–122.79), indicating a widening absolute disparity in disability-adjusted life years (DALYs) in this population favoring wealthier groups. Meanwhile, the Concentration Index (CIX) remained slightly positive and stable throughout this period (1990: 0.034; 2021: 0.036; 95% CIs excluding zero), showing minimal change in the relative socioeconomic gradient. This trend confirms that while relative inequalities in migraine burden among postmenopausal women have remained largely stable, the absolute burden gap has significantly expanded (Fig. 4).

**Fig. 4.** Health inequality regression curves (A) and concentration curves (B) for the DALYs of migraine among postmenopausal women worldwide, 1990 and 2021. SDI: socio-demographic index; SII: Slope Index of Inequality; CI: confidence interval.

Frontier analysis of the migraine burden across 204 countries and territories uncovered considerable unrealized health potential in managing this condition among postmenopausal women, especially in high-income societies. High-SDI nations (SDI >0.8) showed the largest efficiency gaps in reducing the ASDR for migraine among postmenopausal women. For instance, Belgium’s observed ASDR for migraine in postmenopausal women was 855.62 per 100,000 population in 2021, which was 512.27 points higher than its theoretical optimum of 343.35. Similarly large disparities in ASDR for migraine among postmenopausal women were noted in Germany (difference: 380.26) and Canada (difference: 334.50), representing approximately 300–500 avoidable DALYs per 100,000 in this population despite their high resource availability. In contrast, low-SDI countries such as Somalia (efficiency difference = 0) and Ethiopia (5.66) operated close to their efficiency frontiers for managing migraine in postmenopausal women, achieving near-maximal performance given their developmental constraints. Notably, efficiency gaps in achieving low ASDR for migraine among postmenopausal women exhibited an inverted U-shaped relationship with SDI, with upper-middle-SDI regions (*e.g.*, Russia, Ukraine) showing the greatest disparities (350–450 per 100,000), surpassing those in both lower- and higher-SDI regions (Fig. 5).

**Fig. 5.** Frontier analysis of the relationship between the Socio-demographic Index (SDI) and disability-adjusted life years (DALYs) for migraine among postmenopausal women in 204 countries and territories. (A) The color gradient from light blue (1990) to dark blue (2021) indicates temporal change. (B) Each point represents a specific country or territory in 2021. The frontier line is displayed in black, with the 15 countries and territories exhibiting the greatest deviations from the frontier highlighted. Blue denotes low-SDI regions with minimal deviation from the frontier, while red signifies high-SDI regions with the largest gaps. The direction of age-standardized DALY rate (ASDR) change between 1990 and 2021 is represented by dot color: red indicates a decrease, and green indicates an increase. SDI: socio-demographic index.

A decomposition analysis was performed to quantify the contributions of population growth, population aging, and epidemiological change to the evolution of migraine-related DALYs among postmenopausal women between 1990 and 2021, stratified by Socio-demographic Index (SDI). Globally, the increase of 2.43 million DALYs due to migraine in postmenopausal women was predominantly driven by population growth, which accounted for 102.97% of the net change. Changes in age structure had a protective effect on the migraine burden in this population, contributing −2.12%, while epidemiological changes slightly reduced the burden (−0.85%).

Distinct patterns in the drivers of DALYs for migraine among postmenopausal women emerged across the SDI spectrum. Population growth was the primary driver of increasing DALYs in this population across all regions, with contributions ranging from 98.38% to 108.28%. The high-middle SDI region experienced the most significant increase attributed to growth, with population expansion explaining 108.28% of the change in DALYs. The protective effect of aging was most pronounced in high-SDI regions (−5.00%), followed by smaller but consistent reductions in other regions. Epidemiological changes generally contributed to a reduction in disease burden across most regions, except in the middle-SDI region, where they had a negative impact due to rising epidemiologic rates (contribution: +3.82%). The analysis revealed a clear gradient in the factors contributing to changes in migraine-related DALYs among postmenopausal women along the SDI continuum. In low-SDI regions, changes in DALYs for migraine among postmenopausal women were almost entirely attributable to population growth (100.65%), with negligible contributions from other factors (<1%). In contrast, high-SDI regions showed more complex interactions among all three drivers (**Supplementary Fig. 6**).

## 4. Discussion

This study offers the first comprehensive assessment of the global, regional, and national burden of migraine among postmenopausal women aged 55 years and older from 1990 to 2021. Our findings highlight a complex epidemiological landscape for migraine in postmenopausal women, marked by a dual burden: while there has been a substantial increase in absolute case counts and disability-adjusted life years (DALYs), age-standardized rates have remained remarkably stable over the 32-year period. This trend, along with significant heterogeneity across Socio-demographic Index (SDI) regions, geographical areas, and age groups, provides essential insights into the changing epidemiology of migraine in this population and guides future public health strategies.

A central finding is the significant increase in the absolute burden of migraine among postmenopausal women worldwide. During the study period, the number of prevalent cases, incident cases, and total DALYs more than doubled. This increase underscores the rising clinical and healthcare system burden associated with this neurological disorder in the growing population of aging women. In contrast, the age-standardized DALY rate (ASDR) remained relatively stable, showing only a slight change from 582.08 to 585.68 per 100,000 population, with an estimated annual percentage change close to zero.

However, interpreting these trends requires careful consideration of diagnostic opportunity and healthcare access. Migraine remains substantially underdiagnosed globally, particularly in older adults and in lowresource settings. The observed increases in case numbers—especially in high SDI regions and among women aged 70+ years—may partly reflect improved case ascertainment rather than a true rise in incidence. Postmenopausal women have frequent contact with healthcare systems for chronic non-communicable diseases, creating more opportunities for incidental migraine diagnosis, particularly in high SDI regions and among those aged 70+ years. Conversely, the persistently low age-standardized rates in low- and lower-middle-SDI regions may mask a substantial hidden burden. This disparity is likely attributable to limited access to neurologists, low public awareness, and competing health priorities in these settings.

Thus, the epidemiological patterns we report represent a composite of true disease occurrence, diagnostic intensity, and healthcare system capacity. Two concurrent trends help reconcile these observations. First, the increase in absolute numbers is mainly driven by global population growth and demographic aging—key contributors to the rising burden of chronic non-communicable diseases [15]. Second, improved diagnostic accuracy, heightened public awareness, and revisions in diagnostic criteria have likely enhanced the identification of cases that were previously underdiagnosed or misclassified [16].

The stabilization of the age-standardized rate likely reflects distinct mechanisms across settings. The contribution of novel, high-cost therapies such as calcitonin gene-related peptide (CGRP) monoclonal antibodies to this trend is likely minimal at the population level, given their current limited accessibility even within high-SDI countries [17, 18, 19]. For low- and middle-SDI regions, where access to such advanced therapies is exceedingly rare, a stable ASDR might paradoxically reflect systemic constraints and persistent care gaps: limited healthcare capacity may lead to underdiagnosis of milder cases, while the most severe cases presenting for care are those incurring significant disability. Second, methodological aspects of the GBD study—such as the use of modeling tools (DisMod-MR 2.1) that harmonize data across time and regions—may inherently produce smoothed estimates that further stabilize the age-standardized metrics [20, 21].

Therefore, the observed stabilization represents a complex equilibrium between genuine clinical improvements in some settings, methodological smoothing, and persistent under-ascertainment in others—rather than a uniform global success in migraine therapy.

The middle SDI region has become the epicenter of the global migraine burden for postmenopausal women. This region reported not only the highest absolute number of DALYs but also the most rapid growth in both absolute case counts and age-standardized rates (ASRs). This trend likely results from a combination of factors. The rapid growth in absolute burden in middle-SDI regions coincides with periods of intense socioeconomic transition. These transitions may increase population exposure to several modifiable migraine risk factors. For instance, urbanization is often associated with changes in dietary patterns, occupational stress, sleep hygiene, and environmental exposures (*e.g.*, air pollution) [22, 23]. At the same time, many middle-SDI countries have healthcare systems that may be insufficiently equipped to meet the rising demand for services. This shortfall results in a widening gap between increasing risk exposure and the availability of timely, high-quality prevention, diagnosis, and treatment options.

High-SDI regions consistently reported the highest age-standardized prevalence and DALY rates. While this may reflect a genuinely higher disease burden, it is also plausible that these regions have approached diagnostic saturation. With near-universal healthcare coverage, high neurologist density, and long-standing public awareness campaigns, most individuals with migraine who seek care are likely already diagnosed. In this context, the minimal growth in ASRs may not indicate successful prevention of new cases, but rather that the pool of undiagnosed cases has been largely exhausted. This contrasts with middle-SDI regions, where rising ASRs may signify a transition from underdiagnosis toward fuller case identification, concurrent with true risk factor exposure. These disparate patterns suggest that the global epidemiology of migraine is characterized not by a uniform trajectory, but by distinct phases that correspond to healthcare system capacity and diagnostic infrastructure.

From a clinical perspective, the near-saturation of migraine diagnosis in high-SDI regions shifts the focus from case identification to disease management in an aging population. The rising absolute number of older adults with migraine—particularly women aged 70+ years—introduces new complexities, including multimorbidity (*e.g.*, cardiovascular diseases and neurocognitive disorders) and the associated diagnostic and therapeutic challenges. In these settings, reducing migraine-related disability increasingly depends not on expanding access to diagnosis, but on integrating headache care into the management of multiple chronic conditions [24, 25].

In addition to pharmacological management, non-pharmacological interventions such as physiotherapy and dietary therapy can play a valuable role in improving the quality of life for women with migraine. Physiotherapy, including manual therapy and exercise programs, may help address musculoskeletal contributors to headache, such as cervical dysfunction or muscle tension, which are common in older adults [26]. Dietary modifications—including identification of individual trigger foods, regular meal timing, and adequate hydration—are simple, low-cost strategies that can complement medical treatment and reduce attack frequency [27]. These approaches are particularly relevant for postmenopausal women, who may experience changes in headache patterns and seek non-drug options due to concerns about polypharmacy or medication interactions. Integrating such lifestyle-based interventions into routine migraine care, regardless of SDI setting, can enhance patient empowerment and contribute to better long-term outcomes.

The observed decline in age-standardized rates in low-middle-SDI regions requires careful interpretation. One optimistic view is that this reflects genuine improvements in population health or successful public health interventions [28]. However, an alternative and equally plausible explanation is persistent or even worsening underdiagnosis. In many low-middle-SDI countries, health systems are strained by the dual burden of communicable and non-communicable diseases, and headache disorders are often deprioritized. Limited access to specialized neurological care, a shortage of headache-trained clinicians, and low public awareness that migraine is a treatable medical condition may result in a large and potentially growing reservoir of undiagnosed cases that are not captured in administrative or survey data. The true burden in these regions is therefore likely substantially higher than current GBD estimates suggest.

Regionally, the most rapid increase in absolute disability-adjusted life years (DALYs) occurred in Southeast Asia and Andean Latin America. These areas have experienced profound socioeconomic transformations, including rapid urbanization, economic development, and a shift toward Westernized lifestyles. Such macro-level changes can lead to individual-level exposures, including psychological stress and environmental triggers like air pollution, creating a confluence of risk factors that contribute to higher migraine incidence [29]. The established link between PM2.5 exposure and increased healthcare utilization for headaches suggests a plausible biological and environmental mechanism [30, 31].

Southeast Asia’s status as an outlier, characterized by a declining age-standardized rate (ASR), requires further investigation. In addition to concerns about data quality, unmeasured cultural or social protective factors—such as the use of traditional remedies, robust community support networks, or specific dietary patterns—may influence this trend. Understanding these factors could help in developing innovative, low-cost public health interventions [32, 33]. Divergent trends were observed among high-income regions: Western Europe experienced an increasing ASR, while North America saw a decline. This disparity likely reflects differences in healthcare system structures, pharmaceutical policies, patient behaviors, and investment in preventive care. These factors underscore the significant impact of specific health policies on long-term disease burden trajectories, even among countries with similar economic development.

The extreme national-level trends observed—such as the dramatic percentage increase in Qatar and the notable decrease in Georgia—must be interpreted with caution, as they are particularly susceptible to denominator effects and demographic volatility. Qatar has experienced one of the world’s most rapid population expansions, driven by large-scale labor migration. This influx substantially alters the age and sex structure of the population, and when calculating percentage increases over three decades, the small baseline population in 1990 can produce exceptionally high relative growth figures [34]. In Georgia, the opposite demographic trend—substantial population decline and emigration—may artificially deflate long-term growth estimates. Furthermore, in both settings, changes in healthcare infrastructure and diagnostic capacity over the study period likely influenced case detection rates independently of true disease incidence. These cases highlight the importance of interpreting percentage changes alongside absolute numbers and age-standardized rates, and caution against over-interpreting extreme values in demographically dynamic populations. Meanwhile, the rapid rise in ASR in Belgium, a highly aged and high-income nation, could suggest either growing challenges in managing migraines among multimorbid elderly populations or improvements in case detection and reporting accuracy for older adults [35].

The atypical plateau or slight increase in incidence among women aged 70–84 years in high-SDI regions—a pattern that diverges from the conventional post-reproductive decline observed globally [36]—has several potential explanations. While diagnostic challenges and misattribution of headache symptoms to medication side effects or comorbidities remain important considerations [37], this pattern may also reflect increased diagnostic opportunity. Older adults in high-income settings have frequent and regular contact with primary care physicians and specialists for the management of cardiovascular disease, diabetes, osteoarthritis, and other age-related conditions. Each encounter represents an opportunity for headache symptoms to be elicited, evaluated, and formally diagnosed. Thus, the observed plateau may partially represent the cumulative ascertainment of migraine in a population with high healthcare utilization, rather than a true increase in age-specific incidence. This finding reinforces the need for age-inclusive diagnostic guidelines that do not assume migraine declines universally after the seventh decade.

Analysis of socioeconomic inequality in this population has revealed a persistent and slightly widening absolute gap, as indicated by the increasing Slope Index of Inequality (SII). This situation presents a wealth-disease paradox: populations in high-SDI countries face a disproportionately high relative burden despite having superior healthcare resources. To address this disparity, policies must focus on equitable access, including strengthening primary care, expanding health insurance coverage, and disseminating standardized clinical guidelines to ensure effective migraine care reaches all socioeconomic groups.

The study also identified a significant efficiency gap in health resource utilization. Several high-SDI countries have considerable room for improvement, suggesting that high healthcare expenditure does not always lead to optimal health outcomes. In contrast, some low-SDI settings operate close to their efficiency frontier despite severe resource constraints, indicating that achieving basic service accessibility can yield high marginal returns in these contexts. For low- and middle-income countries, scaling up cost-effective interventions—such as patient education, self-management support, and ensuring the availability of essential, affordable medications—is critical for improving overall health system performance [26].

Decomposition analysis of the factors contributing to the increase in total DALYs revealed that population growth was the primary driver, responsible for over 100% of the net change. In contrast, population aging and epidemiological changes had a modest counteracting effect. This finding has significant implications for health resource planning, as the main pressure stems from the rising number of patients due to population expansion. However, ongoing efforts to lower age-standardized rates through chronic disease prevention and control are crucial for enhancing population health outcomes [38]. Future public health strategies must balance addressing the “quantity” of demand with enhancing the “quality” of chronic disease management.

A key strength of this study is its comprehensive scope and detailed analytical depth. It leverages the Global Burden of Disease (GBD) 2021 Study dataset to provide a systematic, long-term assessment of migraine burden in postmenopausal women, a population that has historically been underrepresented in epidemiological research.

However, this study has several limitations. First, as a model-based ecological analysis, its findings are constrained by the inherent limitations of GBD estimates. In many low- and middle-income regions, the lack of high-quality primary epidemiological data necessitates a heavy reliance on statistical modeling, which may not fully capture local disease dynamics [39]. Second, despite rigorous adjustments by GBD collaborators for data heterogeneity, variations in diagnostic criteria, case definitions, and data collection methods across different time periods and regions can impact cross-national comparability. Third, there is a significant global gap in high-quality, prospective studies that specifically examine migraine trends in postmenopausal women [40]. Fourth, our findings are subject to diagnostic ascertainment bias, which likely varies substantially across regions and over time. The GBD estimates rely on primary data from surveys, administrative records, and clinical registries, all of which are sensitive to healthcare-seeking behavior, clinician awareness, and the availability of diagnostic services. In regions with limited access to neurological care, many migraine cases—particularly those of moderate severity—may never be formally diagnosed or recorded, leading to systematic underestimation of the burden. Conversely, in high-income settings with high healthcare utilization among older adults, incidental diagnosis may inflate apparent prevalence and incidence among postmenopausal women. These competing biases preclude direct comparison of absolute rates across settings and underscore that the observed trends reflect both true epidemiological change and evolving patterns of medical recognition. Our analysis is constrained by the availability and representativeness of the underlying data. As such, these results should be viewed as the best synthesis of current evidence and as a strong call for targeted primary data collection.

## 5. Conclusions

In conclusion, the global burden of migraine in postmenopausal women is at a critical turning point. The ongoing increase in absolute case numbers presents a substantial challenge for healthcare systems worldwide. However, the stabilization of age-standardized rates offers hope, indicating that effective interventions can reduce individual-level disability. Our findings underscore the necessity for differentiated approaches to managing migraine in postmenopausal women across regions. The distinct profiles observed—from managing comorbidities in high-SDI regions, to addressing transitional health risks in middle-SDI regions, and overcoming barriers to care in low-SDI regions—highlight specific targets for health system planning and future research. Addressing these context-specific challenges through evidence-informed strategies is paramount to mitigating the growing global health impact of migraine in postmenopausal women.

## Supplementary Material

Supplementary material associated with this article can be found, in the online version, at https://files.jofph.com/files/article/2075461167256354816/attachment/Supplementary%20material.docx.


