# Supplementary material for: Global, regional, and national burden of migraine in postmenopausal women: a 32-year analysis from the global burden of disease study 1990–2021
Source: J Oral Facial Pain Headache. 2026 Jul 12;40(4):97–109. doi: 10.22514/jofph.2026.052 (PMC13434146; doi:10.22514/jofph.2026.052)
Supplement: Supplementary file 1 [file Supplementary-material.docx]

Supplementary material

Supplementary Table 1. Global and regional prevalence, incidence, and disability-adjusted life years (DALYs) of migraine in postmenopausal women from 1990 to 2021.

| Location | | 1990 | | 2021 | | EAPC (95% CI) |
| --- | --- | --- | --- | --- | --- | --- |
|  |  | Number (95% UI) | ASR (95% UI) | Number (95% UI) | ASR (95% UI) |  |
| Prevalence | | | | | | |
|  | Global | 55,289,061.89 (44,329,906.90, 70,364,037.37) | 15,194.93 (12,180.03, 19,344.24) | 120,675,003.70 (96,142,462.01, 154,190,395.53) | 15,394.20 (12,267.01, 19,666.57) | 0.07 (0.05, 0.09) |
|  | High SDI | 15,669,527.36 (12,451,729.79, 20,125,230.59) | 15,643.50 (12,464.52, 20,066.76) | 26,996,451.12 (21,422,826.39, 34,644,469.23) | 15,839.66 (12,611.25, 20,280.75) | 0.07 (0.03, 0.11) |
|  | High-middle SDI | 15,228,737.73 (12,179,778.75, 19,226,974.16) | 15,535.31 (12,424.66, 19,620.56) | 29,057,167.61 (23,221,071.74, 36,984,339.34) | 15,674.20 (12,536.77, 19,939.87) | 0.09 (0.06, 0.12) |
|  | Middle SDI | 13,742,886.37 (11,002,550.87, 17,443,054.90) | 14,816.22 (11,847.07, 18,827.77) | 38,849,052.83 (30,814,704.50, 49,658,083.44) | 15,438.01 (12,236.89, 19,742.48) | 0.16 (0.13, 0.18) |
|  | Low-middle SDI | 7,970,089.53 (6,348,563.04, 10,183,020.98) | 15,245.38 (12,118.48, 19,503.31) | 19,665,474.68 (15,624,818.33, 25,041,956.77) | 15,176.97 (12,047.25, 19,339.12) | −0.03 (−0.03, −0.02) |
|  | Low SDI | 2,610,018.73 (2,078,053.58, 3,323,227.84) | 13,565.49 (10,781.75, 17,289.78) | 5,999,168.18 (4,756,079.86, 7,648,359.25) | 13,652.15 (10,811.10, 17,414.64) | 0.02 (0.01, 0.03) |
|  | Andean Latin America | 185,036.44 (148,838.39, 229,550.00) | 10,461.02 (8414.25, 12,976.77) | 584,461.79 (459,056.94, 739,881.07) | 11,223.06 (8815.68, 14,207.12) | 0.24 (0.17, 0.32) |
|  | Australasia | 262,049.65 (205,244.88, 333,421.83) | 12,962.69 (10,172.09, 16,454.55) | 564,907.79 (442,626.57, 718,921.41) | 12,960.38 (10,173.10, 16,453.45) | 0.00 (0.00, 0.00) |
|  | Caribbean | 313,305.86 (241,129.51, 404,108.32) | 14,068.88 (10,828.17, 18,146.75) | 688,156.26 (529,496.92, 887,870.72) | 14,068.88 (10,828.17, 18,146.75) | 0.00 (0.00, 0.00) |
|  | Central Asia | 733,961.13 (571,985.40, 948,537.24) | 15,184.00 (11,825.15, 19,631.00) | 1,310,595.40 (1,022,481.44, 1,692,917.91) | 15,184.00 (11,825.15, 19,631.00) | 0.00 (0.00, 0.00) |
|  | Central Europe | 2,476,272.47 (1,933,394.14, 3,161,675.43) | 16,375.28 (12,787.94, 20,913.73) | 3,172,077.12 (2,475,180.95, 4,057,691.12) | 16,397.38 (12,842.03, 20,942.47) | 0.01 (0.00, 0.01) |
|  | Central Latin America | 1,066,895.66 (851,456.04, 1,348,203.58) | 14,668.98 (11,694.84, 18,546.52) | 3,462,210.23 (2,733,238.57, 4,406,189.35) | 14,773.56 (11,656.55, 18,809.67) | 0.03 (0.03, 0.04) |
|  | Central Sub-Saharan Africa | 285,854.02 (222,182.50, 363,621.92) | 13,068.96 (10,144.53, 16,678.80) | 688,924.24 (535,463.56, 876,773.92) | 13,068.96 (10,144.53, 16,678.80) | 0.00 (0.00, 0.00) |
|  | East Asia | 10,632,825.47 (8,466,042.79, 13,508,983.61) | 13,532.81 (10,760.53, 17,207.73) | 30,301,448.74 (23,998,001.67, 38,710,335.35) | 14,820.52 (11,735.75, 18,934.63) | 0.40 (0.33, 0.47) |
|  | Eastern Europe | 5,301,631.80 (4,240,462.00, 6,649,258.64) | 16,984.21 (13,595.05, 21,286.96) | 6,263,196.30 (5,006,079.86, 7,855,293.93) | 17,011.48 (13,624.26, 21,313.15) | 0.03 (0.00, 0.06) |
|  | Eastern Sub-Saharan Africa | 656,059.13 (519,737.35, 828,274.73) | 10,273.64 (8128.62, 12,971.44) | 1,552,087.20 (1,225,516.84, 1,946,280.11) | 10,449.26 (8242.25, 13,111.74) | 0.05 (0.03, 0.07) |
|  | High-income Asia Pacific | 2,108,970.12 (1,683,662.89, 2,644,887.96) | 10,811.20 (8632.88, 13,555.06) | 3,750,384.61 (2,946,566.25, 4,814,745.96) | 11,401.73 (8994.31, 14,585.62) | 0.22 (0.18, 0.26) |
|  | High-income North America | 5,351,541.58 (4,194,520.67, 6,958,018.47) | 17,592.38 (13,843.97, 22,831.70) | 9,996,212.80 (7,855,543.44, 13,000,234.36) | 17,427.82 (13,737.84, 22,618.19) | 0.01 (−0.06, 0.09) |
|  | North Africa and Middle East | 2,276,928.22 (1,794,072.51, 2,922,384.26) | 15,500.65 (12,193.47, 19,916.00) | 6,203,573.14 (4,947,410.23, 7,818,395.48) | 15,699.81 (12,497.78, 19,811.02) | 0.01 (0.00, 0.02) |
|  | Oceania | 38,247.85 (29,533.46, 49,793.47) | 15,346.47 (11,831.65, 20,020.04) | 97,454.71 (75,231.28, 126,903.50) | 15,346.47 (11,831.65, 20,020.04) | 0.00 (0.00, 0.00) |
|  | South Asia | 7,336,791.73 (5,813,861.34, 9,405,512.12) | 15,279.57 (12,087.18, 19,618.38) | 19,921,826.81 (15,777,239.88, 25,522,935.93) | 15,268.77 (12,081.07, 19,573.09) | −0.01 (−0.02, 0.00) |
|  | Southeast Asia | 4,149,520.87 (3,290,237.22, 5,288,475.17) | 17,513.18 (13,853.16, 22,362.78) | 11,003,032.38 (8,637,420.30, 14,098,312.56) | 17,227.07 (13,496.83, 22,119.15) | −0.06 (−0.08, −0.04) |
|  | Southern Latin America | 510,905.51 (404,908.19, 643,604.31) | 11,724.94 (9293.95, 14,761.40) | 946,370.92 (739,907.05, 1,215,047.96) | 12,103.45 (9473.28, 15,531.08) | 0.13 (0.12, 0.15) |
|  | Southern Sub-Saharan Africa | 354,895.90 (283,334.98, 455,277.92) | 13,779.81 (10,992.85, 17,683.94) | 811,818.71 (648,638.27, 1,041,980.91) | 13,805.56 (11,016.99, 17,729.56) | 0.01 (0.01, 0.01) |
|  | Tropical Latin America | 1,338,504.07 (1,065,752.45, 1,689,709.74) | 16,026.70 (12,742.89, 20,255.62) | 3,940,508.70 (3,120,529.37, 5,078,160.02) | 16,131.03 (12,771.83, 20,790.37) | −0.07 (−0.11, −0.02) |
|  | Western Europe | 8,825,060.96 (6,987,945.11, 11,279,548.36) | 17,101.26 (13,588.07, 21,804.32) | 12,640,676.89 (10,030,615.38, 16,215,990.06) | 17,400.12 (13,862.76, 22,257.92) | 0.09 (0.07, 0.12) |
|  | Western Sub-Saharan Africa | 1,083,803.42 (850,870.95, 1,401,171.60) | 15,189.46 (11,921.21, 19,641.30) | 2,775,078.95 (2,186,506.47, 3,576,150.01) | 15,213.10 (11,967.33, 19,632.42) | 0.00 (0.00, 0.01) |
| Incidence | | | | | | |
|  | Global | 1,600,595.00 (968,507.20, 2,419,775.63) | 440.67 (266.85, 666.18) | 3,603,661.16 (2,181,077.25, 5,394,488.44) | 460.29 (278.61, 689.26) | 0.17 (0.15, 0.18) |
|  | High SDI | 374,226.97 (222,425.71, 572,119.66) | 377.97 (223.73, 579.08) | 657,886.14 (393,533.43, 1,002,012.46) | 390.75 (232.91, 596.71) | 0.16 (0.13, 0.19) |
|  | High-middle SDI | 441,073.55 (268,039.26, 663,725.68) | 452.27 (275.07, 680.65) | 885,990.49 (538,976.98, 1,315,911.89) | 480.36 (292.07, 714.03) | 0.23 (0.21, 0.26) |
|  | Middle SDI | 454,171.27 (280,729.08, 681,164.59) | 487.00 (301.55, 729.77) | 1,262,818.61 (771,387.12, 1,879,363.78) | 498.50 (305.03, 741.84) | 0.11 (0.08, 0.13) |
|  | Low-middle SDI | 246,594.43 (147,376.02, 373,891.59) | 468.37 (280.94, 709.08) | 604,180.14 (359,957.52, 916,222.03) | 464.93 (277.69, 704.55) | −0.03 (−0.03, −0.02) |
|  | Low SDI | 82,652.70 (48,203.40, 127,013.18) | 428.03 (250.93, 656.64) | 189,787.99 (110,491.72, 290,993.49) | 429.84 (251.34, 658.38) | 0.02 (0.01, 0.03) |
|  | Andean Latin America | 6028.14 (3350.89, 9643.06) | 339.11 (188.84, 542.03) | 18,362.89 (10,155.14, 29,474.30) | 352.00 (194.82, 564.95) | 0.13 (0.09, 0.17) |
|  | Australasia | 7069.47 (3967.29, 11,297.46) | 356.46 (199.37, 571.58) | 15,354.42 (8624.79, 24,545.84) | 356.43 (199.37, 571.53) | 0.00 (0.00, 0.00) |
|  | Caribbean | 9081.78 (4956.26, 14,723.76) | 407.48 (222.23, 660.74) | 20,018.87 (10,920.30, 32,453.40) | 407.48 (222.23, 660.74) | 0.00 (0.00, 0.00) |
|  | Central Asia | 21,215.79 (11,619.45, 34,478.25) | 439.31 (240.51, 713.12) | 38,125.81 (20,789.59, 61,950.07) | 439.31 (240.51, 713.12) | 0.00 (0.00, 0.00) |
|  | Central Europe | 68,396.93 (39,131.84, 107,253.93) | 455.78 (261.33, 715.17) | 85,852.63 (49,526.70, 133,922.38) | 456.68 (263.20, 713.16) | 0.01 (0.01, 0.01) |
|  | Central Latin America | 30,787.14 (18,128.77, 47,037.94) | 421.47 (248.65, 643.49) | 99,253.37 (58,579.93, 153,754.98) | 423.36 (250.12, 655.45) | 0.02 (0.02, 0.02) |
|  | Central Sub-Saharan Africa | 9072.96 (4922.76, 14,662.59) | 415.64 (227.55, 671.82) | 22,026.71 (11,966.22, 35,605.37) | 415.64 (227.55, 671.82) | 0.00 (0.00, 0.00) |
|  | East Asia | 402,323.60 (250,923.56, 598,093.62) | 509.13 (318.08, 756.36) | 1,108,928.25 (682,437.61, 1,624,866.88) | 539.14 (332.17, 791.14) | 0.26 (0.21, 0.30) |
|  | Eastern Europe | 139,890.53 (85,418.14, 208,228.56) | 454.30 (277.35, 675.89) | 163,585.85 (99,935.54, 243,123.20) | 454.49 (277.46, 675.87) | 0.00 (0.00, 0.00) |
|  | Eastern Sub-Saharan Africa | 22,947.80 (13,315.17, 35,400.24) | 360.66 (210.36, 555.83) | 54,086.19 (31,397.21, 83,120.99) | 364.36 (212.60, 558.94) | 0.03 (0.02, 0.04) |
|  | High-income Asia Pacific | 54,830.01 (33,130.27, 83,293.00) | 281.64 (170.14, 427.94) | 98,312.04 (59,467.96, 149,526.41) | 302.13 (180.44, 461.78) | 0.32 (0.27, 0.38) |
|  | High-income North America | 121,148.51 (73,010.98, 180,792.04) | 406.96 (243.71, 608.62) | 230,125.37 (139,564.15, 345,328.67) | 409.38 (247.81, 615.08) | 0.06 (0.03, 0.09) |
|  | North Africa and Middle East | 64,033.35 (36,455.37, 101,452.42) | 435.54 (248.37, 690.02) | 173,863.41 (98,674.00, 275,249.93) | 438.14 (249.20, 693.08) | 0.01 (0.00, 0.02) |
|  | Oceania | 1270.80 (669.75, 2033.39) | 505.44 (269.49, 808.58) | 3279.30 (1723.12, 5249.02) | 505.44 (269.49, 808.58) | 0.00 (0.00, 0.00) |
|  | South Asia | 230,153.15 (140,300.21, 347,110.83) | 474.03 (289.95, 713.21) | 619,013.19 (375,488.12, 922,053.27) | 473.32 (287.68, 704.76) | 0.00 (0.00, 0.00) |
|  | Southeast Asia | 124,230.52 (72,324.70, 191,836.95) | 519.39 (303.28, 801.91) | 335,136.36 (195,712.26, 517,690.00) | 521.42 (305.18, 805.32) | 0.01 (0.01, 0.02) |
|  | Southern Latin America | 15,053.70 (8362.09, 24,432.09) | 348.16 (193.29, 565.20) | 27,106.45 (14,959.27, 44,145.05) | 348.59 (191.81, 568.53) | 0.00 (0.00, 0.00) |
|  | Southern Sub-Saharan Africa | 11,098.42 (6710.83, 16,761.53) | 432.47 (262.12, 652.69) | 25,391.44 (15,402.73, 38,301.89) | 433.08 (263.53, 652.71) | 0.00 (0.00, 0.01) |
|  | Tropical Latin America | 37,321.66 (22,868.62, 55,679.70) | 446.46 (274.11, 665.30) | 108,570.09 (66,741.39, 163,246.53) | 444.95 (273.69, 668.80) | −0.03 (−0.05, −0.02) |
|  | Western Europe | 192,223.22 (110,103.10, 303,964.13) | 374.98 (213.46, 594.65) | 273,385.18 (157,639.11, 431,525.92) | 373.96 (214.20, 592.51) | 0.01 (0.00, 0.03) |
|  | Western Sub-Saharan Africa | 32,417.53 (18,611.76, 49,867.36) | 454.92 (261.98, 699.82) | 83,883.36 (48,408.03, 129,881.92) | 455.77 (264.23, 705.37) | 0.00 (−0.01, 0.01) |
| DALYs | | | | | | |
|  | Global | 2,133,300.44 (535,491.93, 4,522,283.59) | 585.68 (147.03, 1241.93) | 4,562,189.11 (1,040,958.35, 9,836,077.78) | 582.08 (132.71, 1254.97) | 0.01 (0.00, 0.03) |
|  | High SDI | 605,858.02 (154,828.75, 1,291,649.99) | 605.75 (153.01, 1293.29) | 1,027,335.67 (253,264.80, 2,194,862.94) | 604.57 (146.51, 1292.99) | 0.02 (−0.01, 0.05) |
|  | High-middle SDI | 617,812.20 (184,056.40, 1,259,954.46) | 629.31 (186.97, 1284.55) | 1,136,435.09 (292,806.98, 2,394,768.16) | 613.60 (157.76, 1293.18) | −0.02 (−0.05, 0.00) |
|  | Middle SDI | 511,845.95 (101,840.72, 1,124,847.20) | 549.87 (110.16, 1208.47) | 1,437,917.05 (278,839.84, 3,199,664.22) | 570.33 (111.13, 1269.13) | 0.14 (0.12, 0.16) |
|  | Low-middle SDI | 295,806.52 (63,913.05, 642,103.07) | 563.77 (123.19, 1222.67) | 728,525.32 (157,305.23, 1,571,545.62) | 560.93 (121.82, 1209.67) | −0.02 (−0.02, −0.01) |
|  | Low SDI | 99,345.45 (25,255.53, 210,102.17) | 514.09 (132.01, 1086.56) | 227,838.80 (56,862.63, 486,872.42) | 516.72 (130.02, 1103.26) | 0.02 (0.02, 0.03) |
|  | Andean Latin America | 7424.33 (2167.44, 15,313.11) | 419.73 (123.10, 865.61) | 23,042.21 (6504.50, 48,196.85) | 442.52 (125.19, 925.63) | 0.18 (0.12, 0.25) |
|  | Australasia | 10,150.00 (2714.97, 21,337.66) | 502.20 (132.04, 1055.38) | 21,878.68 (5831.10, 46,742.43) | 502.85 (132.27, 1075.09) | −0.01 (−0.02, −0.01) |
|  | Caribbean | 11,969.23 (2763.26, 25,831.84) | 537.26 (123.88, 1159.42) | 26,063.25 (6038.66, 56,885.50) | 533.31 (123.90, 1163.58) | −0.02 (−0.02, −0.02) |
|  | Central Asia | 28,393.45 (7691.40, 61,067.53) | 586.41 (158.81, 1263.94) | 50,470.63 (13,473.47, 108,800.54) | 582.41 (155.89, 1258.21) | −0.01 (−0.01, −0.01) |
|  | Central Europe | 97,215.42 (29,481.16, 204,578.58) | 641.93 (193.99, 1351.74) | 123,930.81 (38,005.95, 259,878.79) | 643.19 (196.03, 1347.86) | 0.02 (0.02, 0.03) |
|  | Central Latin America | 40,655.22 (9493.05, 87,328.47) | 558.07 (131.39, 1197.64) | 131,401.32 (30,409.58, 281,854.72) | 560.41 (130.27, 1202.17) | 0.03 (0.02, 0.03) |
|  | Central Sub-Saharan Africa | 11,043.33 (2945.34, 23,212.18) | 502.05 (135.45, 1055.70) | 26,643.16 (7177.65, 55,520.51) | 503.49 (136.85, 1049.09) | 0.02 (0.02, 0.03) |
|  | East Asia | 392,742.71 (70,040.44, 872,293.48) | 497.83 (89.05, 1105.79) | 1,108,986.64 (181,817.66, 2,508,962.96) | 541.78 (89.06, 1225.61) | 0.36 (0.29, 0.42) |
|  | Eastern Europe | 237,721.40 (91,538.98, 447,556.57) | 761.02 (293.17, 1433.91) | 279,684.37 (108,895.51, 527,144.29) | 760.91 (295.40, 1435.11) | 0.06 (0.00, 0.13) |
|  | Eastern Sub-Saharan Africa | 26,639.73 (8420.56, 54,736.13) | 414.93 (131.81, 851.59) | 62,780.51 (19,487.13, 129,355.00) | 420.99 (131.24, 866.95) | 0.06 (0.05, 0.08) |
|  | High-income Asia Pacific | 84,008.04 (24,413.28, 176,967.73) | 430.52 (124.91, 907.12) | 147,047.44 (41,997.42, 311,672.71) | 448.96 (124.81, 950.09) | 0.17 (0.14, 0.20) |
|  | High-income North America | 201,021.82 (43,486.70, 447,261.70) | 662.22 (140.40, 1476.27) | 369,290.60 (81,146.51, 815,345.44) | 645.19 (140.08, 1426.14) | −0.04 (−0.10, 0.03) |
|  | North Africa and Middle East | 90,427.35 (22,834.58, 188,512.52) | 613.98 (157.04, 1282.12) | 242,601.54 (61,868.56, 508,548.12) | 612.58 (157.63, 1285.14) | −0.02 (−0.03, −0.01) |
|  | Oceania | 1405.08 (238.93, 3132.26) | 559.73 (96.27, 1251.20) | 3557.39 (605.97, 7967.87) | 556.57 (96.29, 1247.44) | −0.02 (−0.02, −0.01) |
|  | South Asia | 267,766.24 (57,933.70, 575,106.07) | 555.21 (121.66, 1191.90) | 727,345.81 (155,848.49, 1,581,271.24) | 556.15 (119.68, 1208.41) | 0.01 (0.00, 0.02) |
|  | Southeast Asia | 153,687.01 (24,957.95, 339,177.58) | 645.54 (105.84, 1429.01) | 407,134.23 (65,673.36, 908,360.03) | 634.78 (103.14, 1419.17) | −0.05 (−0.07, −0.03) |
|  | Southern Latin America | 20,173.20 (5642.19, 41,800.29) | 462.72 (129.01, 958.89) | 36,843.51 (10,029.77, 78,471.65) | 471.60 (127.45, 1004.56) | 0.09 (0.08, 0.11) |
|  | Southern Sub-Saharan Africa | 13,898.92 (3746.99, 29,343.71) | 538.45 (145.49, 1136.70) | 31,212.08 (8407.88, 65,828.93) | 529.38 (143.03, 1116.05) | −0.05 (−0.05, −0.04) |
|  | Tropical Latin America | 48,908.09 (10,217.18, 107,422.59) | 584.48 (123.05, 1283.56) | 143,314.24 (30,299.85, 314,483.77) | 586.55 (124.22, 1287.14) | −0.04 (−0.10, 0.02) |
|  | Western Europe | 346,701.08 (89,761.25, 730,334.85) | 673.10 (171.03, 1420.23) | 492,626.28 (125,762.37, 1,043,477.70) | 680.84 (170.36, 1445.35) | 0.08 (0.05, 0.11) |
|  | Western Sub-Saharan Africa | 41,348.82 (10,011.76, 88,450.64) | 578.09 (140.33, 1236.30) | 106,334.42 (25,419.46, 226,921.82) | 580.92 (140.83, 1239.71) | 0.03 (0.02, 0.03) |

DALYs: Disability-Adjusted Life Years; ASR: age-standardized rate; EAPC: estimated annual percentage change; CI: confidence interval; UI: uncertainty interval; SDI: socio-demographic index.

**Supplementary Fig. 1. The EAPC in the age-standardized rate (ASIR) of migraine among menopausal women across 5 SDI regions and 21 GBD regions.** (A) ASPR. (B) ASIR. (C) ASDR. ASPR: age-standardized prevalence rate; ASIR: age-standardized incidence rate; ASDR: age-standardized disability-adjusted life years rate; SDI: socio-demographic index; EAPC: estimated annual percentage change.

Supplementary Table 2. The prevalence of migraine in postmenopausal women in 204 countries and regions from 1990 to 2021.

| Location | 1990 | | 2023 | | EAPC (95% CI) |
| --- | --- | --- | --- | --- | --- |
|  | Number | ASR | Number | ASR |  |
| Afghanistan | 92,491.15 (71,995.86, 118,796.18) | 15,159.76 (11,776.35, 19,514.37) | 112,653.51 (87,615.13, 145,003.07) | 15,159.76 (11,776.35, 19,514.37) | 0.00 (0.00, 0.00) |
| Albania | 29,082.64 (22,463.15, 37,350.06) | 16,145.85 (12,464.62, 20,743.29) | 65,473.75 (50,538.17, 84,131.94) | 16,145.85 (12,464.62, 20,743.29) | 0.00 (0.00, 0.00) |
| Algeria | 170,387.64 (132,509.96, 219,022.36) | 15,159.76 (11,776.35, 19,514.37) | 473,671.55 (368,483.29, 608,928.44) | 15,159.76 (11,776.35, 19,514.37) | 0.00 (0.00, 0.00) |
| American Samoa | 291.81 (225.24, 380.05) | 15,346.47 (11,831.65, 20,020.04) | 676.87 (522.31, 881.88) | 15,346.47 (11,831.65, 20,020.04) | 0.00 (0.00, 0.00) |
| Andorra | 779.52 (611.41, 992.66) | 16,292.98 (12,787.16, 20,758.04) | 2066.69 (1621.91, 2635.19) | 16,292.98 (12,787.16, 20,758.04) | 0.00 (0.00, 0.00) |
| Angola | 43,895.94 (34,119.07, 55,845.65) | 13,068.96 (10,144.53, 16,678.80) | 151,659.85 (117,902.31, 192,950.74) | 13,068.96 (10,144.53, 16,678.80) | 0.00 (0.00, 0.00) |
| Antigua and Barbuda | 636.79 (489.48, 822.74) | 14,068.88 (10,828.17, 18,146.75) | 1438.03 (1107.42, 1853.87) | 14,068.88 (10,828.17, 18,146.75) | 0.00 (0.00, 0.00) |
| Argentina | 346,693.12 (273,876.79, 438,540.77) | 11,324.99 (8947.79, 14,318.72) | 574,444.44 (449,518.58, 735,212.39) | 11,647.78 (9124.71, 14,889.83) | 0.13 (0.11, 0.15) |
| Armenia | 43,896.41 (34,245.69, 56,715.69) | 15,184.00 (11,825.15, 19,631.00) | 68,197.35 (53,133.71, 88,132.96) | 15,184.00 (11,825.15, 19,631.00) | 0.00 (0.00, 0.00) |
| Australia | 215,484.06 (167,501.17, 275,812.40) | 12,797.09 (9958.24, 16,342.07) | 466,065.81 (362,268.59, 596,700.98) | 12,797.09 (9958.24, 16,342.07) | 0.00 (0.00, 0.00) |
| Austria | 161,748.28 (128,121.44, 206,042.40) | 15,167.54 (12,055.26, 19,292.16) | 222,527.40 (175,937.86, 284,336.34) | 15,146.11 (11,999.25, 19,326.28) | 0.17 (0.04, 0.30) |
| Azerbaijan | 80,301.24 (62,605.21, 103,822.63) | 15,184.00 (11,825.15, 19,631.00) | 174,536.11 (136,282.58, 225,400.92) | 15,184.00 (11,825.15, 19,631.00) | 0.00 (0.00, 0.00) |
| Bahrain | 2115.03 (1647.50, 2714.58) | 15,159.76 (11,776.35, 19,514.37) | 11,017.39 (8585.42, 14,138.42) | 15,159.76 (11,776.35, 19,514.37) | 0.00 (0.00, 0.00) |
| Bangladesh | 509,743.50 (392,828.01, 650,506.10) | 14,456.53 (11,134.40, 18,478.94) | 1,685,037.10 (1,298,316.33, 2,151,501.64) | 14,456.53 (11,134.40, 18,478.94) | 0.00 (0.00, 0.00) |
| Barbados | 3521.68 (2706.08, 4553.01) | 14,068.88 (10,828.17, 18,146.75) | 6887.13 (5300.35, 8883.83) | 14,068.88 (10,828.17, 18,146.75) | 0.00 (0.00, 0.00) |
| Belarus | 230,894.47 (180,654.23, 293,414.38) | 15,832.62 (12,391.50, 20,124.11) | 271,328.68 (212,087.50, 345,088.75) | 15,832.62 (12,391.50, 20,124.11) | 0.00 (0.00, 0.00) |
| Belgium | 275,172.29 (211,866.04, 363,330.55) | 19,706.45 (15,224.95, 25,960.95) | 424,707.91 (323,865.65, 540,606.33) | 22,949.07 (17,556.20, 29,072.62) | 0.53 (0.38, 0.68) |
| Belize | 1063.55 (818.39, 1372.32) | 14,068.88 (10,828.17, 18,146.75) | 3637.94 (2802.39, 4688.15) | 14,068.88 (10,828.17, 18,146.75) | 0.00 (0.00, 0.00) |
| Benin | 24,979.67 (19,307.68, 32,559.12) | 14,784.73 (11,420.71, 19,277.82) | 67,306.30 (52,045.65, 87,758.49) | 14,784.73 (11,420.71, 19,277.82) | 0.00 (0.00, 0.00) |
| Bermuda | 829.85 (638.65, 1070.59) | 14,068.88 (10,828.17, 18,146.75) | 1702.48 (1309.29, 2198.43) | 14,068.88 (10,828.17, 18,146.75) | 0.00 (0.00, 0.00) |
| Bhutan | 3036.77 (2341.23, 3872.75) | 14,456.53 (11,134.40, 18,478.94) | 7185.76 (5535.39, 9180.89) | 14,456.53 (11,134.40, 18,478.94) | 0.00 (0.00, 0.00) |
| Bolivia | 33,335.84 (25,586.56, 42,517.01) | 11,523.79 (8847.42, 14,708.53) | 96,471.68 (74,049.41, 123,077.66) | 11,523.79 (8847.42, 14,708.53) | 0.00 (0.00, 0.00) |
| Bosnia and Herzegovina | 70,669.48 (54,683.15, 90,659.95) | 16,145.85 (12,464.62, 20,743.29) | 94,458.56 (72,829.59, 121,464.77) | 16,145.85 (12,464.62, 20,743.29) | 0.00 (0.00, 0.00) |
| Botswana | 7059.58 (5483.43, 8992.26) | 13,068.96 (10,144.53, 16,678.80) | 18,996.16 (14,757.85, 24,193.71) | 13,068.96 (10,144.53, 16,678.80) | 0.00 (0.00, 0.00) |
| Brazil | 1,309,550.25 (1,042,750.25, 1,653,463.16) | 16,047.00 (12,759.36, 20,285.87) | 3,861,140.45 (3,056,099.49, 4,977,601.90) | 16,152.10 (12,782.18, 20,824.61) | −0.07 (−0.11, −0.03) |
| Brunei | 812.58 (639.32, 1033.78) | 10,272.44 (8074.34, 13,084.15) | 3401.49 (2678.41, 4326.79) | 10,272.44 (8074.34, 13,084.15) | 0.00 (0.00, 0.00) |
| Bulgaria | 199,698.39 (154,220.84, 256,514.32) | 16,145.85 (12,464.62, 20,743.29) | 200,433.05 (154,181.11, 258,059.14) | 16,145.85 (12,464.62, 20,743.29) | 0.00 (0.00, 0.00) |
| Burkina Faso | 57,683.05 (44,606.34, 75,179.06) | 14,784.73 (11,420.71, 19,277.82) | 122,023.56 (94,355.07, 159,094.57) | 14,784.73 (11,420.71, 19,277.82) | 0.00 (0.00, 0.00) |
| Burundi | 21,957.68 (16,893.07, 27,951.24) | 10,286.87 (7914.71, 13,099.45) | 41,418.89 (31,845.85, 52,734.43) | 10,286.87 (7914.71, 13,099.45) | 0.00 (0.00, 0.00) |
| Cambodia | 74,215.39 (57,052.59, 96,837.31) | 16,512.94 (12,664.57, 21,579.40) | 219,133.82 (168,382.62, 285,959.07) | 16,512.94 (12,664.57, 21,579.40) | 0.00 (0.00, 0.00) |
| Cameroon | 59,048.63 (45,696.35, 76,964.66) | 14,784.73 (11,420.71, 19,277.82) | 164,378.12 (127,184.97, 214,298.97) | 14,784.73 (11,420.71, 19,277.82) | 0.00 (0.00, 0.00) |
| Canada | 537,715.20 (433,928.03, 672,361.59) | 18,835.78 (15,216.44, 23,558.61) | 1,113,206.70 (855,222.26, 1,458,854.82) | 18,145.18 (13,973.44, 23,719.87) | −0.14 (−0.18, −0.10) |
| Cape Verde | 3288.86 (2539.90, 4291.05) | 14,784.73 (11,420.71, 19,277.82) | 6576.48 (5085.97, 8572.87) | 14,784.73 (11,420.71, 19,277.82) | 0.00 (0.00, 0.00) |
| Central African Republic | 14,330.05 (11,138.60, 18,227.58) | 13,068.96 (10,144.53, 16,678.80) | 27,987.42 (21,758.50, 35,596.36) | 13,068.96 (10,144.53, 16,678.80) | 0.00 (0.00, 0.00) |
| Chad | 35,889.87 (27,733.91, 46,798.24) | 14,784.73 (11,420.71, 19,277.82) | 65,292.84 (50,513.65, 85,138.53) | 14,784.73 (11,420.71, 19,277.82) | 0.00 (0.00, 0.00) |
| Chile | 119,570.86 (94,089.79, 152,265.39) | 12,825.30 (10,089.71, 16,335.97) | 313,670.01 (242,879.86, 406,677.86) | 12,995.66 (10,065.63, 16,847.35) | 0.06 (0.05, 0.07) |
| China | 10,178,715.40 (8,103,939.75, 12,942,323.94) | 13,473.56 (10,712.56, 17,145.28) | 29,199,265.16 (23,127,830.58, 37,280,176.00) | 14,803.76 (11,724.44, 18,903.42) | 0.41 (0.34, 0.48) |
| Colombia | 231,567.98 (180,934.55, 299,212.11) | 15,068.67 (11,756.72, 19,482.12) | 787,557.03 (614,269.90, 1,016,311.68) | 14,958.40 (11,666.49, 19,296.85) | −0.03 (−0.04, −0.03) |
| Comoros | 1779.57 (1369.29, 2265.06) | 10,286.87 (7914.71, 13,099.45) | 4672.49 (3594.38, 5948.92) | 10,286.87 (7914.71, 13,099.45) | 0.00 (0.00, 0.00) |
| Congo | 14,016.11 (10,889.54, 17,846.68) | 13,068.96 (10,144.53, 16,678.80) | 31,109.69 (24,178.79, 39,598.96) | 13,068.96 (10,144.53, 16,678.80) | 0.00 (0.00, 0.00) |
| Cook Islands | 158.77 (122.50, 206.96) | 15,346.47 (11,831.65, 20,020.04) | 367.39 (283.29, 479.39) | 15,346.47 (11,831.65, 20,020.04) | 0.00 (0.00, 0.00) |
| Costa Rica | 21,411.03 (16,746.52, 27,512.64) | 14,511.53 (11,344.71, 18,651.03) | 75,723.20 (59,224.55, 97,314.86) | 14,511.53 (11,344.71, 18,651.03) | 0.00 (0.00, 0.00) |
| Cote d’Ivoire | 47,055.02 (36,435.11, 61,340.51) | 14,784.73 (11,420.71, 19,277.82) | 134,584.21 (104,156.84, 175,464.15) | 14,784.73 (11,420.71, 19,277.82) | 0.00 (0.00, 0.00) |
| Croatia | 108,141.93 (83,552.42, 138,874.50) | 16,145.85 (12,464.62, 20,743.29) | 123,459.26 (96,297.35, 159,401.10) | 16,035.86 (12,551.64, 20,676.40) | −0.05 (−0.08, −0.01) |
| Cuba | 119,204.33 (91,713.87, 153,810.22) | 14,068.88 (10,828.17, 18,146.75) | 253,390.79 (194,885.42, 327,085.57) | 14,068.88 (10,828.17, 18,146.75) | 0.00 (0.00, 0.00) |
| Cyprus | 12,032.71 (9428.71, 15,330.75) | 16,292.98 (12,787.16, 20,758.04) | 28,363.70 (22,220.99, 36,170.87) | 16,292.98 (12,787.16, 20,758.04) | 0.00 (0.00, 0.00) |
| Czech Republic | 215,648.96 (166,297.74, 277,314.32) | 16,145.85 (12,464.62, 20,743.29) | 282,288.66 (217,072.18, 363,536.63) | 16,145.85 (12,464.62, 20,743.29) | 0.00 (0.00, 0.00) |
| Democratic Republic of the Congo | 203,835.86 (158,441.43, 259,244.87) | 13,068.96 (10,144.53, 16,678.80) | 459,106.59 (356,811.97, 584,357.46) | 13,068.96 (10,144.53, 16,678.80) | 0.00 (0.00, 0.00) |
| Denmark | 90,466.90 (71,804.92, 113,507.92) | 13,758.01 (10,941.06, 17,226.91) | 128,608.07 (101,539.61, 163,169.16) | 14,009.30 (11,095.33, 17,750.07) | 0.06 (0.05, 0.08) |
| Djibouti | 1151.83 (885.28, 1466.67) | 10,286.87 (7914.71, 13,099.45) | 5090.57 (3912.99, 6481.03) | 10,286.87 (7914.71, 13,099.45) | 0.00 (0.00, 0.00) |
| Dominica | 826.54 (635.70, 1066.92) | 14,068.88 (10,828.17, 18,146.75) | 1072.72 (825.50, 1384.01) | 14,068.88 (10,828.17, 18,146.75) | 0.00 (0.00, 0.00) |
| Dominican Republic | 44,930.19 (34,602.04, 57,921.07) | 14,068.88 (10,828.17, 18,146.75) | 122,602.65 (94,363.85, 158,138.81) | 14,068.88 (10,828.17, 18,146.75) | 0.00 (0.00, 0.00) |
| Ecuador | 58,722.35 (46,140.23, 76,413.55) | 13,125.59 (10,305.93, 17,088.12) | 189,223.82 (147,550.07, 245,947.28) | 12,992.94 (10,131.64, 16,890.14) | −0.05 (−0.06, −0.03) |
| Egypt | 371,716.25 (289,176.60, 481,784.20) | 15,541.19 (12,062.68, 20,174.57) | 841,585.53 (698,101.62, 1,015,341.48) | 14,722.43 (12,187.50, 17,761.83) | −0.24 (−0.29, −0.20) |
| El Salvador | 38,460.77 (30,083.28, 49,423.85) | 14,511.53 (11,344.71, 18,651.03) | 84,897.67 (66,328.28, 109,188.02) | 14,511.53 (11,344.71, 18,651.03) | 0.00 (0.00, 0.00) |
| Equatorial Guinea | 2434.36 (1891.64, 3099.40) | 13,068.96 (10,144.53, 16,678.80) | 6364.39 (4946.06, 8102.81) | 13,068.96 (10,144.53, 16,678.80) | 0.00 (0.00, 0.00) |
| Eritrea | 11,424.12 (8779.07, 14,546.54) | 10,286.87 (7914.71, 13,099.45) | 28,796.39 (22,140.06, 36,665.04) | 10,286.87 (7914.71, 13,099.45) | 0.00 (0.00, 0.00) |
| Estonia | 35,268.02 (27,580.26, 44,847.05) | 15,832.62 (12,391.50, 20,124.11) | 37,577.50 (29,312.07, 47,920.26) | 15,832.62 (12,391.50, 20,124.11) | 0.00 (0.00, 0.00) |
| Ethiopia | 153,888.69 (122,561.90, 193,983.36) | 9579.53 (7621.35, 12,084.18) | 342,431.07 (272,102.98, 430,376.09) | 9805.74 (7783.21, 12,328.77) | 0.10 (0.08, 0.12) |
| Federated States of Micronesia | 632.41 (487.86, 824.44) | 15,346.47 (11,831.65, 20,020.04) | 1135.10 (876.63, 1478.46) | 15,346.47 (11,831.65, 20,020.04) | 0.00 (0.00, 0.00) |
| Fiji | 4659.95 (3596.84, 6068.41) | 15,346.47 (11,831.65, 20,020.04) | 11,692.47 (9025.04, 15,232.24) | 15,346.47 (11,831.65, 20,020.04) | 0.00 (0.00, 0.00) |
| Finland | 106,063.54 (82,915.29, 135,408.53) | 16,292.98 (12,787.16, 20,758.04) | 155,710.91 (121,539.13, 199,095.57) | 16,292.98 (12,787.16, 20,758.04) | 0.00 (0.00, 0.00) |
| France | 1,177,497.70 (929,066.64, 1,517,149.48) | 15,992.86 (12,644.17, 20,570.55) | 1,727,104.99 (1,352,064.53, 2,221,855.11) | 15,984.67 (12,573.96, 20,486.96) | −0.06 (−0.13, 0.01) |
| Gabon | 7341.71 (5702.21, 9357.73) | 13,068.96 (10,144.53, 16,678.80) | 12,696.31 (9865.93, 16,167.59) | 13,068.96 (10,144.53, 16,678.80) | 0.00 (0.00, 0.00) |
| Georgia | 101,920.21 (79,400.69, 131,728.57) | 15,184.00 (11,825.15, 19,631.00) | 90,487.63 (70,410.11, 117,083.72) | 15,184.00 (11,825.15, 19,631.00) | −0.01 (−0.01, 0.00) |
| Germany | 2,108,094.70 (1,634,121.74, 2,731,355.09) | 18,290.57 (14,231.75, 23,618.58) | 2,895,940.93 (2,235,093.48, 3,778,983.26) | 18,878.92 (14,632.54, 24,574.40) | 0.11 (0.06, 0.16) |
| Ghana | 82,365.55 (63,723.29, 107,355.01) | 14,784.73 (11,420.71, 19,277.82) | 239,723.40 (185,442.35, 312,468.12) | 14,784.73 (11,420.71, 19,277.82) | 0.00 (0.00, 0.00) |
| Greece | 236,062.55 (185,287.49, 307,184.80) | 16,718.25 (13,135.70, 21,743.08) | 296,064.17 (229,743.95, 384,194.12) | 16,829.48 (13,113.04, 21,721.95) | 0.03 (0.02, 0.04) |
| Greenland | 532.94 (412.48, 694.03) | 17,659.20 (13,614.13, 23,087.55) | 1203.18 (932.38, 1565.49) | 17,659.20 (13,614.13, 23,087.55) | 0.00 (0.00, 0.00) |
| Grenada | 921.96 (708.87, 1191.59) | 14,068.88 (10,828.17, 18,146.75) | 1473.75 (1134.26, 1901.24) | 14,068.88 (10,828.17, 18,146.75) | 0.00 (0.00, 0.00) |
| Guam | 1024.31 (790.78, 1334.22) | 15,346.47 (11,831.65, 20,020.04) | 2984.83 (2300.23, 3893.97) | 15,346.47 (11,831.65, 20,020.04) | 0.00 (0.00, 0.00) |
| Guatemala | 44,334.05 (34,744.88, 56,904.50) | 14,511.53 (11,344.71, 18,651.03) | 144,770.34 (113,256.55, 185,965.71) | 14,511.53 (11,344.71, 18,651.03) | 0.00 (0.00, 0.00) |
| Guinea | 41,442.27 (32,033.47, 53,998.65) | 14,784.73 (11,420.71, 19,277.82) | 68,101.60 (52,666.57, 88,787.60) | 14,784.73 (11,420.71, 19,277.82) | 0.00 (0.00, 0.00) |
| Guinea-Bissau | 5064.69 (3918.60, 6600.58) | 14,784.73 (11,420.71, 19,277.82) | 9821.90 (7601.70, 12,803.22) | 14,784.73 (11,420.71, 19,277.82) | 0.00 (0.00, 0.00) |
| Guyana | 4626.23 (3563.06, 5961.82) | 14,068.88 (10,828.17, 18,146.75) | 8870.48 (6833.95, 11,427.58) | 14,068.88 (10,828.17, 18,146.75) | 0.00 (0.00, 0.00) |
| Haiti | 40,857.43 (31,479.63, 52,619.07) | 14,068.88 (10,828.17, 18,146.75) | 94,253.76 (72,637.46, 121,364.56) | 14,068.88 (10,828.17, 18,146.75) | 0.00 (0.00, 0.00) |
| Honduras | 25,688.02 (20,111.92, 32,991.33) | 14,511.53 (11,344.71, 18,651.03) | 84,320.29 (65,991.20, 108,279.69) | 14,511.53 (11,344.71, 18,651.03) | 0.00 (0.00, 0.00) |
| Hungary | 237,884.53 (183,531.15, 305,782.86) | 16,145.85 (12,464.62, 20,743.29) | 272,694.23 (209,723.41, 351,221.86) | 16,145.85 (12,464.62, 20,743.29) | 0.00 (0.00, 0.00) |
| Iceland | 3914.03 (3064.41, 4994.34) | 16,292.98 (12,787.16, 20,758.04) | 7730.81 (6053.70, 9862.34) | 16,292.98 (12,787.16, 20,758.04) | 0.00 (0.00, 0.00) |
| India | 6,093,375.92 (4,841,966.68, 7,823,784.77) | 15,414.70 (12,219.85, 19,829.92) | 16,457,936.10 (12,992,998.57, 21,125,597.74) | 15,419.57 (12,162.36, 19,803.55) | −0.01 (−0.02, 0.00) |
| Indonesia | 1,535,555.20 (1,204,181.16, 1,964,355.70) | 17,085.34 (13,366.72, 21,919.05) | 3,920,261.63 (3,074,322.43, 5,015,432.43) | 17,085.34 (13,366.72, 21,919.05) | 0.00 (0.00, 0.00) |
| Iran | 380,500.28 (303,497.60, 485,493.09) | 16,400.21 (13,042.68, 20,964.10) | 1,227,964.13 (971,376.00, 1,573,366.12) | 18,052.05 (14,254.01, 23,178.49) | 0.20 (0.11, 0.29) |
| Iraq | 100,121.78 (77,813.47, 128,813.96) | 15,159.76 (11,776.35, 19,514.37) | 317,670.97 (247,178.61, 408,194.44) | 15,159.76 (11,776.35, 19,514.37) | 0.00 (0.00, 0.00) |
| Ireland | 56,111.65 (43,882.74, 71,603.37) | 16,292.98 (12,787.16, 20,758.04) | 105,519.30 (82,640.73, 134,624.06) | 16,292.98 (12,787.16, 20,758.04) | 0.00 (0.00, 0.00) |
| Israel | 69,725.10 (54,598.95, 88,885.78) | 16,292.98 (12,787.16, 20,758.04) | 162,532.57 (127,125.40, 207,423.46) | 16,292.98 (12,787.16, 20,758.04) | 0.00 (0.00, 0.00) |
| Italy | 1,453,295.69 (1,149,719.16, 1,863,884.16) | 17,838.37 (14,157.16, 22,817.52) | 2,061,266.77 (1,619,541.46, 2,679,520.25) | 18,650.47 (14,722.56, 24,154.24) | 0.43 (0.34, 0.52) |
| Jamaica | 21,231.00 (16,327.44, 27,411.86) | 14,068.88 (10,828.17, 18,146.75) | 37,799.42 (29,090.24, 48,780.39) | 14,068.88 (10,828.17, 18,146.75) | 0.00 (0.00, 0.00) |
| Japan | 1,724,614.15 (1,369,553.35, 2,179,571.68) | 10,613.15 (8431.07, 13,408.76) | 2,648,256.36 (2,063,814.39, 3,409,963.65) | 11,313.17 (8881.04, 14,490.15) | 0.29 (0.24, 0.35) |
| Jordan | 16,901.66 (13,157.19, 21,713.70) | 15,159.76 (11,776.35, 19,514.37) | 96,963.87 (75,489.99, 124,550.51) | 15,159.76 (11,776.35, 19,514.37) | 0.00 (0.00, 0.00) |
| Kazakhstan | 198,981.73 (155,042.99, 257,066.14) | 15,184.00 (11,825.15, 19,631.00) | 293,474.70 (228,814.99, 379,168.41) | 15,184.00 (11,825.15, 19,631.00) | 0.00 (0.00, 0.00) |
| Kenya | 80,109.66 (63,259.08, 101,073.61) | 11,270.26 (8889.32, 14,222.20) | 234,353.61 (185,009.61, 295,688.05) | 11,270.26 (8889.32, 14,222.20) | 0.00 (0.00, 0.00) |
| Kiribati | 532.52 (410.98, 693.52) | 15,346.47 (11,831.65, 20,020.04) | 1170.98 (904.14, 1524.98) | 15,346.47 (11,831.65, 20,020.04) | 0.00 (0.00, 0.00) |
| Kuwait | 5363.58 (4210.23, 6898.97) | 14,910.74 (11,687.72, 19,204.75) | 31,687.62 (24,880.51, 40,713.02) | 14,764.48 (11,551.77, 18,993.33) | −0.05 (−0.05, −0.04) |
| Kyrgyzstan | 47,821.74 (37,265.04, 61,798.57) | 15,184.00 (11,825.15, 19,631.00) | 77,584.72 (60,541.09, 100,219.90) | 15,184.00 (11,825.15, 19,631.00) | 0.00 (0.00, 0.00) |
| Laos | 31,268.29 (24,030.45, 40,796.86) | 16,512.94 (12,664.57, 21,579.40) | 69,886.16 (53,739.34, 91,183.40) | 16,512.94 (12,664.57, 21,579.40) | 0.00 (0.00, 0.00) |
| Latvia | 61,810.77 (48,334.63, 78,591.63) | 15,832.62 (12,391.50, 20,124.11) | 58,368.22 (45,555.62, 74,415.84) | 15,832.62 (12,391.50, 20,124.11) | 0.00 (0.00, 0.00) |
| Lebanon | 30,350.52 (23,611.23, 39,005.94) | 15,159.76 (11,776.35, 19,514.37) | 79,410.14 (61,648.55, 102,310.46) | 15,159.76 (11,776.35, 19,514.37) | 0.00 (0.00, 0.00) |
| Lesotho | 11,864.18 (9211.89, 15,132.79) | 13,068.96 (10,144.53, 16,678.80) | 15,287.08 (11,871.82, 19,485.79) | 13,068.96 (10,144.53, 16,678.80) | 0.00 (0.00, 0.00) |
| Liberia | 12,946.31 (10,006.39, 16,867.57) | 14,784.73 (11,420.71, 19,277.82) | 25,202.56 (19,498.65, 32,873.64) | 14,784.73 (11,420.71, 19,277.82) | 0.00 (0.00, 0.00) |
| Libya | 23,042.03 (17,921.13, 29,616.66) | 15,159.76 (11,776.35, 19,514.37) | 67,006.36 (52,156.43, 86,076.20) | 15,159.76 (11,776.35, 19,514.37) | 0.00 (0.00, 0.00) |
| Lithuania | 73,255.56 (56,991.61, 93,509.72) | 15,139.60 (11,776.65, 19,320.47) | 82,442.75 (63,984.70, 105,490.95) | 15,139.60 (11,776.65, 19,320.47) | −0.04 (−0.06, −0.02) |
| Luxembourg | 7872.18 (6247.99, 10,102.84) | 15,507.69 (12,322.73, 19,870.71) | 13,573.20 (10,773.72, 17,416.65) | 15,507.69 (12,322.73, 19,870.71) | −0.06 (−0.15, 0.02) |
| Macedonia | 29,287.69 (22,648.50, 37,586.22) | 16,145.85 (12,464.62, 20,743.29) | 49,855.38 (38,468.56, 64,055.40) | 16,145.85 (12,464.62, 20,743.29) | 0.00 (0.00, 0.00) |
| Madagascar | 45,264.51 (34,811.96, 57,630.14) | 10,286.87 (7914.71, 13,099.45) | 105,916.26 (81,402.92, 134,865.45) | 10,286.87 (7914.71, 13,099.45) | 0.00 (0.00, 0.00) |
| Malawi | 36,226.82 (27,889.47, 46,100.74) | 10,286.87 (7914.71, 13,099.45) | 69,226.61 (53,287.30, 88,108.15) | 10,286.87 (7914.71, 13,099.45) | 0.00 (0.00, 0.00) |
| Malaysia | 110,032.29 (86,289.45, 139,755.34) | 13,923.68 (10,917.12, 17,702.84) | 362,552.74 (282,842.58, 465,290.88) | 14,131.14 (11,018.01, 18,152.67) | 0.07 (0.06, 0.08) |
| Maldives | 1141.09 (879.35, 1486.55) | 16,512.94 (12,664.57, 21,579.40) | 4371.12 (3362.18, 5703.28) | 16,512.94 (12,664.57, 21,579.40) | 0.00 (0.00, 0.00) |
| Mali | 51,815.88 (40,095.36, 67,530.09) | 14,784.73 (11,420.71, 19,277.82) | 109,621.86 (84,817.34, 142,891.38) | 14,784.73 (11,420.71, 19,277.82) | 0.00 (0.00, 0.00) |
| Malta | 6481.91 (5082.65, 8260.29) | 16,292.98 (12,787.16, 20,758.04) | 12,083.39 (9432.50, 15,438.20) | 16,292.98 (12,787.16, 20,758.04) | 0.00 (0.00, 0.00) |
| Marshall Islands | 205.72 (158.72, 268.05) | 15,346.47 (11,831.65, 20,020.04) | 492.39 (380.30, 640.76) | 15,346.47 (11,831.65, 20,020.04) | 0.00 (0.00, 0.00) |
| Mauritania | 13,042.43 (10,073.52, 17,013.16) | 14,784.73 (11,420.71, 19,277.82) | 27,653.19 (21,386.58, 36,046.38) | 14,784.73 (11,420.71, 19,277.82) | 0.00 (0.00, 0.00) |
| Mauritius | 11,194.07 (8589.12, 14,617.63) | 16,512.94 (12,664.57, 21,579.40) | 30,495.44 (23,394.76, 39,831.27) | 16,512.94 (12,664.57, 21,579.40) | 0.00 (0.00, 0.00) |
| Mexico | 545,807.79 (434,839.29, 688,199.37) | 14,583.78 (11,604.50, 18,403.45) | 1,746,197.20 (1,383,718.84, 2,225,571.26) | 14,811.77 (11,725.19, 18,899.34) | 0.07 (0.06, 0.09) |
| Moldova | 75,002.03 (58,712.21, 95,212.00) | 15,832.62 (12,391.50, 20,124.11) | 97,528.86 (76,247.65, 123,989.34) | 15,832.62 (12,391.50, 20,124.11) | 0.00 (0.00, 0.00) |
| Monaco | 885.64 (690.71, 1132.92) | 16,292.98 (12,787.16, 20,758.04) | 1202.85 (939.84, 1537.70) | 16,292.98 (12,787.16, 20,758.04) | 0.00 (0.00, 0.00) |
| Mongolia | 14,642.55 (11,404.71, 18,927.08) | 15,184.00 (11,825.15, 19,631.00) | 37,515.70 (29,284.75, 48,457.14) | 15,184.00 (11,825.15, 19,631.00) | 0.00 (0.00, 0.00) |
| Montenegro | 9949.87 (7689.78, 12,776.47) | 16,145.85 (12,464.62, 20,743.29) | 15,101.37 (11,646.98, 19,412.81) | 16,145.85 (12,464.62, 20,743.29) | 0.00 (0.00, 0.00) |
| Morocco | 183,198.15 (142,466.46, 235,532.83) | 15,159.76 (11,776.35, 19,514.37) | 477,052.37 (371,135.98, 613,112.62) | 15,159.76 (11,776.35, 19,514.37) | 0.00 (0.00, 0.00) |
| Mozambique | 54,629.56 (42,027.21, 69,545.54) | 10,286.87 (7914.71, 13,099.45) | 106,781.00 (82,135.77, 135,955.64) | 10,286.87 (7914.71, 13,099.45) | 0.00 (0.00, 0.00) |
| Myanmar | 362,523.83 (278,596.14, 473,025.25) | 16,512.94 (12,664.57, 21,579.40) | 826,093.68 (634,597.78, 1,078,097.05) | 16,512.94 (12,664.57, 21,579.40) | 0.00 (0.00, 0.00) |
| Namibia | 8144.10 (6324.01, 10,375.62) | 13,068.96 (10,144.53, 16,678.80) | 18,004.30 (13,984.97, 22,944.23) | 13,068.96 (10,144.53, 16,678.80) | 0.00 (0.00, 0.00) |
| Nauru | 51.83 (40.03, 67.46) | 15,346.47 (11,831.65, 20,020.04) | 84.15 (64.96, 109.59) | 15,346.47 (11,831.65, 20,020.04) | 0.00 (0.00, 0.00) |
| Nepal | 122,794.66 (94,786.31, 158,249.61) | 15,501.36 (11,931.82, 20,019.32) | 336,446.53 (260,060.86, 436,674.27) | 15,657.04 (12,088.89, 20,336.39) | 0.05 (0.04, 0.05) |
| Netherlands | 262,312.06 (205,908.25, 337,146.22) | 15,103.32 (11,906.95, 19,348.75) | 446,712.44 (347,480.28, 581,133.76) | 15,781.28 (12,303.36, 20,499.95) | 0.24 (0.19, 0.30) |
| New Zealand | 46,565.59 (36,506.20, 59,336.44) | 13,789.64 (10,837.25, 17,546.88) | 98,841.98 (77,574.03, 125,913.51) | 13,789.64 (10,837.25, 17,546.88) | −0.01 (−0.01, 0.00) |
| Nicaragua | 19,580.69 (15,327.09, 25,156.73) | 14,511.53 (11,344.71, 18,651.03) | 66,689.86 (52,198.84, 85,676.71) | 14,511.53 (11,344.71, 18,651.03) | 0.00 (0.00, 0.00) |
| Niger | 31,874.01 (24,666.07, 41,526.61) | 14,784.73 (11,420.71, 19,277.82) | 112,161.23 (86,793.06, 146,222.20) | 14,784.73 (11,420.71, 19,277.82) | 0.00 (0.00, 0.00) |
| Nigeria | 533,299.80 (417,516.96, 692,518.81) | 15,630.13 (12,248.50, 20,295.35) | 1,405,045.51 (1,114,184.95, 1,803,498.88) | 15,666.18 (12,395.67, 20,153.77) | 0.01 (0.00, 0.01) |
| Niue | 29.55 (22.74, 38.64) | 15,346.47 (11,831.65, 20,020.04) | 31.79 (24.51, 41.48) | 15,346.47 (11,831.65, 20,020.04) | 0.00 (0.00, 0.00) |
| North Korea | 247,014.58 (190,915.91, 320,420.10) | 14,289.52 (11,029.19, 18,569.51) | 465,947.36 (359,725.41, 605,542.41) | 14,289.52 (11,029.19, 18,569.51) | 0.00 (0.00, 0.00) |
| Northern Mariana Islands | 158.35 (122.23, 206.21) | 15,346.47 (11,831.65, 20,020.04) | 827.92 (639.08, 1077.55) | 15,346.47 (11,831.65, 20,020.04) | 0.00 (0.00, 0.00) |
| Norway | 86,256.35 (68,060.40, 111,133.58) | 16,177.35 (12,852.40, 20,764.58) | 125,324.42 (99,157.32, 160,872.97) | 16,289.31 (12,947.98, 20,846.80) | 0.00 (−0.02, 0.01) |
| Oman | 7115.94 (5530.38, 9151.38) | 15,159.76 (11,776.35, 19,514.37) | 21,867.28 (17,017.02, 28,092.08) | 15,159.76 (11,776.35, 19,514.37) | 0.00 (0.00, 0.00) |
| Pakistan | 607,840.89 (483,309.45, 782,462.42) | 14,678.73 (11,656.32, 18,897.00) | 1,435,221.31 (1,138,911.68, 1,848,477.44) | 14,512.13 (11,489.52, 18,692.20) | −0.05 (−0.06, −0.04) |
| Palau | 127.52 (98.41, 166.21) | 15,346.47 (11,831.65, 20,020.04) | 346.03 (267.21, 450.45) | 15,346.47 (11,831.65, 20,020.04) | 0.00 (0.00, 0.00) |
| Palestine | 12,609.11 (9807.45, 16,207.91) | 15,159.76 (11,776.35, 19,514.37) | 35,103.24 (27,323.29, 45,094.48) | 15,159.76 (11,776.35, 19,514.37) | 0.00 (0.00, 0.00) |
| Panama | 17,480.67 (13,667.78, 22,463.41) | 14,511.53 (11,344.71, 18,651.03) | 55,017.80 (42,998.71, 70,742.73) | 14,511.53 (11,344.71, 18,651.03) | 0.00 (0.00, 0.00) |
| Papua New Guinea | 23,485.47 (18,138.29, 30,567.87) | 15,346.47 (11,831.65, 20,020.04) | 63,249.53 (48,833.64, 82,340.49) | 15,346.47 (11,831.65, 20,020.04) | 0.00 (0.00, 0.00) |
| Paraguay | 28,953.83 (22,496.25, 37,532.94) | 15,167.10 (11,781.03, 19,663.70) | 79,368.25 (61,679.49, 102,862.27) | 15,167.10 (11,781.03, 19,663.70) | 0.00 (0.00, 0.00) |
| Peru | 92,978.24 (75,254.84, 113,970.11) | 9006.16 (7288.44, 11,034.17) | 298,766.30 (234,160.21, 378,550.51) | 10,250.54 (8038.00, 12,985.71) | 0.45 (0.32, 0.58) |
| Philippines | 446,839.31 (350,221.48, 572,101.67) | 17,085.34 (13,366.72, 21,919.05) | 1,333,131.66 (1,044,430.83, 1,707,435.42) | 17,085.34 (13,366.72, 21,919.05) | 0.00 (0.00, 0.00) |
| Poland | 753,724.95 (599,169.84, 959,620.30) | 16,907.64 (13,454.88, 21,520.51) | 1,084,287.80 (858,573.40, 1,384,493.55) | 16,907.64 (13,454.88, 21,520.51) | 0.00 (0.00, 0.00) |
| Portugal | 216,836.46 (169,887.08, 276,430.24) | 16,292.98 (12,787.16, 20,758.04) | 315,915.17 (246,859.06, 403,855.47) | 16,292.98 (12,787.16, 20,758.04) | 0.00 (0.00, 0.00) |
| Puerto Rico | 45,186.95 (34,766.26, 58,298.64) | 14,068.88 (10,828.17, 18,146.75) | 83,583.65 (64,216.47, 108,022.05) | 14,068.88 (10,828.17, 18,146.75) | 0.00 (0.00, 0.00) |
| Qatar | 841.84 (656.08, 1080.46) | 15,159.76 (11,776.35, 19,514.37) | 8255.15 (6439.43, 10,581.59) | 15,159.76 (11,776.35, 19,514.37) | 0.00 (0.00, 0.00) |
| Romania | 455,028.57 (351,536.11, 584,350.21) | 16,145.85 (12,464.62, 20,743.29) | 502,799.25 (386,756.24, 647,584.79) | 16,145.85 (12,464.62, 20,743.29) | 0.00 (0.00, 0.00) |
| Russian Federation | 3,531,299.95 (2,818,314.12, 4,454,827.90) | 17,327.35 (13,833.23, 21,843.45) | 4,385,936.77 (3,495,769.08, 5,535,941.23) | 17,327.35 (13,833.23, 21,843.45) | 0.03 (−0.01, 0.08) |
| Rwanda | 28,441.22 (21,883.54, 36,200.51) | 10,286.87 (7914.71, 13,099.45) | 66,128.40 (50,878.58, 84,175.41) | 10,286.87 (7914.71, 13,099.45) | 0.00 (0.00, 0.00) |
| Saint Kitts and Nevis | 477.02 (366.76, 615.95) | 14,068.88 (10,828.17, 18,146.75) | 1008.82 (777.62, 1299.08) | 14,068.88 (10,828.17, 18,146.75) | 0.00 (0.00, 0.00) |
| Saint Lucia | 1134.20 (872.77, 1462.95) | 14,068.88 (10,828.17, 18,146.75) | 3103.95 (2389.34, 4004.29) | 14,068.88 (10,828.17, 18,146.75) | 0.00 (0.00, 0.00) |
| Saint Vincent and the Grenadines | 919.79 (707.64, 1186.58) | 14,068.88 (10,828.17, 18,146.75) | 1755.09 (1351.29, 2263.34) | 14,068.88 (10,828.17, 18,146.75) | 0.00 (0.00, 0.00) |
| Samoa | 1151.28 (888.24, 1500.38) | 15,346.47 (11,831.65, 20,020.04) | 1970.50 (1520.22, 2568.71) | 15,346.47 (11,831.65, 20,020.04) | 0.00 (0.00, 0.00) |
| San Marino | 481.90 (377.09, 615.11) | 16,292.98 (12,787.16, 20,758.04) | 932.09 (729.79, 1190.77) | 16,292.98 (12,787.16, 20,758.04) | 0.00 (0.00, 0.00) |
| Sao Tome and Principe | 886.94 (685.53, 1156.24) | 14,784.73 (11,420.71, 19,277.82) | 1480.89 (1145.64, 1930.96) | 14,784.73 (11,420.71, 19,277.82) | 0.00 (0.00, 0.00) |
| Saudi Arabia | 53,403.06 (41,787.79, 67,928.42) | 14,018.93 (10,958.71, 17,840.99) | 191,501.93 (150,194.79, 243,424.95) | 14,018.93 (10,958.71, 17,840.99) | −0.02 (−0.03, −0.01) |
| Senegal | 39,491.27 (30,536.12, 51,477.95) | 14,784.73 (11,420.71, 19,277.82) | 103,535.73 (80,075.35, 134,983.33) | 14,784.73 (11,420.71, 19,277.82) | 0.00 (0.00, 0.00) |
| Serbia | 191,321.15 (147,664.12, 244,980.27) | 16,150.03 (12,452.49, 20,715.24) | 234,071.17 (180,034.29, 300,644.25) | 16,150.03 (12,452.49, 20,715.24) | 0.00 (0.00, 0.00) |
| Seychelles | 869.33 (665.91, 1137.10) | 16,512.94 (12,664.57, 21,579.40) | 1828.84 (1405.16, 2387.78) | 16,512.94 (12,664.57, 21,579.40) | 0.00 (0.00, 0.00) |
| Sierra Leone | 23,942.91 (18,501.42, 31,212.34) | 14,784.73 (11,420.71, 19,277.82) | 45,347.74 (35,060.64, 59,131.79) | 14,784.73 (11,420.71, 19,277.82) | 0.00 (0.00, 0.00) |
| Singapore | 16,344.08 (13,155.69, 20,120.34) | 8117.30 (6537.62, 9998.35) | 69,086.13 (55,385.73, 86,558.39) | 9060.46 (7261.76, 11,350.94) | 0.42 (0.31, 0.52) |
| Slovakia | 94,956.05 (73,278.40, 122,042.51) | 16,145.85 (12,464.62, 20,743.29) | 142,227.91 (109,562.53, 182,973.55) | 16,145.85 (12,464.62, 20,743.29) | 0.00 (0.00, 0.00) |
| Slovenia | 41,278.00 (31,867.21, 53,042.77) | 16,145.85 (12,464.62, 20,743.29) | 58,756.05 (45,237.01, 75,642.08) | 16,145.85 (12,464.62, 20,743.29) | 0.00 (0.00, 0.00) |
| Solomon Islands | 1664.86 (1285.74, 2166.90) | 15,346.47 (11,831.65, 20,020.04) | 4397.17 (3394.15, 5727.33) | 15,346.47 (11,831.65, 20,020.04) | 0.00 (0.00, 0.00) |
| Somalia | 21,392.23 (16,432.99, 27,246.49) | 10,286.87 (7914.71, 13,099.45) | 61,172.53 (47,085.32, 77,843.54) | 10,286.87 (7914.71, 13,099.45) | 0.00 (0.00, 0.00) |
| South Africa | 279,772.40 (223,462.69, 360,325.48) | 13,983.82 (11,160.17, 18,013.33) | 660,901.24 (528,040.03, 851,072.00) | 13,983.82 (11,160.17, 18,013.33) | 0.00 (0.00, 0.00) |
| South Korea | 367,199.31 (284,740.08, 467,751.61) | 12,051.74 (9332.79, 15,369.28) | 1,029,640.64 (801,624.76, 1,327,182.16) | 11,789.55 (9177.43, 15,192.29) | −0.15 (−0.19, −0.12) |
| South Sudan | 16,994.10 (13,081.70, 21,634.45) | 10,286.87 (7914.71, 13,099.45) | 32,278.65 (24,797.98, 41,111.84) | 10,286.87 (7914.71, 13,099.45) | 0.00 (0.00, 0.00) |
| Spain | 893,227.62 (699,317.53, 1,144,411.24) | 17,451.96 (13,686.19, 22,319.75) | 1,317,993.49 (1,026,943.92, 1,694,299.68) | 17,272.80 (13,507.94, 22,117.80) | 0.00 (−0.03, 0.03) |
| Sri Lanka | 153,023.18 (117,596.62, 199,705.83) | 16,512.94 (12,664.57, 21,579.40) | 444,085.73 (340,542.45, 580,208.09) | 16,512.94 (12,664.57, 21,579.40) | 0.00 (0.00, 0.00) |
| Sudan | 108,105.14 (84,016.97, 139,078.00) | 15,159.76 (11,776.35, 19,514.37) | 225,689.87 (175,650.87, 289,994.58) | 15,159.76 (11,776.35, 19,514.37) | 0.00 (0.00, 0.00) |
| Suriname | 3255.69 (2507.45, 4196.12) | 14,068.88 (10,828.17, 18,146.75) | 8611.43 (6630.09, 11,105.78) | 14,068.88 (10,828.17, 18,146.75) | 0.00 (0.00, 0.00) |
| Swaziland | 3383.90 (2628.00, 4313.53) | 13,068.96 (10,144.53, 16,678.80) | 7558.76 (5871.06, 9631.13) | 13,068.96 (10,144.53, 16,678.80) | 0.00 (0.00, 0.00) |
| Sweden | 209,301.17 (166,917.95, 267,463.84) | 18,008.43 (14,462.38, 22,868.99) | 274,930.29 (214,292.12, 353,474.33) | 17,646.37 (13,842.42, 22,564.87) | −0.10 (−0.11, −0.09) |
| Switzerland | 126,144.06 (99,429.12, 161,661.19) | 14,366.15 (11,353.99, 18,387.08) | 205,233.91 (160,751.34, 263,504.78) | 14,493.56 (11,382.38, 18,584.41) | 0.03 (0.01, 0.04) |
| Syria | 67,423.96 (52,488.18, 86,603.39) | 15,159.76 (11,776.35, 19,514.37) | 187,254.90 (145,769.27, 240,520.45) | 15,159.76 (11,776.35, 19,514.37) | 0.00 (0.00, 0.00) |
| Taiwan (Province of China) | 207,095.50 (164,548.91, 260,162.43) | 15,901.79 (12,614.36, 19,995.29) | 636,236.22 (487,018.53, 830,760.74) | 16,078.81 (12,313.26, 20,980.11) | 0.06 (0.05, 0.07) |
| Tajikistan | 41,118.42 (32,050.78, 53,139.42) | 15,184.00 (11,825.15, 19,631.00) | 89,056.34 (69,563.46, 114,975.14) | 15,184.00 (11,825.15, 19,631.00) | 0.00 (0.00, 0.00) |
| Tanzania | 99,457.87 (78,290.04, 125,376.42) | 10,397.54 (8176.22, 13,133.89) | 241,887.47 (188,374.09, 308,704.20) | 10,931.85 (8504.14, 13,955.25) | 0.12 (0.04, 0.20) |
| Thailand | 741,407.11 (589,184.38, 923,735.87) | 22,254.38 (17,630.58, 27,779.81) | 2,083,597.58 (1,594,816.91, 2,709,047.02) | 19,660.27 (15,043.06, 25,574.06) | −0.43 (−0.55, −0.31) |
| The Bahamas | 1943.32 (1495.39, 2506.94) | 14,068.88 (10,828.17, 18,146.75) | 5790.41 (4460.08, 7462.97) | 14,068.88 (10,828.17, 18,146.75) | 0.00 (0.00, 0.00) |
| The Gambia | 3837.99 (2966.28, 5003.79) | 14,784.73 (11,420.71, 19,277.82) | 11,922.98 (9216.29, 15,546.42) | 14,784.73 (11,420.71, 19,277.82) | 0.00 (0.00, 0.00) |
| Timor-Leste | 3861.43 (2974.10, 5031.81) | 16,512.94 (12,664.57, 21,579.40) | 11,886.77 (9109.25, 15,530.25) | 16,512.94 (12,664.57, 21,579.40) | 0.00 (0.00, 0.00) |
| Togo | 15,812.50 (12,231.65, 20,617.16) | 14,784.73 (11,420.71, 19,277.82) | 55,266.48 (42,754.80, 72,052.10) | 14,784.73 (11,420.71, 19,277.82) | 0.00 (0.00, 0.00) |
| Tokelau | 18.50 (14.28, 24.14) | 15,346.47 (11,831.65, 20,020.04) | 19.63 (15.13, 25.62) | 15,346.47 (11,831.65, 20,020.04) | 0.00 (0.00, 0.00) |
| Tonga | 773.40 (596.76, 1007.73) | 15,346.47 (11,831.65, 20,020.04) | 1085.97 (837.15, 1416.82) | 15,346.47 (11,831.65, 20,020.04) | 0.00 (0.00, 0.00) |
| Trinidad and Tobago | 10,046.63 (7730.52, 12,959.25) | 14,068.88 (10,828.17, 18,146.75) | 25,561.76 (19,676.05, 32,959.55) | 14,068.88 (10,828.17, 18,146.75) | 0.00 (0.00, 0.00) |
| Tunisia | 66,979.58 (52,116.66, 86,062.28) | 15,159.76 (11,776.35, 19,514.37) | 185,827.51 (144,457.03, 238,994.28) | 15,159.76 (11,776.35, 19,514.37) | 0.00 (0.00, 0.00) |
| Turkey | 515,297.23 (399,863.16, 668,816.44) | 15,681.90 (12,145.67, 20,363.41) | 1,390,382.11 (1,080,944.69, 1,789,207.19) | 15,731.65 (12,225.84, 20,248.32) | 0.01 (0.00, 0.01) |
| Turkmenistan | 29,809.51 (23,239.58, 38,504.82) | 15,184.00 (11,825.15, 19,631.00) | 64,604.97 (50,411.09, 83,439.64) | 15,184.00 (11,825.15, 19,631.00) | 0.00 (0.00, 0.00) |
| Tuvalu | 103.82 (80.14, 135.18) | 15,346.47 (11,831.65, 20,020.04) | 154.67 (119.34, 201.57) | 15,346.47 (11,831.65, 20,020.04) | 0.00 (0.00, 0.00) |
| Uganda | 56,885.64 (43,765.70, 72,413.21) | 10,286.87 (7914.71, 13,099.45) | 143,322.86 (110,199.77, 182,507.88) | 10,286.87 (7914.71, 13,099.45) | 0.00 (0.00, 0.00) |
| Ukraine | 1,294,101.01 (1,027,417.90, 1,639,427.17) | 16,582.30 (13,201.06, 20,988.97) | 1,330,013.52 (1,055,703.36, 1,685,813.29) | 16,582.30 (13,201.06, 20,988.97) | 0.00 (0.00, 0.00) |
| United Arab Emirates | 3398.97 (2649.02, 4359.33) | 15,159.93 (11,796.36, 19,474.75) | 30,472.48 (23,876.41, 39,015.86) | 15,160.04 (11,806.71, 19,510.41) | 0.00 (0.00, 0.00) |
| United Kingdom | 1,257,043.86 (993,806.28, 1,607,881.70) | 16,509.85 (13,113.08, 21,045.03) | 1,697,501.80 (1,343,387.67, 2,172,780.23) | 16,600.44 (13,179.14, 21,201.74) | 0.02 (0.02, 0.03) |
| United States | 4,813,170.72 (3,740,875.34, 6,308,607.76) | 17,461.46 (13,633.25, 22,838.00) | 8,881,646.16 (6,988,467.30, 11,550,424.12) | 17,341.96 (13,690.01, 22,509.34) | 0.03 (−0.06, 0.11) |
| Uruguay | 44,617.27 (34,862.89, 56,921.70) | 12,266.03 (9594.10, 15,631.20) | 58,204.24 (45,433.61, 74,343.02) | 12,266.03 (9594.10, 15,631.20) | 0.00 (0.00, 0.00) |
| Uzbekistan | 175,469.30 (136,730.70, 226,834.32) | 15,184.00 (11,825.15, 19,631.00) | 415,137.88 (324,039.65, 536,040.09) | 15,184.00 (11,825.15, 19,631.00) | 0.00 (0.00, 0.00) |
| Vanuatu | 737.76 (569.60, 960.53) | 15,346.47 (11,831.65, 20,020.04) | 2391.88 (1846.26, 3114.61) | 15,346.47 (11,831.65, 20,020.04) | 0.00 (0.00, 0.00) |
| Venezuela | 122,564.66 (97,394.89, 153,518.70) | 14,532.56 (11,547.62, 18,203.87) | 417,036.84 (326,761.36, 533,922.31) | 14,600.67 (11,433.84, 18,699.61) | 0.02 (0.02, 0.03) |
| Vietnam | 671,587.68 (515,352.66, 877,169.63) | 16,512.94 (12,664.57, 21,579.40) | 1,680,360.27 (1,290,639.39, 2,193,273.22) | 16,512.94 (12,664.57, 21,579.40) | 0.00 (0.00, 0.00) |
| Virgin Islands, U.S. | 1083.81 (834.57, 1397.09) | 14,068.88 (10,828.17, 18,146.75) | 2324.03 (1787.10, 3000.69) | 14,068.88 (10,828.17, 18,146.75) | 0.00 (0.00, 0.00) |
| Yemen | 64,319.76 (50,020.48, 82,695.38) | 15,159.76 (11,776.35, 19,514.37) | 184,749.16 (143,756.16, 237,390.43) | 15,159.76 (11,776.35, 19,514.37) | 0.00 (0.00, 0.00) |
| Zambia | 25,986.75 (20,146.67, 33,084.55) | 11,255.16 (8720.81, 14,337.00) | 67,259.19 (52,501.99, 85,541.07) | 11,302.09 (8812.54, 14,379.33) | −0.02 (−0.04, 0.00) |
| Zimbabwe | 44,671.74 (34,688.31, 56,948.07) | 13,068.96 (10,144.53, 16,678.80) | 91,071.16 (70,741.97, 115,966.72) | 13,068.96 (10,144.53, 16,678.80) | 0.00 (0.00, 0.00) |

ASR: age-standardized rate; EAPC: estimated annual percentage change; CI: confidence interval.

Supplementary Table 3. The incidence of migraine in postmenopausal women in 204 countries and regions from 1990 to 2021.

| Location | 1990 | | 2023 | | EAPC (95% CI) |
| --- | --- | --- | --- | --- | --- |
|  | Number | ASR | Number | ASR |  |
| Afghanistan | 2628.21 (1460.40, 4274.90) | 430.01 (239.34, 698.54) | 3283.13 (1822.98, 5334.29) | 430.01 (239.34, 698.54) | 0.00 (0.00, 0.00) |
| Albania | 807.76 (438.55, 1316.83) | 447.98 (243.49, 730.25) | 1802.80 (980.35, 2937.78) | 447.98 (243.49, 730.25) | 0.00 (0.00, 0.00) |
| Algeria | 4847.01 (2696.70, 7880.20) | 430.01 (239.34, 698.54) | 13,511.93 (7510.97, 21,970.67) | 430.01 (239.34, 698.54) | 0.00 (0.00, 0.00) |
| American Samoa | 9.66 (5.11, 15.45) | 505.44 (269.49, 808.58) | 22.64 (11.95, 36.24) | 505.44 (269.49, 808.58) | 0.00 (0.00, 0.00) |
| Andorra | 17.33 (9.41, 28.19) | 368.35 (200.35, 598.41) | 47.34 (25.80, 76.79) | 368.35 (200.35, 598.41) | 0.00 (0.00, 0.00) |
| Angola | 1396.66 (758.19, 2257.60) | 415.64 (227.55, 671.82) | 4866.67 (2641.35, 7867.17) | 415.64 (227.55, 671.82) | 0.00 (0.00, 0.00) |
| Antigua and Barbuda | 18.35 (10.07, 29.73) | 407.48 (222.23, 660.74) | 41.82 (22.76, 67.86) | 407.48 (222.23, 660.74) | 0.00 (0.00, 0.00) |
| Argentina | 10,478.33 (5799.97, 17,120.08) | 345.80 (191.24, 565.10) | 16,882.67 (9299.90, 27,513.63) | 345.34 (189.65, 563.83) | −0.01 (−0.01, −0.01) |
| Armenia | 1277.58 (699.24, 2078.53) | 439.31 (240.51, 713.12) | 1937.55 (1060.18, 3149.87) | 439.31 (240.51, 713.12) | 0.00 (0.00, 0.00) |
| Australia | 5834.04 (3192.82, 9518.85) | 353.34 (192.61, 578.80) | 12,709.11 (6964.22, 20,733.30) | 353.34 (192.61, 578.80) | 0.00 (0.00, 0.00) |
| Austria | 3819.60 (2105.26, 6107.87) | 364.02 (199.79, 582.55) | 5419.82 (2993.06, 8660.12) | 364.02 (199.79, 582.55) | 0.01 (0.00, 0.03) |
| Azerbaijan | 2366.54 (1294.08, 3843.91) | 439.31 (240.51, 713.12) | 5112.81 (2785.22, 8312.71) | 439.31 (240.51, 713.12) | 0.00 (0.00, 0.00) |
| Bahrain | 60.46 (33.62, 98.40) | 430.01 (239.34, 698.54) | 313.49 (173.89, 510.28) | 430.01 (239.34, 698.54) | 0.00 (0.00, 0.00) |
| Bangladesh | 16,078.46 (8872.88, 25,808.56) | 451.03 (249.27, 723.82) | 52,907.21 (29,192.70, 84,883.00) | 451.03 (249.27, 723.82) | 0.00 (0.00, 0.00) |
| Barbados | 101.93 (56.10, 165.02) | 407.48 (222.23, 660.74) | 197.97 (108.13, 320.93) | 407.48 (222.23, 660.74) | 0.00 (0.00, 0.00) |
| Belarus | 6244.42 (3478.49, 10,025.69) | 430.98 (240.19, 691.93) | 7262.19 (4045.02, 11,660.72) | 430.98 (240.19, 691.93) | 0.00 (0.00, 0.00) |
| Belgium | 5128.20 (2745.37, 8458.44) | 368.78 (195.60, 610.44) | 6655.56 (3578.87, 11,119.87) | 360.26 (192.16, 604.30) | −0.08 (−0.10, −0.06) |
| Belize | 30.79 (16.81, 49.91) | 407.48 (222.23, 660.74) | 105.99 (57.54, 172.05) | 407.48 (222.23, 660.74) | 0.00 (0.00, 0.00) |
| Benin | 743.54 (396.99, 1206.82) | 440.97 (236.06, 715.48) | 2020.63 (1075.47, 3281.54) | 440.97 (236.06, 715.48) | 0.00 (0.00, 0.00) |
| Bermuda | 24.01 (13.11, 38.93) | 407.48 (222.23, 660.74) | 48.91 (26.79, 79.26) | 407.48 (222.23, 660.74) | 0.00 (0.00, 0.00) |
| Bhutan | 95.84 (52.74, 153.71) | 451.03 (249.27, 723.82) | 224.59 (124.03, 360.37) | 451.03 (249.27, 723.82) | 0.00 (0.00, 0.00) |
| Bolivia | 1031.59 (577.78, 1661.88) | 356.06 (199.57, 573.32) | 2973.42 (1664.77, 4788.59) | 356.06 (199.57, 573.32) | 0.00 (0.00, 0.00) |
| Bosnia and Herzegovina | 1979.10 (1069.45, 3225.15) | 447.98 (243.49, 730.25) | 2577.35 (1402.16, 4198.64) | 447.98 (243.49, 730.25) | 0.00 (0.00, 0.00) |
| Botswana | 222.62 (121.24, 359.61) | 415.64 (227.55, 671.82) | 603.04 (328.26, 974.60) | 415.64 (227.55, 671.82) | 0.00 (0.00, 0.00) |
| Brazil | 36,521.12 (22,429.42, 54,405.49) | 447.09 (275.13, 665.25) | 106,375.44 (65,539.72, 159,735.67) | 445.50 (274.64, 668.74) | −0.03 (−0.05, −0.02) |
| Brunei | 23.31 (12.64, 37.95) | 289.48 (157.57, 470.68) | 96.55 (52.21, 157.32) | 289.48 (157.57, 470.68) | 0.00 (0.00, 0.00) |
| Bulgaria | 5493.01 (2975.82, 8941.31) | 447.98 (243.49, 730.25) | 5440.84 (2981.66, 8863.26) | 447.98 (243.49, 730.25) | 0.00 (0.00, 0.00) |
| Burkina Faso | 1720.98 (915.99, 2794.13) | 440.97 (236.06, 715.48) | 3663.10 (1949.72, 5949.26) | 440.97 (236.06, 715.48) | 0.00 (0.00, 0.00) |
| Burundi | 772.54 (424.79, 1230.17) | 361.47 (199.31, 575.10) | 1451.98 (794.56, 2313.72) | 361.47 (199.31, 575.10) | 0.00 (0.00, 0.00) |
| Cambodia | 2295.05 (1227.72, 3712.45) | 505.38 (271.24, 816.86) | 6745.57 (3608.46, 10,912.57) | 505.38 (271.24, 816.86) | 0.00 (0.00, 0.00) |
| Cameroon | 1765.28 (937.02, 2868.85) | 440.97 (236.06, 715.48) | 4932.67 (2617.95, 8014.74) | 440.97 (236.06, 715.48) | 0.00 (0.00, 0.00) |
| Canada | 10,655.61 (5796.42, 17,372.21) | 380.20 (205.85, 620.30) | 23,173.76 (12,650.16, 38,527.42) | 383.74 (208.76, 639.37) | 0.05 (0.03, 0.07) |
| Cape Verde | 99.66 (53.50, 161.75) | 440.97 (236.06, 715.48) | 194.76 (103.80, 316.20) | 440.97 (236.06, 715.48) | 0.00 (0.00, 0.00) |
| Central African Republic | 456.97 (247.98, 738.59) | 415.64 (227.55, 671.82) | 894.32 (485.19, 1,445.92) | 415.64 (227.55, 671.82) | 0.00 (0.00, 0.00) |
| Chad | 1076.95 (574.71, 1748.02) | 440.97 (236.06, 715.48) | 1966.48 (1042.97, 3195.10) | 440.97 (236.06, 715.48) | 0.00 (0.00, 0.00) |
| Chile | 3314.26 (1839.07, 5380.62) | 354.95 (196.98, 576.01) | 8571.23 (4640.69, 14,148.24) | 354.68 (191.69, 585.63) | 0.00 (0.00, 0.00) |
| China | 386,356.88 (242,107.07, 573,477.08) | 508.65 (319.27, 754.41) | 1,071,719.56 (660,633.06, 1,567,682.10) | 539.95 (333.26, 791.07) | 0.27 (0.22, 0.31) |
| Colombia | 6404.92 (3489.40, 10,300.53) | 415.29 (226.77, 668.00) | 21,833.89 (11,837.44, 35,330.11) | 414.99 (225.10, 671.39) | 0.00 (0.00, 0.00) |
| Comoros | 62.04 (33.99, 98.83) | 361.47 (199.31, 575.10) | 164.16 (90.16, 261.39) | 361.47 (199.31, 575.10) | 0.00 (0.00, 0.00) |
| Congo | 443.48 (241.36, 716.65) | 415.64 (227.55, 671.82) | 995.72 (541.11, 1609.22) | 415.64 (227.55, 671.82) | 0.00 (0.00, 0.00) |
| Cook Islands | 5.31 (2.81, 8.50) | 505.44 (269.49, 808.58) | 12.18 (6.48, 19.49) | 505.44 (269.49, 808.58) | 0.00 (0.00, 0.00) |
| Costa Rica | 610.29 (334.25, 978.19) | 413.08 (226.25, 661.97) | 2150.18 (1176.96, 3445.76) | 413.08 (226.25, 661.97) | 0.00 (0.00, 0.00) |
| Cote d’Ivoire | 1412.56 (746.81, 2296.89) | 440.97 (236.06, 715.48) | 4044.68 (2143.85, 6574.17) | 440.97 (236.06, 715.48) | 0.00 (0.00, 0.00) |
| Croatia | 2975.88 (1608.65, 4844.99) | 447.98 (243.49, 730.25) | 3376.49 (1848.28, 5429.51) | 446.41 (242.65, 717.75) | −0.03 (−0.03, −0.02) |
| Cuba | 3459.07 (1890.73, 5606.58) | 407.48 (222.23, 660.74) | 7439.49 (4060.66, 12,055.74) | 407.48 (222.23, 660.74) | 0.00 (0.00, 0.00) |
| Cyprus | 266.21 (144.93, 433.76) | 368.35 (200.35, 598.41) | 639.23 (348.38, 1039.81) | 368.35 (200.35, 598.41) | 0.00 (0.00, 0.00) |
| Czech Republic | 5876.33 (3193.43, 9557.97) | 447.98 (243.49, 730.25) | 7680.03 (4208.87, 12,509.42) | 447.98 (243.49, 730.25) | 0.00 (0.00, 0.00) |
| Democratic Republic of the Congo | 6464.94 (3505.72, 10,447.24) | 415.64 (227.55, 671.82) | 14,659.84 (7966.74, 23,697.02) | 415.64 (227.55, 671.82) | 0.00 (0.00, 0.00) |
| Denmark | 2354.74 (1282.05, 3847.58) | 361.26 (195.22, 593.33) | 3306.16 (1828.48, 5314.55) | 359.96 (197.60, 579.49) | −0.01 (−0.02, −0.01) |
| Djibouti | 40.55 (22.15, 64.64) | 361.47 (199.31, 575.10) | 179.25 (98.02, 285.81) | 361.47 (199.31, 575.10) | 0.00 (0.00, 0.00) |
| Dominica | 23.96 (13.12, 38.82) | 407.48 (222.23, 660.74) | 31.23 (17.02, 50.63) | 407.48 (222.23, 660.74) | 0.00 (0.00, 0.00) |
| Dominican Republic | 1309.16 (712.34, 2124.46) | 407.48 (222.23, 660.74) | 3561.95 (1941.71, 5775.86) | 407.48 (222.23, 660.74) | 0.00 (0.00, 0.00) |
| Ecuador | 1659.55 (894.33, 2696.54) | 368.77 (198.99, 599.06) | 5378.14 (2907.83, 8711.47) | 367.97 (198.97, 596.10) | −0.01 (−0.01, 0.00) |
| Egypt | 10,283.51 (5669.33, 16,629.80) | 430.95 (238.80, 695.81) | 23,861.96 (12,914.39, 37,957.74) | 419.92 (229.27, 664.78) | −0.12 (−0.14, −0.09) |
| El Salvador | 1095.14 (599.15, 1754.50) | 413.08 (226.25, 661.97) | 2416.43 (1324.49, 3871.47) | 413.08 (226.25, 661.97) | 0.00 (0.00, 0.00) |
| Equatorial Guinea | 77.58 (42.20, 125.37) | 415.64 (227.55, 671.82) | 203.95 (110.88, 329.56) | 415.64 (227.55, 671.82) | 0.00 (0.00, 0.00) |
| Eritrea | 401.63 (218.89, 640.53) | 361.47 (199.31, 575.10) | 1011.88 (553.70, 1612.35) | 361.47 (199.31, 575.10) | 0.00 (0.00, 0.00) |
| Estonia | 954.19 (531.55, 1531.68) | 430.98 (240.19, 691.93) | 1004.90 (560.65, 1612.20) | 430.98 (240.19, 691.93) | 0.00 (0.00, 0.00) |
| Ethiopia | 5544.34 (3446.34, 8154.53) | 347.81 (217.62, 509.93) | 12,472.81 (7790.32, 18,237.78) | 356.77 (223.52, 521.57) | 0.11 (0.09, 0.13) |
| Federated States of Micronesia | 20.75 (11.08, 33.20) | 505.44 (269.49, 808.58) | 37.72 (19.87, 60.37) | 505.44 (269.49, 808.58) | 0.00 (0.00, 0.00) |
| Fiji | 156.50 (82.35, 250.52) | 505.44 (269.49, 808.58) | 389.37 (205.80, 623.12) | 505.44 (269.49, 808.58) | 0.00 (0.00, 0.00) |
| Finland | 2362.51 (1289.22, 3845.24) | 368.35 (200.35, 598.41) | 3478.32 (1903.24, 5655.37) | 368.35 (200.35, 598.41) | 0.00 (0.00, 0.00) |
| France | 27,101.13 (14,895.57, 44,281.09) | 367.55 (200.81, 601.53) | 39,191.92 (21,728.18, 64,416.12) | 365.82 (201.74, 603.12) | −0.05 (−0.09, 0.00) |
| Gabon | 233.33 (127.31, 377.14) | 415.64 (227.55, 671.82) | 406.21 (220.96, 656.47) | 415.64 (227.55, 671.82) | 0.00 (0.00, 0.00) |
| Georgia | 2930.44 (1606.82, 4762.87) | 439.31 (240.51, 713.12) | 2566.57 (1407.48, 4168.53) | 439.31 (240.51, 713.12) | 0.00 (0.00, 0.00) |
| Germany | 42,333.05 (22,629.64, 69,530.69) | 368.74 (195.23, 606.71) | 57,228.95 (30,969.59, 93,589.73) | 365.65 (195.91, 600.31) | 0.00 (−0.01, 0.02) |
| Ghana | 2458.97 (1305.55, 3994.53) | 440.97 (236.06, 715.48) | 7156.82 (3802.57, 11,623.67) | 440.97 (236.06, 715.48) | 0.00 (0.00, 0.00) |
| Greece | 5230.31 (2876.99, 8614.87) | 370.08 (203.30, 609.94) | 6606.04 (3554.01, 10,947.83) | 370.82 (197.50, 616.87) | 0.01 (0.01, 0.01) |
| Greenland | 11.83 (6.40, 19.48) | 385.68 (209.10, 634.10) | 27.02 (14.60, 44.47) | 385.68 (209.10, 634.10) | 0.00 (0.00, 0.00) |
| Grenada | 26.68 (14.64, 43.25) | 407.48 (222.23, 660.74) | 43.14 (23.51, 69.95) | 407.48 (222.23, 660.74) | 0.00 (0.00, 0.00) |
| Guam | 34.12 (18.01, 54.61) | 505.44 (269.49, 808.58) | 97.39 (51.92, 155.76) | 505.44 (269.49, 808.58) | 0.00 (0.00, 0.00) |
| Guatemala | 1264.84 (690.27, 2026.82) | 413.08 (226.25, 661.97) | 4122.27 (2255.29, 6605.92) | 413.08 (226.25, 661.97) | 0.00 (0.00, 0.00) |
| Guinea | 1222.21 (652.03, 1982.89) | 440.97 (236.06, 715.48) | 2038.95 (1084.82, 3311.95) | 440.97 (236.06, 715.48) | 0.00 (0.00, 0.00) |
| Guinea-Bissau | 151.06 (80.19, 245.38) | 440.97 (236.06, 715.48) | 294.59 (156.08, 478.76) | 440.97 (236.06, 715.48) | 0.00 (0.00, 0.00) |
| Guyana | 133.77 (72.74, 217.00) | 407.48 (222.23, 660.74) | 256.95 (139.50, 416.98) | 407.48 (222.23, 660.74) | 0.00 (0.00, 0.00) |
| Haiti | 1182.01 (641.76, 1918.18) | 407.48 (222.23, 660.74) | 2736.62 (1483.21, 4442.26) | 407.48 (222.23, 660.74) | 0.00 (0.00, 0.00) |
| Honduras | 733.91 (400.99, 1175.75) | 413.08 (226.25, 661.97) | 2403.09 (1313.98, 3850.90) | 413.08 (226.25, 661.97) | 0.00 (0.00, 0.00) |
| Hungary | 6521.75 (3542.77, 10,611.73) | 447.98 (243.49, 730.25) | 7326.57 (4005.85, 11,920.55) | 447.98 (243.49, 730.25) | 0.00 (0.00, 0.00) |
| Iceland | 88.03 (47.99, 143.05) | 368.35 (200.35, 598.41) | 172.90 (94.26, 280.98) | 368.35 (200.35, 598.41) | 0.00 (0.00, 0.00) |
| India | 190,874.29 (117,109.16, 286,671.42) | 477.24 (293.89, 714.28) | 509,370.86 (308,392.06, 752,137.88) | 476.76 (289.21, 703.69) | 0.00 (0.00, 0.01) |
| Indonesia | 48,945.97 (29,786.73, 73,128.11) | 538.03 (329.15, 802.34) | 124,858.36 (75,983.98, 186,508.74) | 538.03 (329.15, 802.34) | 0.00 (0.00, 0.00) |
| Iran | 10,629.36 (6552.31, 15,826.73) | 459.62 (285.16, 683.06) | 32,959.91 (20,066.24, 49,014.70) | 484.18 (295.86, 719.02) | 0.11 (0.06, 0.15) |
| Iraq | 2838.34 (1578.86, 4611.97) | 430.01 (239.34, 698.54) | 9037.84 (5025.82, 14,697.94) | 430.01 (239.34, 698.54) | 0.00 (0.00, 0.00) |
| Ireland | 1245.22 (678.97, 2026.74) | 368.35 (200.35, 598.41) | 2377.30 (1296.35, 3863.25) | 368.35 (200.35, 598.41) | 0.00 (0.00, 0.00) |
| Israel | 1544.24 (840.15, 2513.78) | 368.35 (200.35, 598.41) | 3602.97 (1964.27, 5860.19) | 368.35 (200.35, 598.41) | 0.00 (0.00, 0.00) |
| Italy | 31,515.40 (19,037.97, 47,433.81) | 389.85 (234.15, 588.23) | 43,983.72 (26,940.93, 65,938.28) | 393.83 (238.72, 592.86) | 0.13 (0.08, 0.18) |
| Jamaica | 612.21 (335.35, 991.85) | 407.48 (222.23, 660.74) | 1087.80 (593.70, 1764.98) | 407.48 (222.23, 660.74) | 0.00 (0.00, 0.00) |
| Japan | 45,105.54 (27,421.53, 67,657.35) | 278.64 (169.19, 418.17) | 70,106.89 (43,430.93, 104,266.93) | 304.10 (185.17, 453.94) | 0.40 (0.33, 0.47) |
| Jordan | 483.70 (268.78, 786.74) | 430.01 (239.34, 698.54) | 2786.01 (1549.79, 4532.82) | 430.01 (239.34, 698.54) | 0.00 (0.00, 0.00) |
| Kazakhstan | 5679.42 (3114.98, 9235.06) | 439.31 (240.51, 713.12) | 8484.28 (4632.55, 13,787.43) | 439.31 (240.51, 713.12) | 0.00 (0.00, 0.00) |
| Kenya | 2686.48 (1650.82, 3986.96) | 378.98 (234.09, 561.60) | 7828.17 (4807.84, 11,617.15) | 378.98 (234.09, 561.60) | 0.00 (0.00, 0.00) |
| Kiribati | 17.60 (9.33, 28.16) | 505.44 (269.49, 808.58) | 39.02 (20.57, 62.44) | 505.44 (269.49, 808.58) | 0.00 (0.00, 0.00) |
| Kuwait | 156.75 (85.14, 254.60) | 429.81 (234.33, 696.99) | 943.07 (511.36, 1,531.40) | 429.83 (235.01, 695.29) | 0.00 (0.00, 0.00) |
| Kyrgyzstan | 1377.96 (754.50, 2239.13) | 439.31 (240.51, 713.12) | 2260.63 (1232.32, 3673.78) | 439.31 (240.51, 713.12) | 0.00 (0.00, 0.00) |
| Laos | 964.50 (515.71, 1560.06) | 505.38 (271.24, 816.86) | 2169.78 (1160.85, 3509.14) | 505.38 (271.24, 816.86) | 0.00 (0.00, 0.00) |
| Latvia | 1668.03 (928.89, 2677.51) | 430.98 (240.19, 691.93) | 1571.17 (877.34, 2521.70) | 430.98 (240.19, 691.93) | 0.00 (0.00, 0.00) |
| Lebanon | 860.18 (478.15, 1398.49) | 430.01 (239.34, 698.54) | 2267.98 (1263.63, 3682.53) | 430.01 (239.34, 698.54) | 0.00 (0.00, 0.00) |
| Lesotho | 378.57 (206.98, 611.73) | 415.64 (227.55, 671.82) | 483.62 (264.00, 781.49) | 415.64 (227.55, 671.82) | 0.00 (0.00, 0.00) |
| Liberia | 382.24 (204.18, 620.20) | 440.97 (236.06, 715.48) | 763.17 (404.13, 1240.20) | 440.97 (236.06, 715.48) | 0.00 (0.00, 0.00) |
| Libya | 653.95 (364.10, 1063.39) | 430.01 (239.34, 698.54) | 1920.70 (1069.07, 3124.51) | 430.01 (239.34, 698.54) | 0.00 (0.00, 0.00) |
| Lithuania | 2077.70 (1149.50, 3337.95) | 430.49 (238.11, 691.22) | 2330.49 (1297.41, 3745.10) | 430.49 (238.11, 691.22) | −0.01 (−0.01, 0.00) |
| Luxembourg | 184.39 (99.21, 301.22) | 363.56 (194.21, 594.56) | 320.92 (172.21, 524.31) | 363.56 (194.21, 594.56) | 0.00 (−0.01, 0.00) |
| Macedonia | 815.35 (441.27, 1328.62) | 447.98 (243.49, 730.25) | 1369.56 (744.36, 2231.77) | 447.98 (243.49, 730.25) | 0.00 (0.00, 0.00) |
| Madagascar | 1585.69 (868.06, 2526.53) | 361.47 (199.31, 575.10) | 3722.75 (2032.14, 5935.62) | 361.47 (199.31, 575.10) | 0.00 (0.00, 0.00) |
| Malawi | 1255.59 (687.75, 1999.80) | 361.47 (199.31, 575.10) | 2413.30 (1325.05, 3842.69) | 361.47 (199.31, 575.10) | 0.00 (0.00, 0.00) |
| Malaysia | 3847.42 (2175.87, 6174.89) | 484.96 (274.34, 777.96) | 12,541.91 (6843.56, 20,028.01) | 488.86 (267.55, 780.40) | 0.04 (0.03, 0.04) |
| Maldives | 35.82 (19.10, 57.96) | 505.38 (271.24, 816.86) | 136.44 (73.02, 220.59) | 505.38 (271.24, 816.86) | 0.00 (0.00, 0.00) |
| Mali | 1544.46 (819.09, 2509.28) | 440.97 (236.06, 715.48) | 3280.74 (1741.60, 5330.17) | 440.97 (236.06, 715.48) | 0.00 (0.00, 0.00) |
| Malta | 145.40 (79.06, 236.35) | 368.35 (200.35, 598.41) | 266.75 (145.92, 434.50) | 368.35 (200.35, 598.41) | 0.00 (0.00, 0.00) |
| Marshall Islands | 6.76 (3.60, 10.81) | 505.44 (269.49, 808.58) | 16.45 (8.63, 26.32) | 505.44 (269.49, 808.58) | 0.00 (0.00, 0.00) |
| Mauritania | 394.11 (210.31, 639.73) | 440.97 (236.06, 715.48) | 825.37 (439.07, 1340.29) | 440.97 (236.06, 715.48) | 0.00 (0.00, 0.00) |
| Mauritius | 340.21 (182.13, 550.47) | 505.38 (271.24, 816.86) | 928.97 (497.74, 1502.39) | 505.38 (271.24, 816.86) | 0.00 (0.00, 0.00) |
| Mexico | 16,143.81 (9957.88, 23,921.32) | 428.87 (264.95, 634.81) | 51,130.83 (31,287.19, 76,378.66) | 432.62 (265.13, 645.69) | 0.04 (0.03, 0.05) |
| Moldova | 2024.14 (1125.04, 3248.79) | 430.98 (240.19, 691.93) | 2607.68 (1450.90, 4185.55) | 430.98 (240.19, 691.93) | 0.00 (0.00, 0.00) |
| Monaco | 19.81 (10.83, 32.21) | 368.35 (200.35, 598.41) | 27.21 (14.90, 44.22) | 368.35 (200.35, 598.41) | 0.00 (0.00, 0.00) |
| Mongolia | 427.94 (233.85, 694.09) | 439.31 (240.51, 713.12) | 1110.33 (605.11, 1803.75) | 439.31 (240.51, 713.12) | 0.00 (0.00, 0.00) |
| Montenegro | 277.35 (150.61, 451.99) | 447.98 (243.49, 730.25) | 413.04 (224.59, 673.00) | 447.98 (243.49, 730.25) | 0.00 (0.00, 0.00) |
| Morocco | 5189.17 (2885.62, 8435.03) | 430.01 (239.34, 698.54) | 13,587.24 (7560.13, 22,093.73) | 430.01 (239.34, 698.54) | 0.00 (0.00, 0.00) |
| Mozambique | 1913.12 (1048.37, 3047.50) | 361.47 (199.31, 575.10) | 3747.31 (2053.50, 5968.74) | 361.47 (199.31, 575.10) | 0.00 (0.00, 0.00) |
| Myanmar | 11,152.90 (5964.29, 18,045.24) | 505.38 (271.24, 816.86) | 25,417.13 (13,595.97, 41,107.22) | 505.38 (271.24, 816.86) | 0.00 (0.00, 0.00) |
| Namibia | 254.43 (138.63, 410.84) | 415.64 (227.55, 671.82) | 573.27 (312.68, 926.56) | 415.64 (227.55, 671.82) | 0.00 (0.00, 0.00) |
| Nauru | 1.71 (0.90, 2.73) | 505.44 (269.49, 808.58) | 2.78 (1.47, 4.45) | 505.44 (269.49, 808.58) | 0.00 (0.00, 0.00) |
| Nepal | 3639.16 (1966.06, 5882.19) | 455.87 (247.86, 736.91) | 9827.52 (5365.11, 15,778.50) | 456.88 (249.79, 733.51) | 0.01 (0.01, 0.01) |
| Netherlands | 6387.82 (3474.54, 10,421.19) | 371.34 (201.11, 607.42) | 10,399.46 (5651.36, 17,143.27) | 368.94 (199.04, 609.87) | −0.02 (−0.06, 0.01) |
| New Zealand | 1235.43 (758.98, 1855.63) | 372.06 (227.70, 560.08) | 2645.31 (1623.82, 3977.29) | 372.06 (227.70, 560.08) | 0.00 (−0.01, 0.00) |
| Nicaragua | 559.52 (305.93, 896.45) | 413.08 (226.25, 661.97) | 1904.91 (1041.67, 3052.28) | 413.08 (226.25, 661.97) | 0.00 (0.00, 0.00) |
| Niger | 943.36 (501.06, 1532.08) | 440.97 (236.06, 715.48) | 3378.20 (1792.01, 5491.52) | 440.97 (236.06, 715.48) | 0.00 (0.00, 0.00) |
| Nigeria | 15,989.80 (9772.36, 23,982.78) | 470.20 (287.28, 706.22) | 42,789.09 (25,987.41, 64,365.72) | 471.30 (287.84, 705.92) | −0.01 (−0.02, 0.00) |
| Niue | 0.97 (0.52, 1.55) | 505.44 (269.49, 808.58) | 1.05 (0.56, 1.68) | 505.44 (269.49, 808.58) | 0.00 (0.00, 0.00) |
| North Korea | 8941.66 (4833.51, 14,312.76) | 512.03 (277.63, 820.15) | 16,846.73 (9158.14, 26,998.39) | 512.03 (277.63, 820.15) | 0.00 (0.00, 0.00) |
| Northern Mariana Islands | 5.34 (2.81, 8.55) | 505.44 (269.49, 808.58) | 28.47 (14.82, 45.60) | 505.44 (269.49, 808.58) | 0.00 (0.00, 0.00) |
| Norway | 2032.37 (1259.10, 3058.67) | 390.23 (239.80, 591.92) | 2954.72 (1809.36, 4438.34) | 387.73 (235.72, 584.50) | 0.01 (−0.01, 0.04) |
| Oman | 202.28 (112.79, 328.91) | 430.01 (239.34, 698.54) | 622.81 (346.68, 1013.14) | 430.01 (239.34, 698.54) | 0.00 (0.00, 0.00) |
| Pakistan | 19,465.40 (11,924.10, 28,997.84) | 466.62 (286.41, 694.86) | 46,683.00 (28,564.54, 69,603.15) | 466.62 (286.41, 694.86) | 0.02 (0.01, 0.03) |
| Palau | 4.18 (2.23, 6.68) | 505.44 (269.49, 808.58) | 11.56 (6.08, 18.50) | 505.44 (269.49, 808.58) | 0.00 (0.00, 0.00) |
| Palestine | 357.37 (198.72, 580.99) | 430.01 (239.34, 698.54) | 1006.69 (560.25, 1637.49) | 430.01 (239.34, 698.54) | 0.00 (0.00, 0.00) |
| Panama | 498.03 (272.73, 798.05) | 413.08 (226.25, 661.97) | 1567.24 (858.72, 2511.08) | 413.08 (226.25, 661.97) | 0.00 (0.00, 0.00) |
| Papua New Guinea | 778.65 (410.13, 1245.81) | 505.44 (269.49, 808.58) | 2138.76 (1120.79, 3423.81) | 505.44 (269.49, 808.58) | 0.00 (0.00, 0.00) |
| Paraguay | 800.54 (436.24, 1314.13) | 419.70 (228.82, 688.82) | 2194.64 (1194.39, 3603.45) | 419.70 (228.82, 688.82) | 0.00 (0.00, 0.00) |
| Peru | 3336.99 (1867.60, 5328.24) | 321.56 (180.32, 513.22) | 10,011.34 (5617.09, 16,071.69) | 342.88 (192.57, 550.13) | 0.22 (0.16, 0.29) |
| Philippines | 14,242.18 (8681.71, 21,267.25) | 538.03 (329.15, 802.34) | 42,204.58 (25,747.45, 62,997.67) | 538.03 (329.15, 802.34) | 0.00 (0.00, 0.00) |
| Poland | 20,950.56 (12,700.41, 31,022.60) | 474.10 (287.14, 702.03) | 29,389.27 (17,858.13, 43,479.65) | 474.10 (287.14, 702.03) | 0.00 (0.00, 0.00) |
| Portugal | 4849.26 (2640.17, 7889.43) | 368.35 (200.35, 598.41) | 7175.04 (3924.75, 11,651.31) | 368.35 (200.35, 598.41) | 0.00 (0.00, 0.00) |
| Puerto Rico | 1306.14 (713.72, 2116.17) | 407.48 (222.23, 660.74) | 2397.40 (1317.62, 3881.02) | 407.48 (222.23, 660.74) | 0.00 (0.00, 0.00) |
| Qatar | 24.40 (13.56, 39.72) | 430.01 (239.34, 698.54) | 239.06 (133.02, 389.42) | 430.01 (239.34, 698.54) | 0.00 (0.00, 0.00) |
| Romania | 12,600.39 (6829.77, 20,517.39) | 447.98 (243.49, 730.25) | 13,428.28 (7337.85, 21,850.42) | 447.98 (243.49, 730.25) | 0.00 (0.00, 0.00) |
| Russian Federation | 92,169.86 (56,642.77, 136,545.05) | 457.88 (281.55, 677.44) | 113,062.09 (69,459.76, 167,289.29) | 457.88 (281.55, 677.44) | 0.00 (0.00, 0.00) |
| Rwanda | 990.43 (541.85, 1578.08) | 361.47 (199.31, 575.10) | 2305.14 (1262.99, 3671.91) | 361.47 (199.31, 575.10) | 0.00 (0.00, 0.00) |
| Saint Kitts and Nevis | 13.66 (7.51, 22.11) | 407.48 (222.23, 660.74) | 28.93 (15.68, 46.97) | 407.48 (222.23, 660.74) | 0.00 (0.00, 0.00) |
| Saint Lucia | 32.60 (17.82, 52.83) | 407.48 (222.23, 660.74) | 90.30 (49.23, 146.48) | 407.48 (222.23, 660.74) | 0.00 (0.00, 0.00) |
| Saint Vincent and the Grenadines | 26.43 (14.46, 42.82) | 407.48 (222.23, 660.74) | 50.83 (27.69, 82.46) | 407.48 (222.23, 660.74) | 0.00 (0.00, 0.00) |
| Samoa | 38.09 (20.18, 60.92) | 505.44 (269.49, 808.58) | 65.55 (34.75, 104.90) | 505.44 (269.49, 808.58) | 0.00 (0.00, 0.00) |
| San Marino | 10.83 (5.91, 17.61) | 368.35 (200.35, 598.41) | 21.43 (11.71, 34.75) | 368.35 (200.35, 598.41) | 0.00 (0.00, 0.00) |
| Sao Tome and Principe | 26.38 (14.10, 42.82) | 440.97 (236.06, 715.48) | 44.52 (23.66, 72.33) | 440.97 (236.06, 715.48) | 0.00 (0.00, 0.00) |
| Saudi Arabia | 1620.03 (884.16, 2640.54) | 423.46 (231.41, 689.07) | 5884.91 (3182.59, 9621.58) | 423.46 (231.41, 689.07) | 0.02 (0.01, 0.02) |
| Senegal | 1179.67 (628.25, 1915.66) | 440.97 (236.06, 715.48) | 3104.76 (1651.59, 5043.03) | 440.97 (236.06, 715.48) | 0.00 (0.00, 0.00) |
| Serbia | 5264.16 (2845.46, 8579.03) | 447.98 (243.49, 730.25) | 6338.78 (3454.13, 10,316.19) | 447.98 (243.49, 730.25) | 0.00 (0.00, 0.00) |
| Seychelles | 26.45 (14.22, 42.74) | 505.38 (271.24, 816.86) | 56.65 (30.36, 91.58) | 505.38 (271.24, 816.86) | 0.00 (0.00, 0.00) |
| Sierra Leone | 714.91 (382.28, 1160.04) | 440.97 (236.06, 715.48) | 1365.32 (727.54, 2217.32) | 440.97 (236.06, 715.48) | 0.00 (0.00, 0.00) |
| Singapore | 539.58 (300.70, 856.55) | 266.07 (148.30, 422.03) | 2093.79 (1168.82, 3374.77) | 276.04 (154.16, 445.12) | 0.14 (0.10, 0.17) |
| Slovakia | 2601.18 (1410.86, 4232.28) | 447.98 (243.49, 730.25) | 3857.77 (2102.32, 6281.04) | 447.98 (243.49, 730.25) | 0.00 (0.00, 0.00) |
| Slovenia | 1140.32 (619.21, 1856.38) | 447.98 (243.49, 730.25) | 1602.22 (875.71, 2609.49) | 447.98 (243.49, 730.25) | 0.00 (0.00, 0.00) |
| Solomon Islands | 55.92 (29.32, 89.50) | 505.44 (269.49, 808.58) | 147.59 (77.78, 236.27) | 505.44 (269.49, 808.58) | 0.00 (0.00, 0.00) |
| Somalia | 756.25 (412.87, 1205.69) | 361.47 (199.31, 575.10) | 2114.97 (1155.41, 3370.13) | 361.47 (199.31, 575.10) | 0.00 (0.00, 0.00) |
| South Africa | 8723.06 (5319.68, 13,089.50) | 437.39 (267.33, 655.56) | 20,632.43 (12,575.85, 30,961.65) | 437.39 (267.33, 655.56) | 0.00 (0.00, 0.00) |
| South Korea | 9161.58 (5003.66, 15,079.21) | 298.75 (163.68, 491.06) | 26,014.80 (14,201.97, 42,478.06) | 299.23 (163.15, 488.25) | 0.00 (0.00, 0.01) |
| South Sudan | 596.50 (328.50, 949.14) | 361.47 (199.31, 575.10) | 1143.09 (624.79, 1822.28) | 361.47 (199.31, 575.10) | 0.00 (0.00, 0.00) |
| Spain | 18,860.82 (10,297.56, 31,200.87) | 371.50 (202.34, 615.72) | 28,621.46 (15,803.75, 47,382.92) | 371.52 (203.64, 618.31) | 0.00 (0.00, 0.00) |
| Sri Lanka | 4729.81 (2531.30, 7649.42) | 505.38 (271.24, 816.86) | 13,492.05 (7234.41, 21,821.90) | 505.38 (271.24, 816.86) | 0.00 (0.00, 0.00) |
| Sudan | 3084.42 (1716.84, 5013.34) | 430.01 (239.34, 698.54) | 6468.91 (3598.33, 10,520.49) | 430.01 (239.34, 698.54) | 0.00 (0.00, 0.00) |
| Suriname | 94.60 (51.39, 153.55) | 407.48 (222.23, 660.74) | 250.83 (136.56, 406.94) | 407.48 (222.23, 660.74) | 0.00 (0.00, 0.00) |
| Swaziland | 107.56 (58.69, 173.77) | 415.64 (227.55, 671.82) | 239.46 (130.60, 386.94) | 415.64 (227.55, 671.82) | 0.00 (0.00, 0.00) |
| Sweden | 4502.86 (2739.79, 6753.92) | 395.65 (237.70, 596.20) | 6067.80 (3671.49, 9135.23) | 388.99 (232.88, 588.49) | −0.08 (−0.09, −0.06) |
| Switzerland | 3180.04 (1737.05, 5167.58) | 360.63 (195.40, 588.45) | 5189.68 (2885.12, 8355.46) | 362.07 (200.29, 584.04) | 0.01 (0.00, 0.02) |
| Syria | 1923.99 (1069.85, 3130.03) | 430.01 (239.34, 698.54) | 5340.35 (2968.29, 8687.10) | 430.01 (239.34, 698.54) | 0.00 (0.00, 0.00) |
| Taiwan (Province of China) | 7025.06 (3883.35, 11,367.79) | 532.35 (294.78, 861.62) | 20,361.95 (11,215.47, 33,128.00) | 521.26 (287.54, 848.06) | −0.09 (−0.11, −0.08) |
| Tajikistan | 1198.71 (655.11, 1946.48) | 439.31 (240.51, 713.12) | 2620.61 (1426.03, 4259.80) | 439.31 (240.51, 713.12) | 0.00 (0.00, 0.00) |
| Tanzania | 3467.40 (1906.59, 5584.08) | 363.61 (200.67, 585.81) | 8219.52 (4483.28, 13,182.18) | 369.13 (202.52, 590.73) | −0.01 (−0.06, 0.04) |
| Thailand | 16,777.33 (9025.10, 27,362.78) | 496.19 (267.97, 810.20) | 54,107.69 (29,733.78, 88,068.49) | 511.09 (281.03, 831.84) | 0.10 (0.07, 0.13) |
| The Bahamas | 56.62 (30.90, 91.79) | 407.48 (222.23, 660.74) | 168.56 (91.56, 273.52) | 407.48 (222.23, 660.74) | 0.00 (0.00, 0.00) |
| The Gambia | 115.14 (61.49, 186.91) | 440.97 (236.06, 715.48) | 357.46 (190.77, 580.27) | 440.97 (236.06, 715.48) | 0.00 (0.00, 0.00) |
| Timor-Leste | 120.99 (64.56, 195.71) | 505.38 (271.24, 816.86) | 356.95 (191.22, 577.69) | 505.38 (271.24, 816.86) | 0.00 (0.00, 0.00) |
| Togo | 475.20 (252.23, 772.00) | 440.97 (236.06, 715.48) | 1661.08 (881.74, 2698.85) | 440.97 (236.06, 715.48) | 0.00 (0.00, 0.00) |
| Tokelau | 0.60 (0.32, 0.96) | 505.44 (269.49, 808.58) | 0.64 (0.34, 1.02) | 505.44 (269.49, 808.58) | 0.00 (0.00, 0.00) |
| Tonga | 25.72 (13.61, 41.16) | 505.44 (269.49, 808.58) | 35.94 (19.13, 57.49) | 505.44 (269.49, 808.58) | 0.00 (0.00, 0.00) |
| Trinidad and Tobago | 290.70 (158.69, 471.02) | 407.48 (222.23, 660.74) | 735.55 (401.06, 1192.51) | 407.48 (222.23, 660.74) | 0.00 (0.00, 0.00) |
| Tunisia | 1906.85 (1060.52, 3101.17) | 430.01 (239.34, 698.54) | 5255.27 (2922.90, 8540.97) | 430.01 (239.34, 698.54) | 0.00 (0.00, 0.00) |
| Turkey | 14,329.90 (7875.22, 22,999.93) | 434.22 (239.50, 697.15) | 38,286.01 (20,876.45, 62,476.30) | 432.50 (236.24, 705.86) | 0.00 (−0.01, 0.00) |
| Turkmenistan | 862.80 (471.92, 1401.85) | 439.31 (240.51, 713.12) | 1882.35 (1025.66, 3057.50) | 439.31 (240.51, 713.12) | 0.00 (0.00, 0.00) |
| Tuvalu | 3.41 (1.81, 5.46) | 505.44 (269.49, 808.58) | 5.07 (2.70, 8.11) | 505.44 (269.49, 808.58) | 0.00 (0.00, 0.00) |
| Uganda | 1996.63 (1096.71, 3179.89) | 361.47 (199.31, 575.10) | 5051.55 (2771.66, 8046.14) | 361.47 (199.31, 575.10) | 0.00 (0.00, 0.00) |
| Ukraine | 34,752.18 (21,259.07, 51,792.05) | 454.17 (277.73, 676.71) | 35,747.34 (21,895.77, 53,217.28) | 454.17 (277.73, 676.71) | 0.00 (0.00, 0.00) |
| United Arab Emirates | 96.78 (53.54, 158.00) | 429.99 (238.27, 701.22) | 884.20 (488.94, 1,439.79) | 430.01 (239.11, 697.87) | 0.00 (0.00, 0.00) |
| United Kingdom | 28,885.66 (17,542.30, 43,567.90) | 383.67 (231.21, 580.05) | 39,389.77 (23,777.72, 59,182.45) | 383.42 (229.88, 577.06) | 0.00 (0.00, 0.00) |
| United States | 110,478.29 (66,699.81, 164,043.66) | 409.82 (245.56, 609.67) | 206,920.98 (126,735.47, 309,100.21) | 412.45 (252.21, 616.65) | 0.06 (0.03, 0.10) |
| Uruguay | 1260.39 (691.49, 2038.92) | 349.99 (191.61, 566.26) | 1651.05 (908.88, 2667.04) | 349.99 (191.61, 566.26) | 0.00 (0.00, 0.00) |
| Uzbekistan | 5094.42 (2788.95, 8276.34) | 439.31 (240.51, 713.12) | 12,150.67 (6615.05, 19,736.70) | 439.31 (240.51, 713.12) | 0.00 (0.00, 0.00) |
| Vanuatu | 24.43 (12.90, 39.08) | 505.44 (269.49, 808.58) | 79.89 (42.11, 127.77) | 505.44 (269.49, 808.58) | 0.00 (0.00, 0.00) |
| Venezuela | 3476.68 (1921.55, 5593.08) | 411.27 (227.45, 661.03) | 11,724.52 (6425.73, 18,978.47) | 412.95 (226.64, 668.25) | 0.02 (0.02, 0.02) |
| Vietnam | 20,572.19 (11,023.76, 33,259.93) | 505.38 (271.24, 816.86) | 51,652.86 (27,637.06, 83,539.29) | 505.38 (271.24, 816.86) | 0.00 (0.00, 0.00) |
| Virgin Islands, U.S. | 31.55 (17.17, 51.18) | 407.48 (222.23, 660.74) | 67.13 (36.81, 108.73) | 407.48 (222.23, 660.74) | 0.00 (0.00, 0.00) |
| Yemen | 1821.67 (1012.20, 2961.01) | 430.01 (239.34, 698.54) | 5239.80 (2911.74, 8519.41) | 430.01 (239.34, 698.54) | 0.00 (0.00, 0.00) |
| Zambia | 862.23 (474.35, 1383.41) | 371.03 (204.74, 595.12) | 2213.23 (1218.52, 3551.69) | 371.03 (204.74, 595.12) | −0.01 (−0.02, −0.01) |
| Zimbabwe | 1412.19 (770.32, 2280.58) | 415.64 (227.55, 671.82) | 2859.61 (1555.91, 4618.88) | 415.64 (227.55, 671.82) | 0.00 (0.00, 0.00) |

ASR: age-standardized rate; EAPC: estimated annual percentage change; CI: confidence interval.

Supplementary Table 4. The disability-adjusted life years (DALYs) of migraine in postmenopausal women in 204 countries and regions from 1990 to 2021.

| Location | 1990 | | 2023 | | EAPC (95% CI) |
| --- | --- | --- | --- | --- | --- |
|  | Number | ASR | Number | ASR |  |
| Afghanistan | 3639.11 (871.70, 7652.30) | 593.97 (144.94, 1250.64) | 4291.90 (1066.84, 8982.40) | 577.33 (146.69, 1202.66) | −0.07 (−0.08, −0.07) |
| Albania | 1153.15 (337.52, 2451.02) | 639.49 (187.30, 1359.54) | 2591.79 (770.56, 5535.38) | 638.95 (189.45, 1365.84) | 0.01 (0.00, 0.01) |
| Algeria | 6768.78 (1678.56, 14,151.63) | 600.56 (150.49, 1254.98) | 18,592.37 (4631.35, 39,072.65) | 593.78 (149.16, 1248.01) | −0.03 (−0.03, −0.02) |
| American Samoa | 10.74 (1.86, 24.12) | 561.74 (97.71, 1262.83) | 24.31 (4.16, 54.19) | 548.78 (94.79, 1223.49) | −0.07 (−0.07, −0.07) |
| Andorra | 30.71 (7.41, 65.45) | 640.91 (153.82, 1366.44) | 80.76 (19.24, 173.25) | 637.93 (152.25, 1368.66) | −0.02 (−0.02, −0.02) |
| Angola | 1712.71 (444.76, 3599.95) | 507.15 (133.59, 1063.70) | 5910.39 (1545.46, 12,459.62) | 507.10 (134.65, 1068.86) | 0.01 (0.00, 0.01) |
| Antigua and Barbuda | 24.36 (5.76, 52.54) | 539.15 (125.46, 1162.99) | 54.48 (12.63, 118.21) | 532.64 (124.73, 1156.73) | −0.04 (−0.04, −0.04) |
| Argentina | 13,814.56 (3884.58, 28,696.94) | 450.97 (126.27, 936.70) | 22,632.01 (6311.48, 47,969.42) | 459.27 (126.93, 973.19) | 0.09 (0.07, 0.12) |
| Armenia | 1702.82 (454.44, 3655.31) | 586.72 (157.60, 1263.68) | 2622.29 (702.59, 5733.86) | 583.00 (154.97, 1278.08) | −0.01 (−0.01, 0.00) |
| Australia | 8359.68 (2211.83, 17,680.70) | 496.54 (128.96, 1050.49) | 18,055.36 (4854.32, 38,735.62) | 496.69 (131.76, 1065.79) | −0.02 (−0.02, −0.01) |
| Austria | 6413.24 (1789.12, 13,313.07) | 602.20 (163.82, 1251.94) | 8782.35 (2268.24, 18,119.22) | 600.18 (152.88, 1238.42) | 0.14 (0.03, 0.25) |
| Azerbaijan | 3129.35 (842.54, 6747.34) | 590.93 (159.28, 1273.80) | 6756.75 (1821.25, 14,508.05) | 584.60 (157.81, 1259.08) | −0.02 (−0.02, −0.01) |
| Bahrain | 83.13 (21.14, 176.10) | 593.65 (153.31, 1256.61) | 426.39 (106.36, 891.60) | 583.82 (148.78, 1225.16) | −0.05 (−0.05, −0.04) |
| Bangladesh | 18,657.48 (3870.13, 40,246.28) | 528.12 (111.04, 1140.03) | 61,808.42 (12,755.25, 134,423.52) | 529.50 (110.08, 1151.38) | 0.01 (0.00, 0.01) |
| Barbados | 134.64 (31.89, 292.77) | 539.13 (124.71, 1169.66) | 260.84 (60.69, 565.66) | 532.66 (123.09, 1154.99) | −0.03 (−0.04, −0.03) |
| Belarus | 10,251.42 (3659.55, 19,691.30) | 702.25 (250.30, 1351.07) | 11,992.67 (4291.14, 23,106.59) | 700.98 (251.07, 1351.31) | 0.01 (0.00, 0.02) |
| Belgium | 10,490.85 (2132.12, 22,585.41) | 753.10 (150.99, 1619.69) | 15,748.09 (2828.60, 35,294.73) | 855.62 (151.21, 1919.10) | 0.45 (0.32, 0.58) |
| Belize | 40.90 (9.24, 87.87) | 541.33 (122.20, 1162.59) | 137.98 (31.38, 301.94) | 533.30 (122.94, 1167.30) | −0.04 (−0.04, −0.03) |
| Benin | 945.95 (227.01, 2017.42) | 558.75 (134.28, 1192.11) | 2565.84 (589.49, 5488.17) | 562.50 (130.19, 1202.94) | 0.02 (0.01, 0.03) |
| Bermuda | 31.97 (7.33, 68.82) | 541.96 (123.94, 1166.64) | 65.27 (15.49, 141.56) | 540.35 (126.77, 1171.07) | −0.01 (−0.02, −0.01) |
| Bhutan | 112.37 (22.79, 246.26) | 533.54 (110.41, 1167.74) | 266.04 (55.44, 580.47) | 534.83 (111.70, 1166.97) | 0.01 (0.00, 0.01) |
| Bolivia | 1302.90 (347.84, 2718.12) | 449.88 (120.88, 938.68) | 3760.51 (1032.30, 7815.05) | 448.60 (123.49, 932.50) | −0.01 (−0.02, −0.01) |
| Bosnia and Herzegovina | 2790.49 (810.87, 5900.17) | 634.49 (184.74, 1345.32) | 3659.85 (1075.41, 7915.31) | 626.45 (183.31, 1355.01) | −0.03 (−0.03, −0.02) |
| Botswana | 278.06 (72.50, 587.64) | 512.20 (134.12, 1083.58) | 731.21 (189.54, 1546.13) | 501.59 (131.25, 1060.20) | −0.05 (−0.06, −0.05) |
| Brazil | 47,839.18 (9999.08, 105,110.25) | 585.06 (123.25, 1285.31) | 140,410.78 (29,723.40, 308,045.59) | 587.25 (124.51, 1288.41) | −0.04 (−0.10, 0.02) |
| Brunei | 31.92 (8.95, 68.10) | 403.46 (114.16, 857.53) | 133.18 (37.00, 286.42) | 401.57 (113.09, 862.47) | −0.01 (−0.02, 0.00) |
| Bulgaria | 7863.84 (2348.76, 16,701.28) | 634.48 (188.85, 1349.29) | 7785.97 (2326.97, 16,612.05) | 630.41 (187.95, 1345.41) | 0.00 (−0.01, 0.00) |
| Burkina Faso | 2209.92 (520.15, 4737.67) | 564.22 (133.85, 1209.03) | 4701.01 (1119.21, 10,202.50) | 568.24 (136.96, 1232.35) | 0.03 (0.03, 0.04) |
| Burundi | 899.77 (285.59, 1838.42) | 420.01 (133.63, 858.98) | 1707.11 (548.83, 3523.70) | 422.19 (136.10, 871.06) | 0.04 (0.03, 0.04) |
| Cambodia | 2727.85 (421.39, 6069.09) | 603.70 (94.34, 1346.86) | 8074.63 (1270.24, 18,181.66) | 605.53 (96.16, 1366.44) | 0.02 (0.01, 0.02) |
| Cameroon | 2247.40 (526.21, 4804.14) | 560.54 (132.75, 1198.13) | 6260.71 (1513.88, 13,226.30) | 561.42 (137.08, 1189.41) | 0.02 (0.01, 0.02) |
| Canada | 20,062.85 (3790.12, 44,208.05) | 704.06 (131.09, 1552.18) | 41,486.40 (8056.59, 92,281.86) | 677.85 (130.21, 1508.29) | −0.14 (−0.18, −0.10) |
| Cape Verde | 127.06 (30.53, 272.31) | 572.23 (137.78, 1224.58) | 252.14 (60.19, 536.86) | 566.15 (135.51, 1207.01) | −0.03 (−0.03, −0.02) |
| Central African Republic | 551.30 (144.08, 1164.25) | 500.07 (132.12, 1056.14) | 1078.33 (293.00, 2288.63) | 500.91 (137.75, 1063.49) | 0.02 (0.01, 0.02) |
| Chad | 1368.68 (335.85, 2949.17) | 562.88 (138.41, 1212.16) | 2484.41 (588.90, 5365.14) | 560.89 (134.66, 1209.21) | −0.01 (−0.01, 0.00) |
| Chile | 4607.34 (1207.56, 9700.04) | 493.97 (129.60, 1040.16) | 11,950.06 (3089.15, 25,406.96) | 495.38 (127.83, 1053.29) | 0.04 (0.03, 0.05) |
| China | 376,049.61 (67,167.42, 834,343.14) | 495.77 (88.84, 1100.08) | 1,068,764.05 (174,739.78, 2,418,056.68) | 541.18 (88.73, 1224.30) | 0.37 (0.30, 0.43) |
| Colombia | 8815.73 (1908.65, 18,957.32) | 572.70 (124.80, 1230.72) | 29,976.80 (6697.55, 64,067.01) | 569.52 (127.60, 1217.48) | −0.01 (−0.02, 0.00) |
| Comoros | 72.97 (22.63, 150.31) | 419.50 (130.50, 864.04) | 191.43 (60.00, 394.68) | 420.17 (131.90, 865.84) | 0.01 (0.00, 0.02) |
| Congo | 546.90 (144.30, 1149.57) | 507.38 (134.84, 1067.97) | 1203.03 (310.95, 2494.34) | 503.56 (131.89, 1046.20) | −0.01 (−0.02, −0.01) |
| Cook Islands | 5.83 (1.00, 13.16) | 561.26 (97.32, 1266.46) | 13.26 (2.27, 30.03) | 553.89 (95.17, 1253.39) | −0.04 (−0.05, −0.04) |
| Costa Rica | 817.43 (188.14, 1759.50) | 553.81 (127.79, 1191.99) | 2867.87 (651.20, 6136.89) | 549.54 (125.10, 1175.80) | −0.01 (−0.02, −0.01) |
| Cote d’Ivoire | 1772.07 (415.74, 3779.64) | 553.49 (132.08, 1179.93) | 5148.41 (1255.95, 11,093.41) | 563.73 (139.89, 1214.72) | 0.05 (0.04, 0.06) |
| Croatia | 4260.22 (1257.68, 9029.45) | 633.89 (186.19, 1344.89) | 4820.02 (1450.31, 10,122.85) | 629.74 (188.47, 1319.85) | −0.04 (−0.08, 0.00) |
| Cuba | 4560.31 (1014.59, 9836.51) | 538.26 (119.17, 1160.43) | 9650.31 (2227.19, 21,023.51) | 537.08 (124.54, 1169.05) | 0.00 (−0.01, 0.00) |
| Cyprus | 471.29 (111.84, 1005.03) | 637.65 (149.90, 1360.65) | 1109.78 (271.35, 2355.96) | 637.92 (153.82, 1353.74) | 0.00 (0.00, 0.01) |
| Czech Republic | 8407.86 (2492.55, 17,979.64) | 630.54 (185.67, 1346.51) | 10,903.25 (3268.41, 23,043.34) | 628.50 (189.50, 1327.59) | −0.01 (−0.01, 0.00) |
| Democratic Republic of the Congo | 7853.74 (2093.85, 16,300.26) | 500.50 (134.75, 1041.07) | 17,715.40 (4746.89, 36,475.76) | 502.45 (135.79, 1034.68) | 0.03 (0.02, 0.03) |
| Denmark | 3617.98 (959.32, 7588.37) | 550.96 (141.68, 1158.39) | 5153.89 (1346.77, 10,821.51) | 562.60 (144.43, 1181.51) | 0.08 (0.06, 0.10) |
| Djibouti | 47.52 (14.99, 97.36) | 422.09 (134.41, 868.07) | 209.04 (66.96, 430.53) | 420.39 (135.74, 864.52) | 0.01 (0.00, 0.01) |
| Dominica | 31.64 (7.46, 68.75) | 539.04 (125.85, 1170.53) | 40.54 (9.53, 87.78) | 531.86 (125.26, 1151.37) | −0.03 (−0.04, −0.03) |
| Dominican Republic | 1728.49 (403.00, 3720.25) | 540.74 (127.23, 1164.06) | 4656.34 (1073.62, 10,105.48) | 534.41 (123.49, 1159.49) | −0.03 (−0.04, −0.03) |
| Ecuador | 2273.47 (546.08, 4870.97) | 507.98 (122.61, 1087.41) | 7286.68 (1818.98, 15,696.44) | 500.38 (125.08, 1077.58) | −0.06 (−0.07, −0.04) |
| Egypt | 14,774.41 (3591.22, 31,490.03) | 614.68 (151.88, 1309.72) | 33,253.34 (8514.07, 68,539.34) | 579.36 (151.22, 1193.28) | −0.22 (−0.26, −0.19) |
| El Salvador | 1473.07 (334.50, 3181.74) | 555.62 (126.64, 1199.86) | 3216.82 (732.38, 6988.34) | 550.60 (125.31, 1195.64) | −0.01 (−0.02, 0.00) |
| Equatorial Guinea | 93.58 (24.64, 198.40) | 499.78 (133.23, 1058.82) | 245.78 (63.48, 519.90) | 502.90 (132.07, 1062.81) | 0.03 (0.02, 0.04) |
| Eritrea | 469.12 (148.04, 961.02) | 419.24 (133.47, 858.69) | 1175.71 (370.49, 2402.21) | 417.92 (132.21, 854.15) | 0.00 (0.00, 0.01) |
| Estonia | 1558.90 (565.07, 2966.12) | 700.99 (254.29, 1331.58) | 1644.10 (595.41, 3175.31) | 701.29 (255.74, 1348.60) | 0.01 (0.01, 0.02) |
| Ethiopia | 6144.18 (1922.89, 12,823.03) | 379.22 (118.75, 791.68) | 13,499.82 (4027.14, 28,292.24) | 385.26 (115.48, 806.19) | 0.08 (0.07, 0.09) |
| Federated States of Micronesia | 23.21 (4.00, 51.93) | 562.19 (96.59, 1258.45) | 41.32 (7.15, 92.43) | 554.61 (96.83, 1241.92) | −0.05 (−0.05, −0.04) |
| Fiji | 170.93 (28.69, 385.90) | 559.70 (95.11, 1265.57) | 422.64 (71.66, 952.30) | 551.90 (94.48, 1245.61) | −0.03 (−0.03, −0.02) |
| Finland | 4149.56 (1037.50, 8857.37) | 638.74 (155.59, 1364.45) | 6070.90 (1516.63, 12,868.90) | 637.60 (154.02, 1352.74) | 0.00 (−0.01, 0.01) |
| France | 47,960.26 (13,864.51, 97,864.26) | 652.61 (186.26, 1329.44) | 70,232.34 (20,396.93, 145,260.81) | 651.31 (185.42, 1352.94) | −0.02 (−0.04, −0.01) |
| Gabon | 285.10 (75.97, 606.34) | 506.19 (135.57, 1076.17) | 490.24 (129.59, 1039.87) | 503.32 (134.25, 1068.33) | −0.01 (−0.01, 0.00) |
| Georgia | 3960.59 (1044.86, 8613.21) | 589.46 (155.06, 1283.30) | 3459.38 (905.42, 7451.53) | 581.67 (151.54, 1253.48) | −0.04 (−0.06, −0.02) |
| Germany | 81,739.67 (20,588.98, 176,030.73) | 710.89 (175.38, 1529.07) | 110,549.59 (26,198.26, 233,376.04) | 723.58 (167.99, 1529.68) | 0.06 (0.01, 0.12) |
| Ghana | 3173.54 (746.45, 6815.65) | 567.54 (134.78, 1219.20) | 9224.84 (2179.32, 19,885.55) | 567.19 (135.04, 1223.49) | 0.01 (0.00, 0.01) |
| Greece | 9205.47 (2180.93, 19,661.81) | 652.19 (153.86, 1392.65) | 11,484.30 (2729.72, 24,853.92) | 655.36 (151.48, 1424.17) | 0.03 (0.02, 0.04) |
| Greenland | 19.75 (3.77, 43.32) | 652.63 (127.18, 1429.91) | 44.94 (8.76, 98.19) | 657.81 (131.54, 1437.19) | 0.04 (0.03, 0.05) |
| Grenada | 35.15 (8.22, 76.05) | 537.57 (123.98, 1161.14) | 55.72 (12.86, 119.77) | 532.32 (123.98, 1144.46) | −0.03 (−0.04, −0.03) |
| Guam | 38.18 (6.52, 85.29) | 568.91 (98.28, 1274.18) | 109.72 (18.55, 247.48) | 564.48 (95.51, 1272.82) | −0.02 (−0.02, −0.01) |
| Guatemala | 1675.00 (376.04, 3577.41) | 546.80 (124.06, 1168.82) | 5421.55 (1210.85, 11,746.75) | 542.91 (121.62, 1176.24) | −0.02 (−0.03, −0.02) |
| Guinea | 1585.06 (378.28, 3402.26) | 563.66 (134.40, 1210.61) | 2605.45 (621.42, 5544.00) | 564.31 (135.59, 1200.96) | 0.01 (0.01, 0.02) |
| Guinea-Bissau | 193.65 (45.82, 414.09) | 562.77 (134.46, 1204.08) | 375.74 (87.80, 800.47) | 563.35 (133.59, 1203.02) | 0.00 (0.00, 0.01) |
| Guyana | 173.06 (39.96, 372.75) | 525.60 (121.97, 1133.09) | 329.43 (76.00, 713.16) | 521.75 (121.66, 1130.82) | −0.01 (−0.02, −0.01) |
| Haiti | 1538.09 (348.68, 3360.78) | 528.67 (121.57, 1155.39) | 3514.33 (811.45, 7597.87) | 523.64 (122.65, 1132.11) | −0.03 (−0.03, −0.02) |
| Honduras | 981.78 (223.94, 2105.55) | 553.72 (127.22, 1187.01) | 3174.21 (712.71, 6829.96) | 545.47 (123.29, 1174.11) | −0.04 (−0.04, −0.03) |
| Hungary | 9252.69 (2744.11, 19,921.58) | 628.53 (185.51, 1351.86) | 10,579.79 (3139.79, 22,680.36) | 630.17 (185.34, 1351.33) | 0.02 (0.02, 0.03) |
| Iceland | 153.34 (37.44, 324.04) | 639.26 (154.24, 1349.96) | 304.15 (74.21, 644.35) | 641.85 (154.93, 1358.96) | 0.02 (0.01, 0.02) |
| India | 222,166.69 (48,206.06, 477,514.33) | 559.26 (122.98, 1201.27) | 601,738.96 (130,726.50, 1,307,301.08) | 562.44 (122.52, 1221.76) | 0.02 (0.02, 0.03) |
| Indonesia | 57,217.43 (9664.48, 127,192.85) | 632.64 (108.27, 1411.99) | 145,915.27 (24,252.46, 325,540.38) | 632.03 (106.42, 1414.66) | 0.01 (0.01, 0.01) |
| Iran | 14,895.07 (3738.94, 31,505.62) | 638.96 (163.05, 1351.75) | 46,690.59 (10,866.72, 101,805.76) | 684.17 (159.89, 1491.54) | 0.14 (0.07, 0.21) |
| Iraq | 3952.95 (964.34, 8429.40) | 598.22 (146.69, 1275.71) | 12,305.58 (3171.79, 25,934.86) | 586.26 (152.64, 1236.23) | −0.05 (−0.05, −0.04) |
| Ireland | 2191.10 (539.76, 4643.92) | 636.36 (153.42, 1347.11) | 4125.00 (967.61, 8742.85) | 637.76 (148.08, 1350.79) | 0.00 (0.00, 0.01) |
| Israel | 2740.31 (666.69, 5903.95) | 640.13 (153.29, 1380.09) | 6369.87 (1572.91, 13,623.22) | 639.43 (154.79, 1367.65) | 0.00 (0.00, 0.01) |
| Italy | 55,679.77 (12,495.54, 121,378.77) | 685.00 (152.15, 1493.21) | 79,220.01 (17,565.12, 171,778.36) | 721.84 (157.26, 1563.83) | 0.43 (0.35, 0.52) |
| Jamaica | 815.16 (191.18, 1765.66) | 540.69 (124.92, 1170.42) | 1439.75 (335.53, 3135.04) | 536.40 (125.19, 1168.15) | −0.02 (−0.02, −0.02) |
| Japan | 69,277.88 (20,690.75, 144,986.84) | 426.21 (126.81, 892.55) | 104,658.58 (30,590.11, 222,048.68) | 450.37 (127.72, 952.49) | 0.24 (0.20, 0.29) |
| Jordan | 664.85 (168.13, 1397.11) | 594.84 (152.24, 1249.31) | 3767.79 (950.81, 7978.46) | 587.89 (150.94, 1242.45) | −0.03 (−0.03, −0.02) |
| Kazakhstan | 7641.22 (2010.57, 16,383.37) | 581.94 (153.10, 1250.76) | 11,198.10 (3003.59, 24,075.54) | 577.67 (154.66, 1244.87) | −0.01 (−0.01, 0.00) |
| Kenya | 3259.60 (1045.00, 6581.71) | 456.54 (146.56, 921.22) | 9530.81 (3065.12, 19,246.14) | 456.24 (146.74, 921.70) | 0.02 (0.01, 0.03) |
| Kiribati | 19.31 (3.35, 43.29) | 553.77 (96.30, 1243.58) | 42.46 (7.12, 95.15) | 553.02 (94.11, 1241.69) | −0.02 (−0.02, −0.01) |
| Kuwait | 211.66 (52.59, 452.31) | 587.78 (149.28, 1248.38) | 1230.72 (298.58, 2615.51) | 572.29 (144.38, 1213.23) | −0.10 (−0.11, −0.09) |
| Kyrgyzstan | 1848.73 (490.29, 3964.94) | 586.00 (155.09, 1259.13) | 3007.43 (794.50, 6489.07) | 586.25 (154.81, 1268.17) | 0.01 (0.01, 0.02) |
| Laos | 1159.05 (179.86, 2565.48) | 609.43 (95.91, 1352.13) | 2592.66 (404.63, 5867.24) | 609.83 (96.91, 1382.54) | 0.01 (0.00, 0.02) |
| Latvia | 2730.38 (993.87, 5200.15) | 700.23 (255.14, 1332.74) | 2549.68 (926.92, 4933.91) | 699.44 (256.11, 1343.50) | 0.01 (0.01, 0.02) |
| Lebanon | 1200.11 (302.17, 2513.14) | 597.99 (151.82, 1253.95) | 3080.79 (776.91, 6433.80) | 589.22 (148.09, 1228.59) | −0.03 (−0.04, −0.03) |
| Lesotho | 465.74 (121.82, 978.31) | 512.41 (134.36, 1076.49) | 583.24 (154.66, 1219.97) | 497.06 (131.87, 1040.86) | −0.10 (−0.11, −0.09) |
| Liberia | 482.50 (115.83, 1021.86) | 549.43 (131.74, 1163.76) | 936.20 (222.91, 1986.37) | 548.29 (132.69, 1164.96) | 0.01 (0.00, 0.01) |
| Libya | 917.19 (230.79, 1928.00) | 602.75 (152.75, 1267.88) | 2620.43 (639.58, 5478.94) | 591.89 (146.66, 1238.11) | −0.06 (−0.06, −0.05) |
| Lithuania | 3119.40 (1084.80, 6086.39) | 644.67 (223.57, 1257.81) | 3466.80 (1211.63, 6820.22) | 642.69 (226.08, 1254.79) | −0.04 (−0.05, −0.02) |
| Luxembourg | 322.75 (93.24, 648.13) | 635.83 (181.21, 1276.91) | 556.09 (158.33, 1129.85) | 636.37 (179.92, 1292.54) | −0.01 (−0.04, 0.01) |
| Macedonia | 1152.10 (343.24, 2458.88) | 632.75 (188.50, 1351.07) | 1935.00 (573.22, 4134.71) | 626.21 (185.33, 1337.79) | −0.02 (−0.02, −0.01) |
| Madagascar | 1853.34 (583.25, 3813.97) | 419.27 (132.49, 862.21) | 4338.48 (1369.16, 8875.91) | 419.20 (132.84, 858.77) | 0.02 (0.01, 0.03) |
| Malawi | 1477.40 (463.73, 3063.61) | 416.74 (131.17, 864.41) | 2825.86 (903.34, 5806.46) | 418.14 (133.74, 859.76) | 0.04 (0.03, 0.05) |
| Malaysia | 4166.89 (766.63, 9150.00) | 525.78 (97.10, 1155.63) | 13,655.75 (2445.77, 29,835.87) | 530.31 (95.64, 1160.36) | 0.06 (0.04, 0.07) |
| Maldives | 42.41 (6.65, 94.39) | 608.56 (97.56, 1364.83) | 162.03 (25.29, 361.84) | 609.58 (96.78, 1369.25) | 0.02 (0.01, 0.03) |
| Mali | 1976.24 (473.90, 4251.57) | 561.13 (136.00, 1209.10) | 4166.04 (1003.13, 8831.50) | 559.86 (135.95, 1188.55) | 0.00 (0.00, 0.01) |
| Malta | 254.63 (62.86, 535.93) | 639.76 (156.86, 1347.21) | 473.68 (117.19, 1017.75) | 639.75 (153.78, 1373.08) | 0.00 (0.00, 0.00) |
| Marshall Islands | 7.52 (1.28, 16.92) | 559.39 (94.94, 1258.46) | 17.76 (3.07, 39.71) | 549.40 (96.23, 1233.68) | −0.06 (−0.06, −0.05) |
| Mauritania | 501.17 (119.87, 1086.45) | 567.47 (136.24, 1228.95) | 1066.11 (251.46, 2288.48) | 568.39 (135.19, 1219.42) | 0.01 (0.01, 0.02) |
| Mauritius | 412.47 (66.22, 916.06) | 606.79 (97.50, 1349.50) | 1109.77 (176.48, 2488.00) | 600.01 (95.49, 1346.29) | −0.03 (−0.03, −0.02) |
| Mexico | 20,819.28 (4980.68, 44,519.66) | 555.15 (133.91, 1186.84) | 66,330.35 (15,632.89, 141,852.50) | 562.16 (133.17, 1202.04) | 0.06 (0.05, 0.07) |
| Moldova | 3332.50 (1220.87, 6434.47) | 700.69 (256.30, 1356.60) | 4303.60 (1598.39, 8308.55) | 699.03 (259.77, 1349.31) | 0.01 (0.00, 0.01) |
| Monaco | 34.97 (8.84, 74.70) | 645.08 (156.94, 1380.79) | 47.21 (11.41, 101.32) | 641.26 (151.62, 1377.66) | −0.02 (−0.02, −0.01) |
| Mongolia | 565.39 (149.97, 1216.92) | 586.06 (156.06, 1260.59) | 1450.62 (388.26, 3116.92) | 584.61 (157.31, 1259.48) | 0.00 (0.00, 0.01) |
| Montenegro | 393.79 (116.39, 839.32) | 638.23 (188.88, 1361.07) | 590.14 (176.20, 1245.38) | 631.07 (187.63, 1329.64) | −0.03 (−0.03, −0.02) |
| Morocco | 7246.45 (1809.11, 15,098.80) | 598.73 (150.25, 1248.67) | 18,465.87 (4734.42, 38,585.25) | 585.92 (151.26, 1224.94) | −0.06 (−0.07, −0.06) |
| Mozambique | 2204.88 (676.84, 4533.15) | 413.17 (127.39, 848.37) | 4293.58 (1365.69, 8843.85) | 412.01 (131.40, 847.42) | 0.02 (0.00, 0.03) |
| Myanmar | 13,452.95 (2164.57, 30,001.90) | 609.37 (98.90, 1361.14) | 30,495.00 (4931.14, 68,360.84) | 606.96 (98.61, 1363.40) | 0.00 (0.00, 0.01) |
| Namibia | 322.22 (82.85, 680.35) | 514.14 (132.18, 1086.96) | 701.01 (184.13, 1493.06) | 507.58 (133.96, 1080.17) | −0.03 (−0.04, −0.03) |
| Nauru | 1.91 (0.33, 4.26) | 561.02 (96.29, 1257.59) | 3.04 (0.53, 6.80) | 551.09 (97.06, 1236.70) | −0.05 (−0.05, −0.04) |
| Nepal | 4498.40 (962.92, 9746.18) | 565.57 (122.92, 1226.64) | 12,252.34 (2518.34, 27,178.74) | 569.01 (117.68, 1262.38) | 0.04 (0.03, 0.04) |
| Netherlands | 10,103.34 (2306.66, 21,648.68) | 583.39 (130.45, 1250.99) | 17,032.43 (3811.33, 36,711.52) | 603.81 (133.33, 1299.15) | 0.21 (0.17, 0.25) |
| New Zealand | 1790.33 (482.25, 3765.65) | 530.44 (141.01, 1116.96) | 3823.31 (1002.93, 8127.13) | 534.13 (138.87, 1136.95) | 0.01 (0.00, 0.02) |
| Nicaragua | 747.39 (169.77, 1601.63) | 553.49 (126.86, 1185.33) | 2513.76 (561.67, 5468.95) | 546.58 (122.69, 1188.53) | −0.02 (−0.03, −0.01) |
| Niger | 1224.56 (291.95, 2624.15) | 565.03 (135.74, 1212.10) | 4307.99 (1015.68, 9296.19) | 565.37 (135.25, 1217.58) | 0.01 (0.00, 0.01) |
| Nigeria | 20,329.65 (5042.04, 43,310.32) | 595.10 (147.10, 1268.15) | 53,933.63 (13,125.40, 114,029.59) | 598.92 (147.99, 1266.65) | 0.03 (0.03, 0.04) |
| Niue | 1.08 (0.18, 2.40) | 562.93 (94.59, 1255.64) | 1.15 (0.20, 2.55) | 553.77 (97.28, 1230.45) | −0.04 (−0.05, −0.04) |
| North Korea | 9119.68 (1460.76, 20,307.44) | 525.31 (84.72, 1171.62) | 17,068.14 (2811.44, 38,150.12) | 523.75 (86.20, 1170.58) | 0.00 (0.00, 0.01) |
| Northern Mariana Islands | 5.89 (0.99, 13.24) | 567.37 (97.67, 1280.03) | 30.50 (5.12, 68.46) | 561.67 (97.40, 1261.65) | −0.02 (−0.03, −0.02) |
| Norway | 3261.38 (745.79, 7057.93) | 615.14 (136.55, 1332.25) | 4826.54 (1181.30, 10,373.62) | 629.79 (152.36, 1354.66) | 0.07 (0.05, 0.09) |
| Oman | 280.73 (68.92, 586.64) | 597.68 (147.15, 1249.25) | 861.10 (213.82, 1811.06) | 595.39 (149.27, 1254.12) | −0.01 (−0.01, 0.00) |
| Pakistan | 22,331.30 (4733.07, 48,715.12) | 537.95 (114.97, 1172.64) | 51,280.06 (9619.79, 114,156.09) | 517.03 (99.50, 1147.04) | −0.16 (−0.19, −0.13) |
| Palau | 4.68 (0.80, 10.45) | 560.83 (96.12, 1255.13) | 12.52 (2.16, 28.08) | 551.47 (96.37, 1238.62) | −0.06 (−0.06, −0.05) |
| Palestine | 500.59 (124.49, 1040.27) | 600.62 (150.58, 1250.23) | 1369.15 (347.98, 2857.07) | 590.36 (152.64, 1233.94) | −0.04 (−0.05, −0.04) |
| Panama | 668.15 (149.37, 1434.14) | 554.53 (124.26, 1190.16) | 2082.55 (468.48, 4545.82) | 549.82 (123.77, 1199.51) | −0.02 (−0.02, −0.01) |
| Papua New Guinea | 862.19 (145.48, 1923.68) | 558.77 (95.22, 1250.22) | 2313.91 (391.59, 5203.10) | 557.16 (95.83, 1253.24) | −0.01 (−0.01, 0.00) |
| Paraguay | 1068.91 (218.70, 2348.64) | 559.67 (114.70, 1229.69) | 2903.46 (578.06, 6564.17) | 554.47 (110.91, 1253.03) | −0.02 (−0.03, −0.02) |
| Peru | 3847.96 (1261.82, 7830.97) | 372.97 (122.74, 758.42) | 11,995.02 (3522.91, 25,073.99) | 411.80 (121.20, 860.90) | 0.35 (0.25, 0.45) |
| Philippines | 16,560.74 (2790.29, 37,057.11) | 630.10 (107.18, 1411.65) | 49,297.77 (8299.15, 110,071.36) | 629.44 (106.59, 1407.76) | 0.00 (0.00, 0.01) |
| Poland | 29,577.20 (9067.32, 61,460.16) | 663.10 (203.01, 1377.42) | 42,591.49 (13,194.88, 88,337.62) | 666.25 (205.29, 1381.98) | 0.03 (0.02, 0.04) |
| Portugal | 8462.63 (2052.54, 17,930.93) | 635.76 (152.45, 1347.48) | 12,285.29 (3080.82, 26,157.97) | 635.39 (155.22, 1353.30) | 0.00 (0.00, 0.01) |
| Puerto Rico | 1732.15 (413.92, 3754.46) | 539.28 (127.83, 1168.42) | 3162.94 (734.88, 6870.61) | 534.21 (121.99, 1156.13) | −0.03 (−0.03, −0.02) |
| Qatar | 33.02 (8.30, 69.28) | 592.73 (153.08, 1245.18) | 317.94 (79.79, 665.20) | 580.88 (150.73, 1212.16) | −0.06 (−0.07, −0.05) |
| Romania | 17,918.31 (5324.06, 38,085.05) | 634.54 (188.21, 1348.21) | 19,726.59 (5991.98, 42,317.44) | 635.89 (191.45, 1365.34) | 0.02 (0.02, 0.03) |
| Russian Federation | 160,267.22 (63,293.66, 301,346.31) | 785.62 (310.03, 1478.75) | 197,798.28 (78,439.46, 371,740.42) | 782.16 (309.13, 1471.85) | 0.06 (−0.02, 0.15) |
| Rwanda | 1166.53 (380.82, 2408.07) | 419.48 (136.74, 865.70) | 2712.95 (869.20, 5656.47) | 419.99 (134.83, 875.91) | 0.03 (0.02, 0.05) |
| Saint Kitts and Nevis | 18.12 (4.35, 39.16) | 534.51 (126.15, 1154.57) | 38.35 (8.99, 82.97) | 533.26 (125.85, 1154.48) | −0.01 (−0.01, 0.00) |
| Saint Lucia | 43.00 (9.96, 93.43) | 533.11 (122.34, 1158.73) | 117.00 (26.51, 250.68) | 530.53 (120.64, 1136.76) | 0.00 (−0.01, 0.00) |
| Saint Vincent and the Grenadines | 35.06 (8.09, 75.88) | 536.16 (122.30, 1159.97) | 66.39 (15.78, 143.27) | 531.92 (126.56, 1148.32) | −0.02 (−0.03, −0.02) |
| Samoa | 42.33 (7.28, 95.17) | 562.20 (97.22, 1265.24) | 71.36 (12.15, 158.79) | 554.21 (95.05, 1234.41) | −0.04 (−0.04, −0.04) |
| San Marino | 18.95 (4.57, 40.46) | 641.63 (152.14, 1370.20) | 36.45 (8.83, 78.78) | 639.55 (152.99, 1381.79) | −0.01 (−0.02, −0.01) |
| Sao Tome and Principe | 34.22 (8.46, 72.28) | 569.90 (141.09, 1204.78) | 56.89 (13.96, 122.74) | 566.24 (140.58, 1220.51) | −0.01 (−0.01, −0.01) |
| Saudi Arabia | 2127.36 (561.79, 4423.14) | 557.51 (147.92, 1158.47) | 7599.03 (1982.96, 15,772.33) | 554.11 (148.02, 1149.38) | −0.02 (−0.02, −0.01) |
| Senegal | 1510.06 (357.18, 3267.90) | 563.63 (134.35, 1219.38) | 3945.42 (951.59, 8380.87) | 561.89 (136.76, 1195.29) | 0.00 (−0.01, 0.00) |
| Serbia | 7564.02 (2290.49, 15,936.85) | 635.40 (191.56, 1340.82) | 9110.11 (2838.97, 19,326.03) | 630.28 (195.29, 1336.35) | −0.01 (−0.01, 0.00) |
| Seychelles | 32.31 (5.22, 72.38) | 615.13 (98.62, 1375.39) | 67.09 (10.52, 151.18) | 603.86 (95.70, 1364.31) | −0.06 (−0.07, −0.06) |
| Sierra Leone | 913.46 (221.66, 1966.25) | 563.12 (136.72, 1212.15) | 1729.91 (407.27, 3686.55) | 562.91 (133.82, 1198.02) | 0.00 (0.00, 0.01) |
| Singapore | 690.72 (220.04, 1411.70) | 343.16 (109.98, 701.03) | 2848.68 (864.93, 6034.50) | 373.51 (113.30, 791.57) | 0.32 (0.24, 0.39) |
| Slovakia | 3718.60 (1127.09, 7963.25) | 632.17 (190.35, 1352.36) | 5550.45 (1681.88, 11,897.80) | 631.82 (190.57, 1353.28) | 0.01 (0.00, 0.01) |
| Slovenia | 1608.49 (468.42, 3407.52) | 629.17 (182.99, 1332.66) | 2282.49 (669.41, 4838.98) | 631.17 (184.28, 1337.06) | 0.02 (0.01, 0.02) |
| Solomon Islands | 61.32 (10.18, 140.94) | 560.35 (94.91, 1285.09) | 160.39 (26.78, 358.65) | 556.48 (94.19, 1245.21) | −0.02 (−0.03, −0.02) |
| Somalia | 877.23 (275.91, 1820.73) | 419.52 (133.04, 867.02) | 2498.96 (784.63, 5169.13) | 417.15 (131.02, 863.22) | 0.00 (−0.01, 0.01) |
| South Africa | 10,937.05 (2940.91, 23,093.66) | 545.65 (146.97, 1152.22) | 25,367.72 (6829.06, 53,545.37) | 535.58 (144.57, 1130.14) | −0.05 (−0.06, −0.05) |
| South Korea | 14,007.51 (3445.31, 30,578.44) | 459.00 (114.06, 1000.44) | 39,407.00 (9971.08, 84,597.25) | 451.31 (113.77, 969.82) | −0.13 (−0.16, −0.10) |
| South Sudan | 676.46 (213.35, 1386.86) | 408.32 (128.72, 837.21) | 1292.80 (408.68, 2672.26) | 409.69 (130.58, 845.62) | 0.02 (0.01, 0.03) |
| Spain | 36,888.89 (10,440.11, 75,601.21) | 720.19 (200.71, 1478.44) | 53,074.18 (14,493.24, 111,283.60) | 697.91 (187.54, 1465.80) | −0.05 (−0.09, −0.01) |
| Sri Lanka | 5677.73 (891.98, 12,838.50) | 609.83 (96.61, 1380.17) | 16,214.51 (2602.93, 36,972.12) | 602.33 (96.53, 1373.97) | −0.03 (−0.03, −0.03) |
| Sudan | 4284.12 (1097.95, 9014.83) | 599.98 (154.74, 1261.54) | 8905.15 (2190.04, 18,695.77) | 597.10 (149.24, 1253.58) | −0.01 (−0.02, −0.01) |
| Suriname | 124.67 (28.01, 266.64) | 538.31 (122.29, 1153.51) | 323.43 (73.79, 705.29) | 528.41 (121.69, 1153.52) | −0.05 (−0.05, −0.04) |
| Swaziland | 133.26 (35.16, 279.50) | 513.19 (135.93, 1076.02) | 290.08 (75.89, 606.85) | 499.79 (131.25, 1045.35) | −0.08 (−0.09, −0.07) |
| Sweden | 7907.14 (1760.81, 16,991.03) | 683.89 (148.95, 1471.50) | 10,378.65 (2328.69, 22,927.12) | 671.04 (148.98, 1480.14) | −0.12 (−0.13, −0.10) |
| Switzerland | 5025.84 (1378.75, 10,432.53) | 573.88 (154.23, 1189.23) | 8117.44 (2170.81, 17,069.27) | 575.28 (151.40, 1208.72) | 0.02 (0.01, 0.04) |
| Syria | 2673.97 (665.86, 5593.16) | 599.84 (151.38, 1257.13) | 7373.50 (1827.35, 15,352.32) | 595.05 (149.53, 1240.88) | −0.03 (−0.03, −0.02) |
| Taiwan (Province of China) | 7573.42 (1186.01, 17,114.46) | 578.57 (90.07, 1308.59) | 23,154.44 (3632.63, 52,970.67) | 585.49 (91.52, 1336.97) | 0.07 (0.06, 0.08) |
| Tajikistan | 1597.72 (425.43, 3461.62) | 588.90 (157.08, 1277.59) | 3453.50 (928.08, 7380.17) | 585.25 (158.02, 1258.89) | −0.01 (−0.01, 0.00) |
| Tanzania | 4072.10 (1261.38, 8433.49) | 423.71 (131.56, 878.00) | 9775.64 (2899.19, 20,084.53) | 440.64 (131.10, 905.73) | 0.11 (0.04, 0.18) |
| Thailand | 26,730.35 (3241.78, 61,047.95) | 798.71 (98.16, 1824.55) | 75,691.21 (10,232.82, 173,836.80) | 713.66 (96.65, 1640.88) | −0.39 (−0.50, −0.29) |
| The Bahamas | 74.68 (17.27, 163.55) | 540.84 (125.40, 1184.01) | 219.91 (50.31, 478.56) | 533.94 (123.61, 1162.19) | −0.03 (−0.04, −0.03) |
| The Gambia | 146.22 (35.16, 312.42) | 562.21 (135.77, 1200.67) | 451.52 (110.55, 966.44) | 559.10 (137.41, 1195.95) | −0.01 (−0.02, −0.01) |
| Timor-Leste | 143.12 (22.47, 316.15) | 606.38 (97.63, 1349.26) | 436.40 (68.28, 975.67) | 605.19 (94.09, 1356.66) | 0.02 (0.01, 0.04) |
| Togo | 606.05 (143.46, 1293.97) | 564.68 (135.06, 1205.46) | 2120.92 (505.73, 4522.77) | 565.68 (136.85, 1205.27) | 0.01 (0.00, 0.01) |
| Tokelau | 0.68 (0.12, 1.54) | 561.20 (97.00, 1279.06) | 0.71 (0.12, 1.58) | 554.43 (95.79, 1239.20) | −0.04 (−0.04, −0.03) |
| Tonga | 28.55 (4.83, 64.17) | 564.42 (96.30, 1272.07) | 39.43 (6.81, 88.91) | 557.32 (96.48, 1256.44) | −0.03 (−0.04, −0.03) |
| Trinidad and Tobago | 381.02 (88.68, 823.48) | 533.26 (123.20, 1151.90) | 960.81 (223.24, 2096.07) | 528.49 (122.51, 1152.76) | −0.02 (−0.03, −0.01) |
| Tunisia | 2666.32 (654.04, 5550.95) | 601.81 (149.41, 1254.34) | 7255.10 (1877.86, 15,001.79) | 591.13 (153.56, 1224.46) | −0.04 (−0.04, −0.03) |
| Turkey | 20,810.44 (5462.68, 43,761.45) | 632.35 (167.48, 1329.74) | 55,554.45 (14,263.22, 115,886.42) | 628.19 (161.72, 1309.80) | −0.02 (−0.03, −0.01) |
| Turkmenistan | 1157.16 (307.16, 2498.48) | 587.79 (156.43, 1270.74) | 2508.77 (677.60, 5397.66) | 587.26 (159.04, 1265.89) | 0.01 (0.00, 0.02) |
| Tuvalu | 3.83 (0.67, 8.62) | 563.65 (99.06, 1269.34) | 5.63 (0.97, 12.76) | 557.31 (96.18, 1263.39) | −0.01 (−0.02, 0.01) |
| Uganda | 2316.88 (730.75, 4732.31) | 417.47 (131.83, 853.03) | 5862.73 (1811.15, 12,049.62) | 419.75 (130.42, 862.50) | 0.04 (0.03, 0.04) |
| Ukraine | 56,461.58 (20,682.48, 108,197.57) | 723.63 (264.32, 1385.46) | 57,929.24 (21,757.71, 111,235.65) | 724.41 (272.60, 1388.00) | 0.02 (0.02, 0.03) |
| United Arab Emirates | 132.84 (33.40, 276.73) | 590.89 (150.35, 1233.27) | 1183.19 (293.61, 2522.77) | 584.53 (151.89, 1242.43) | −0.02 (−0.03, −0.01) |
| United Kingdom | 49,292.06 (12,664.88, 102,984.76) | 648.78 (163.14, 1356.38) | 66,133.55 (16,635.47, 141,576.39) | 648.97 (160.98, 1391.76) | 0.01 (0.00, 0.02) |
| United States | 180,934.60 (39,619.84, 402,831.65) | 657.80 (141.19, 1467.44) | 327,753.47 (72,588.30, 722,022.00) | 641.28 (140.38, 1414.23) | −0.03 (−0.10, 0.04) |
| Uruguay | 1750.35 (480.12, 3678.60) | 481.31 (131.09, 1011.05) | 2259.40 (620.62, 4756.61) | 477.27 (129.21, 1005.02) | −0.02 (−0.03, −0.02) |
| Uzbekistan | 6790.46 (1846.60, 14,731.39) | 586.90 (159.46, 1274.32) | 16,013.79 (4308.13, 34,884.73) | 582.97 (156.96, 1271.87) | −0.01 (−0.02, −0.01) |
| Vanuatu | 27.28 (4.60, 61.89) | 563.93 (96.22, 1281.93) | 87.58 (15.16, 200.40) | 558.94 (97.78, 1278.28) | −0.03 (−0.04, −0.03) |
| Venezuela | 4657.38 (1053.59, 9901.69) | 551.77 (125.50, 1172.41) | 15,817.41 (3510.23, 34,339.82) | 553.27 (123.02, 1201.52) | 0.02 (0.01, 0.03) |
| Vietnam | 25,141.40 (4144.54, 56,864.03) | 617.12 (101.93, 1396.60) | 62,854.29 (10,069.18, 141,755.13) | 615.34 (99.08, 1389.70) | 0.00 (0.00, 0.01) |
| Virgin Islands, U.S. | 41.46 (9.33, 89.11) | 537.66 (122.01, 1156.59) | 87.44 (20.31, 188.93) | 529.90 (121.58, 1143.47) | −0.04 (−0.04, −0.03) |
| Yemen | 2514.80 (630.44, 5266.16) | 591.47 (149.27, 1240.09) | 7230.86 (1790.44, 15,026.08) | 592.01 (147.93, 1232.94) | 0.00 (−0.01, 0.00) |
| Zambia | 1082.71 (358.09, 2168.84) | 467.21 (155.63, 934.54) | 2810.90 (929.63, 5612.49) | 470.49 (156.09, 939.91) | 0.02 (0.01, 0.04) |
| Zimbabwe | 1762.60 (463.90, 3738.21) | 513.84 (135.53, 1089.89) | 3538.82 (926.78, 7367.09) | 505.23 (133.16, 1053.49) | −0.05 (−0.06, −0.04) |

ASR: age-standardized rate; EAPC: estimated annual percentage change; CI: confidence interval.

**Supplementary Fig. 2. The EAPC in the age-standardized rate (ASIR) of migraine among menopausal women across 204 countries and territories worldwide in 2021.** (A) ASPR. (B) ASIR. (C) ASDR. ASPR: age-standardized prevalence rate; ASIR: age-standardized incidence rate; ASDR: age-standardized disability-adjusted life years rate; EAPC: estimated annual percentage change.

**Supplementary Fig. 3. Age-standardized prevalence rates (ASPR) of migraine among postmenopausal women across different age groups, stratified by SDI regions and the GBD regions.** SDI: socio-demographic index.

**Supplementary Fig. 4. Age-standardized incidence rates (ASIR) of migraine among postmenopausal women across different age groups, stratified by SDI regions and the GBD regions.** SDI: socio-demographic index.

**Supplementary Fig. 5. Age-standardized DALYs rates (ASDR) of migraine among postmenopausal women across different age groups, stratified by SDI regions and the GBD regions.** SDI: socio-demographic index.

**Supplementary Fig. 6. Population-level determinants of changes in aging, population growth, and epidemiological trends contributing to migraine-related disability-adjusted life years (DALYs) among postmenopausal women globally and across Socio-demographic Index (SDI) regions from 1990 to 2021.** SDI: socio-demographic index.
